# Critical analysis of translational potential of rodent models of white matter pathology across a wide spectrum of human diseases

**DOI:** 10.1038/s41419-025-07893-6

**Published:** 2025-07-31

**Authors:** Wenxuan Zhou, Shiyue Xia, Chenmeng Wang, Qingwu Yang, Alexei Verkhratsky, Jianqin Niu

**Affiliations:** 1 Department of Histology and Embryology, Third Military Medical University, Chongqing, 400038 China; 2 Research Centre, Seventh Affiliated Hospital of Sun Yat-sen University, Shenzhen, 518107 China; 3 Department of Neurology, Second Affiliated Hospital, Chongqing Institute for Brain and Intelligence, Third Military Medical University, Chongqing, 400037 China; 4 Faculty of Biology, Medicine and Health, The University of Manchester, Manchester, M139PL UK; 5 Department of Neurosciences, University of the Basque Country, CIBERNED, Leioa 48940, and IKERBASQUE Basque Foundation for Science, Bilbao, Spain; 6 International Collaborative Center on Big Science Plan for Purinergic Signalling, Chengdu University of Traditional Chinese Medicine, Chengdu, China; 7 Celica Biomedical, Tehnološki park 24, 1000 Ljubljana, Slovenia; 8 Department of Forensic Analytical Toxicology, School of Forensic Medicine, China Medical University, Shenyang, 110122 China; 9 Chongqing Key Laboratory of Neurobiology, Chongqing, 400038 China; 10 Key Laboratory of Major Brain Disease and Aging Research, Chongqing Medical University, Chongqing, 400016 China; 11 State Key Laboratory of Trauma and Chemical Poisoning, Chongqing, 400042 China

**Keywords:** Glial biology, Myelin biology and repair

## Abstract

Rodents are the most commonly used laboratory animals in medical research. However, significant evolutionary divergences between humans and rodents, particularly in the complexity of white matter connectome, which are fundamentally shaped by myelin as their major structural component, pose critical challenges in modeling the human neurological diseases. Given the divergences and central roles of myelin in pathology, a thorough reevaluation of the rodent models used in contemporary research is critical, alongside the careful selection, optimization, or de novo development of models that faithfully recapitulate human white matter disorders. In this review, we summarize the strengths and limitations of existing rodent models, emphasizing their contributions to understanding demyelinating pathologies across autoimmune, neurodegenerative, vascular, perinatal, traumatic, infectious and genetic diseases. We also overview white mater disease models using other species and human stem cells. Subsequently we discuss critical interspecies differences in white matter biology that may limit translational relevance, while highlighting how rodent models enhance our comprehension of various pathological conditions. Lastly, we outline strategies to refine rodent models through advanced genetic engineering, humanized microenvironments, and multimodal phenotyping, with the goal of progressively improving existing them to increase their preclinical translational potentials.

## Facts

- White matter injury is a hallmark of various neurological disorders, impairing neural signal transmission and resulting in motor, sensory and cognitive deficits.
- The pathogenesis and progression of human white matter injuries are highly heterogeneous and not fully reproducible in animal models, posing significant challenges for disease modeling and driving ongoing research efforts worldwide.
- Reevaluation, alongside careful selection, optimization or development of rodent models is essential for studying white matter pathology across human diseases, particularly when exploring potential therapeutic drugs and strategies.

## Open Questions

- To what extent can current rodent models faithfully recapitulate the pathophysiology of human diseases?
- What are the key advantages and limitations of rodent models in translational research?
- How can we enhance the fidelity of rodent models to better mirror human disease mechanisms in future investigations?

## Introduction

In 1659, Robert Hooke and Robert Boyle placed a mouse under an air pump and observed that it felt unconscious when the air pressure was reduced, then this mouse was revived once the air pressure was restored [1]. It was the first documented use of a rodent in scientific research [2, 3]. In 1908, William Castle established Harvard’s Bussey Institution, which became a starting point for many early mouse geneticists. Between 1914 and 1919, Abbie Lathrop (who started farming mice in 1900 in her farm in Pennsylvania) sent mice with tumors to Leo Loeb at the University of Pennsylvania, where cancer studies were conducted [4]. Since that time, myriads of rodent models were created, characterized, and optimized. It is estimated that rodent models, primarily *Mus musculus* (house mice) and *Rattus norvegicus* (Norway rats), represent about 95% of all animal models in neuroscience studies for human diseases [5]. These models provide researchers with a controllable pathogenetic condition, which is important for understanding the pathogenesis, eliminating extraneous factors in diseases, and then focusing on one or several factors alone, particularly in explorations of potential therapeutic drugs and strategies.

**Fig. 1:**
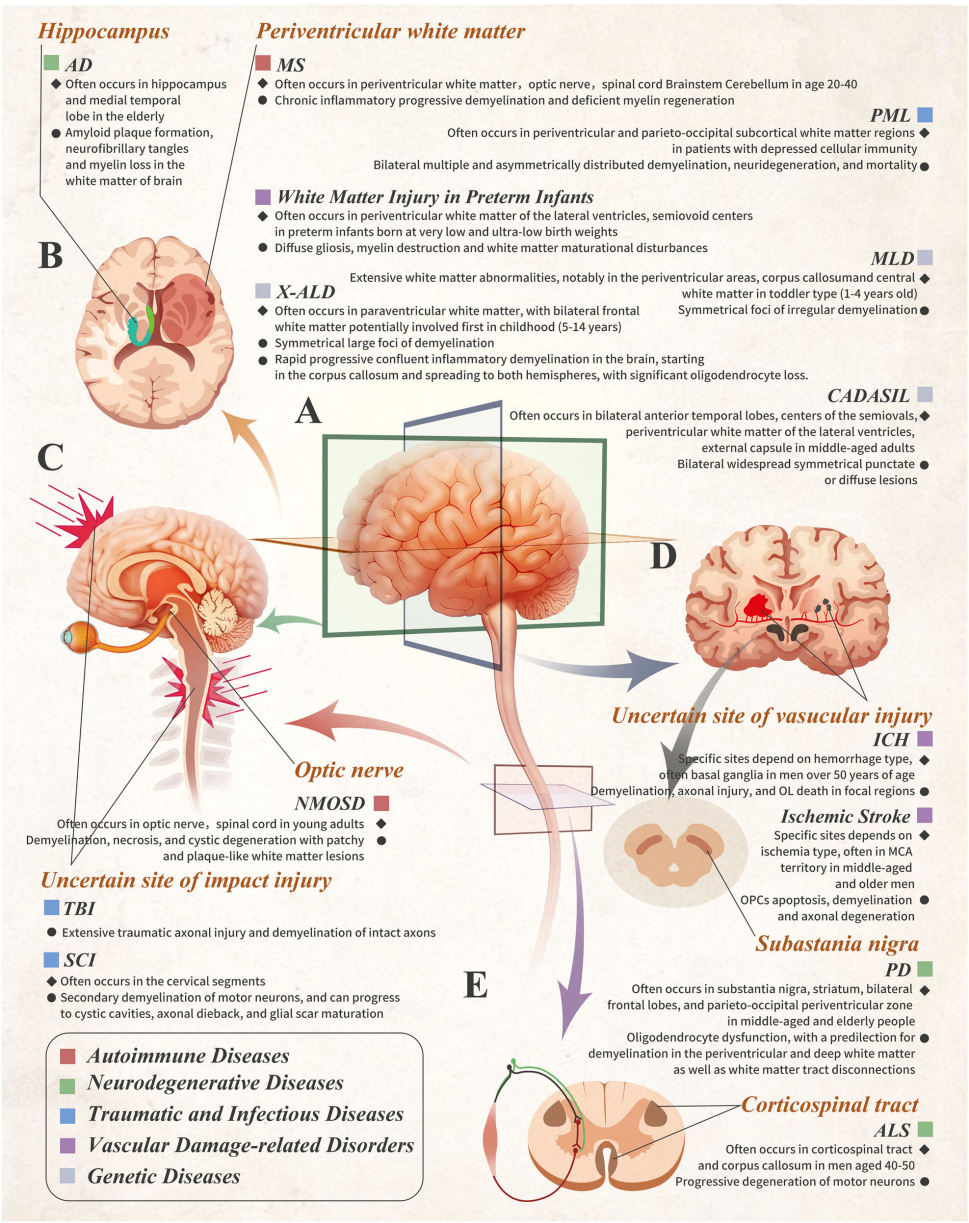
Characteristics of human white matter injury diseases. **A** The indication for brain and spinal cord profiles in various directions. **B**–**E** Cross-sectional views of the brain and spinal cord in different diseases. The accompanying texts provide information about the characteristics of diseases. The figure was drawn by authors for this paper.

**Fig. 2:**
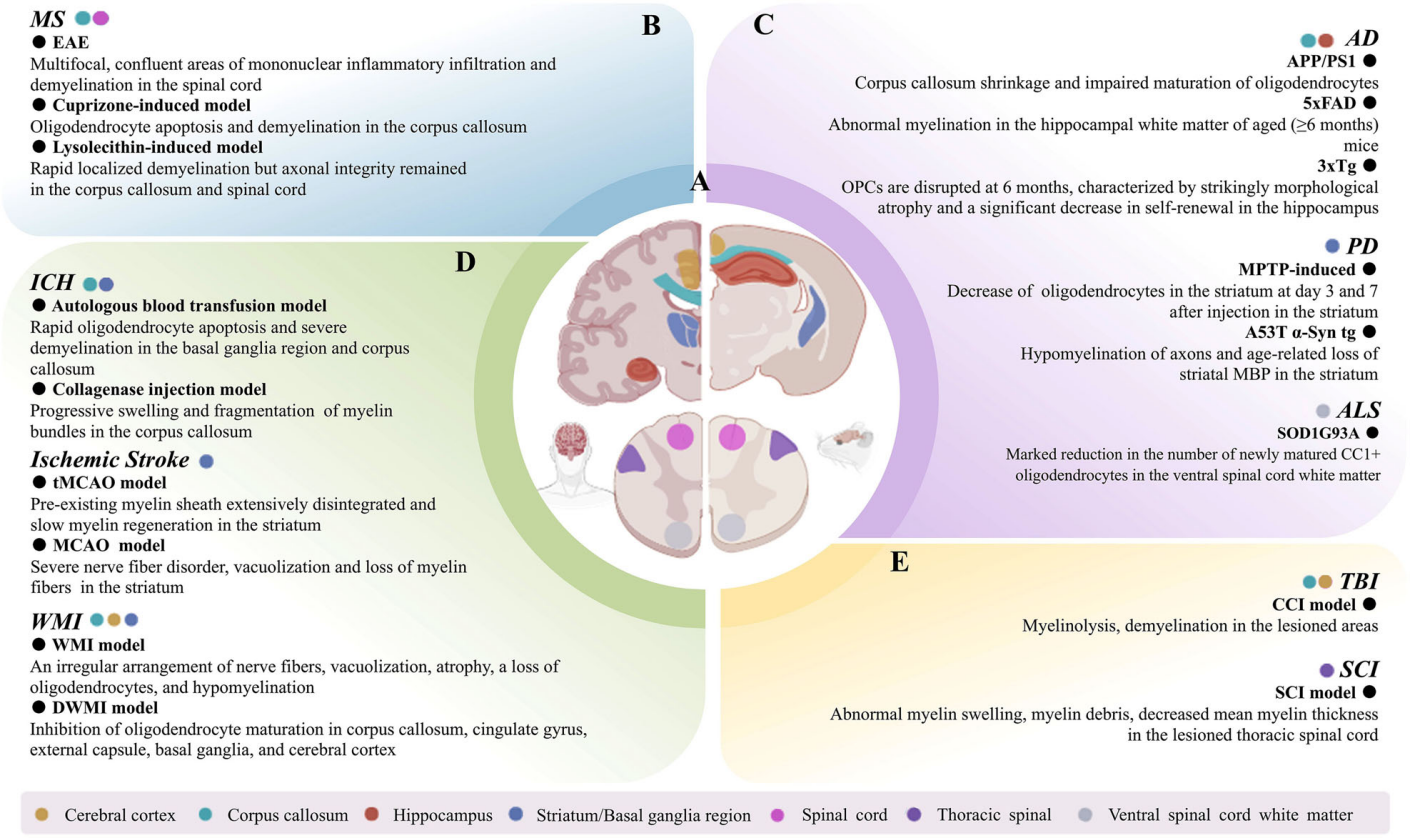
Characteristics of white matter injuries represented by current rodent models. **A** Coronal section of the brain and cross section of the spinal cord in both human and mouse. **B**–**E** The accompanying texts provide information of myelin injuries and description of rodent models employed in current research. The colored dots indicate various injury sites, marked on the sections of the brain and spinal cord.The figure was drawn by authors for this paper.

However, human diseases are diverse. Their idiosyncratic pathogenesis and progression are never fully reproducible in small animal models. In particular, the structural, developmental, and functional complexities of the central nervous system (CNS) are the most different between these two species. Human brain is characterized by highly elaborated connectome; white matter occupies ~50% of the whole volume of the human brain compared to only ~12% in rodents, with the total length of myelinated fibers in the brain of adult men ~15,0000 - 180,000 kilometers [6, 7]. This remarkable evolutionary advance [8, 9], enables humans to engage in complex behaviors, experience a wide range of emotions, consciousness, and other higher cognitive functions [10]. In this review, we aim to address these questions, specifically focusing on white matter diseases related to immunological condition, degeneration, injury, vascular damage, and heredity disorders **(**Fig. 1**)**, we shall provide a succinct summary of rodent models **(**Fig. 2**)**, and outline methodologies used in creation of these models **(**Table 1**)**.

**Table 1:** Characteristics and methodologies for constructing rodent models pertaining to white matter diseases.

	Disease	Model	Animal	Advantage	Disadvantage	Lesion site	Construction method
Autoimmune Diseases	MS	MOG_35-55_ model	C57BL/6 mice	(1) Most widely used EAE model (2) Multiple immune phenotypes of MS	(1) Excessive demyelination and axonal degeneration (2) Wide distribution of lesions (3) Bad recovery from motor deficit	Spinal cord	MOG_35-55_ emulsified with CFA was injected subcutaneously into mice [82]
		Cuprizone-induced model	C57BL/6 mice	(1) Stable reproducibility (2) Easy to observe remyelination	(1) Lack of inflammatory microenvironment (2) Lack of motor dysfunction	Corpus callosum	Introduction of a low dose of Cuprizone into the diet of mice fed continuously for a certain period of time [82]
		Lysolecithin-induced model	C57BL/6 mice	(1) Direct toxic effects (2) Precise control of time and site of injury (3) High axonal integrity	(1) Lack of inflammatory microenvironment (2) Lack of motor dysfunction	Spinal cord, corpus callosum	Using stereotactic techniques, 1% LPC was injected into specific areas of the white matter tracts [104]
	NMOSD	Passive transfer in EAE model	C57BL/6 mice	(1) Comprehensive model for reproduction of typical NMOSD features	(1) Huge demand for NMO-IgG (2) Small lesions around the injection site	Spinal cord, optic nerve	EAE mice were constructed with MOG_35-55_, followed by intraperitoneal injection of AQP4-IgG from NMOSD patients [121]
		Injection model	AQP4-null (CD1) mice	(1) Easy to construct (2) Used to study the role of the complement system in NMOSD	(1) Dependent on human complements	Corpus callosum, external capsule	AQP4-IgG (IgG purified from NMOSD patient serum) and human complement (non-heat-inactivated human serum) were injected directly into the mice brain [123]
Neurodegenerative Diseases	AD	APP/PS1 model	C57BL/6 J mice	(1) Effectively replicating Aβ plaque formation	(1) Iron Metabolism, inflammation, and amyloid morphology differ from AD patients	Corpus callosum, hippocampus	Genetic mutations: APP, PSEN1 [442]
		3xTg model	C57BL/6, 129×1/SvJ, 129S1/Sv mice	(1) Replicating multiple abnormal anatomical structures in AD pathology (2) Accurately reflecting cognitive deficits in memory and learning in AD patients	(1) Relatively long experimental period (2) Instability of gene expression	Hippocampus	Genetic mutations: PSEN1, APP, MAPT [166]
		5xFAD model	C57BL/6 mice	(1) Early manifestations of neuroinflammation and neurodegeneration	(1) High experimental complexity and cost	Hippocampus	Genetic mutations: APP, PSEN1 [163]
	PD	6-OHDA model	Sprague-Dawley rat	(1) Precise control of site of injection (2) Production of major behavioral deficits seen in PD patients	(1) Failure to reflect the key neuropathologic feature of Lewy body formation	Nigra, striatum	Implementation of brain stereotactic techniques in substantia nigra regions for precise injection of 6-OHDA into Sprague-Dawley rats [189]
		MPTP model	C57BL/6 mice	(1) Penetration of the blood-brain barrier (2) Suitable for the study of mitochondrial dysfunction in PD (3) Easy to construct	(1) Failure to reflect the key neuropathologic feature of Lewy body formation	Nigra, striatum	Intraperitoneal injection of MPTP into C57BL/6 mice [182]
		A53T aSyn Tg Mouse	C57BL/6 mice	(1) Effectively simulate the abnormal aggregation of α-synuclein in PD	(1) No significant dopaminergic neuronal degeneration	Striatum	Genetic mutations: SNCA A53T [198]
	ALS	SOD1-G93A	C57BL/6/SJL	(1) Similarity of motor behavioral and pathological changes with ALS patients	(1) No obverse degeneration of motor neurons	Ventral spinal cord white matter	Genetic mutations: SOD1 G93A [223]
Vascular Damage-related Disorders	Ischemic Stroke	tMCAO model	Sprague-Dawley rat, C57BL/6 J mice	(1) Easy to construct (2) Brain damage is localized and reversible	(1) Diversities of injury location and severity	Striatum	A removable suture is inserted through the external carotid artery to unilaterally block the middle cerebral artery [237]
		MCAO model	C57BL/6 mice	(1) Effectively simulate the pathological process of clinical large artery ischemia occlusion (2) Suitable for studying the long-term pathological changes after cerebral ischemia	(1) Failed to simulate reperfusion injury (2) high animal mortality	Striatum	A suture is inserted through the external carotid artery to unilaterally block the middle cerebral artery [443]
	ICH	Autologous blood injection model	Sprague-Dawley rat, C57BL/6 J mice	(1) Easy to construct (2) Brain damage is localized and reversible	(1) There is a time-dependent trend in recovery (2) Hematoma formation is independent of vascular injury	Basal ganglia region, corpus callosum	Non-heparinised autologous animal blood was Injected into specific brain regions [444]
		Collagenase injection model	Sprague-Dawley rat, C57BL/6 J mice	(1) Effectively replicate the fundamental pathological alterations (2) Precisely mirrors the clinical phases of hemorrhagic stroke	(1) Overdose of collagenase can easily kill animals	Corpus callosum	Direct injection of collagenase type IV into specific areas within the brain parenchyma of animals [269]
	WMI in Preterm Infants	WMI model	P3 Sprague-Dawley rat	(1) reproduces injuries in preterm infants at 24–28 weeks’ gestation	(1) Hard to construct	Corpus callosum	Unilateral common carotid artery ligation and a 10-minute hypoxic (6% oxygen) [288]
		DWMI model	OF1 mice	(1) Effectively simulate oligodendrocyte maturation disorders (2) elucidates the relationship between systemic inflammation, myelin formation, and WMI	(1) Hard to construct (2) Lack of hypoxic intervention	Corpus callosum, basal ganglia, and cerebral cortex	Hypoxia, and inflammation induced by intraperitoneal injection of IL-1β for 5 consecutive days [292]
Traumatic and Infectious Diseases	TBI	CCI model	C57BL/6 J mice	(1) Easy to construct (2) High reproducibility and stability	(1) Craniotomy required (2) Pneumatic or electromagnetic drive device required	Cerebral cortex, corpus callosum	Injury is induced to exposed mouse dura mater after craniotomy using a mechanical insult device [302]
	SCI	Contusion SCI model	Sprague-Dawley rat	(1) Easy to construct and low cost (2) High similarities in mechanical insult and clinical manifestations	(1) variations across the sites of injury	Thoracic spinal	Weight drop method, electromagnetic strikers, and air gun projectiles can be used to induce the injuries [326, 330, 331]
	PML	JCPyV model	C57BL/6 mice	(1) Easy to construct (2) Effectively simulate JCPyV CNS invasion	(1) Using non-JCPyV infection (2) Failed to simulate JCPyV replication	Corpus callosum	Intracranial inoculation of MuPyV into C57BL/6 mice [341]
Genetic Diseases	X-ALD	cALD model	C57BL/6 J mice	(1) Accurately replicates the core pathological features of human cALD	(1) Failed to encompass the pathological changes in the chronic phase	Corpus callosum	Deploy a two-hit method combining cuprizone and EAE models in 8-week-old male Abcd1-null mice [358]
	MLD	ASA-deficient mouse model	C57BL/6 J mice	(1) Similar sulfatide storage patterns to human patients	(1) Significantly slower pathological progression (2) Inadequate demyelination in both the CNS and PNS	Corpus callosum, optic nerve	Genetic mutations: ASA [365]
	CADSIL	TgNOTCH3^R169C^ mouse model	FVB/N mice	(1) The only model can exhibit CADASIL-specific early onset of vascular NOTCH3 aggregation with micro-infarcts and subsequent white matter lesions	(1) Behavioral analysis is complicated in the FVB/N genetic background	Corpus callosum, internal capsule	Genetic mutations: Notch3 [372]

## White matter damage in pathogenesis and progression of neurological diseases

White matter, primarily composed of myelin produced by oligodendrocytes enwrapping axons with multilamellar membranous sheath [11], plays a central role in the CNS function. This myelin dominated structure ensures rapid propagation of action potentials, protects axons and supports axonal metabolic activity, structural integrity, and plasticity [12]. White matter is highly vulnerable to pathological insults. Damage to oligodendrocytes or myelin disrupts signal conduction, leading to functional impairments, including motor, sensory, and cognitive deficits [13].

Mounting evidence underscores the broad clinical relevance of white matter injury across diverse diseases [14–17]. Demyelination is a hallmark of autoimmune diseases, neurodegenerative diseases, vascular damage-related diseases, white matter injury in preterm infants, traumatic and infectious diseases and many genetic diseases. A notable example is multiple sclerosis (MS), where autoimmune attack on CNS myelin cause progressive neurological disability [18]. Similarly, neurodegenerative conditions such as Alzheimer’s or Parkinson’s diseases (AD and PD) exhibit a significant decrease in myelin content, correlating with cognitive deterioration [19]. Furthermore, evidence on white matter injury in psychiatric disorders and cognitive development in children are accumulating [20]. During development, inadequate formation of the myelin sheath often leads to significant impairments of learning capabilities, motor functions, and response times. In addition, insufficient myelination in premature infants manifests with dystonia, delayed language development, and cognitive deficits [21].

The population of life-long proliferating oligodendrocyte precursor cells (OPCs), counteracts demyelination by differentiating into oligodendrocytes and remyelinating axonal tracts [22]. However, this endogenous repair system often fails in chronic conditions, resulting in axonal degeneration and irreversible functional loss. Key contributing factors include impaired OPC differentiation, sustained neuroinflammation, and progressive axonal damage [23–25]. These challenges highlight the urgent need to elucidate the mechanisms of (de)remyelination. To achieve this, careful selection, optimization, or novel development of rodent models that faithfully replicate human white matter injury pathologies is essential not only for deciphering disease mechanisms but also for advancing therapeutic strategies.

## White matter vulnerability: cellular and molecular mechanisms

Damage to the white matter is mediated by several factors, including excitotoxicity, metabolic insufficiency of oligodendroglia, imbalanced gene expression, alterations of intercellular interactions, and aberrant microenvironment. Accumulation and synergy of these factors ultimately lead to oligodendroglial death, demyelination, and functional impairment [26–28]. At the cellular level, oligodendroglia is particularly vulnerable to exogenous and endogenous insults. Entry of immune cells into the CNS, for instance, triggers multiple cascades causing oligodendroglial death in autoimmune diseases [29, 30]. Of note, the mechanisms and immune cell subsets underlying demyelination differ between mice and humans [27, 31, 32]. Furthermore, human microglia possess distinct innate immunity, which may affect oligodendroglia [33]. Expression profiles of surface receptors and functional regulation in human microglia are more complex, and their response to injury is subtler and more gradual. This includes a diminished response to dendritic cell differentiation and reduced expression of major histocompatibility complex Class II (MHC II) [34–37]. When adaptive immunity is concerned, rodent – human differences also exist in the composition of T cell and B cell subpopulations, as well as in the mechanisms of immune memory formation [37–39]. In the experimental autoimmune encephalomyelitis (EAE) mouse model for MS, demyelination is primarily mediated by macrophages, CD4 + T cells, and CD8 + T cells, in contrast to the leading role of B cells in orchestrating the demyelination process in humans [40–42].

Aging also contributes to cellular vulnerably, through generation of reactive oxygen species (ROS), lipid peroxidation, and Ca^2+^ overload [43, 44]. Humans lifespan is much longer than rodents, and hence human oligodendroglia face multiple challenges that rodents do not encounter. Prolonged duration of human MS (often spanning decades) creates cumulative cellular stress that short-lived rodent models cannot reproduce [45–47]. To address this issue, experimental models such as cuprizone model in aged mice, were adapted to reflect human conditions [48, 49].

At the molecular level, the metabolic fragility of oligodendroglia presents a key molecular vulnerability [28, 50]. High metabolic demand and limited antioxidant capacity of the white matter render the latter susceptible to oxidative stress and ATP deficiency, which often happen in stroke, MS, and other related disorders [51, 52]. In addition, glutamate excitotoxicity accompanying many pathological states is driven by excessive activation of NMDA and AMPA receptors in both OPCs and mature oligodendrocytes, leading to Ca^2+^ overload, opening of the mitochondrial permeability transition pore, and increased ROS production [53–55]. This in turn activates NADPH oxidases, resulting in damage to lipids, proteins, and DNA, ultimately leading to cell death [56, 57]. Aberrant purinergic signaling also intensifies white matter damage. In cuprizone-induced and microstereotaxic injection of AMPA-induced demyelinating models, massive release of ATP activates P2X_7_ receptors on oligodendrocytes, leading to pathological Ca²⁺ influx and activation of NLRP3 inflammasome activation [58, 59]. In humans, energy deficits arise from mitochondrial malfunction, oxidative stress, and ATP-related neuroinflammation [60]. In contrast, demyelination in rodent models is typically induced through a single metabolic insult such as cuprizone toxicity. Additionally, the lipid-dependent nature of myelin contributes to another human-specific molecular vulnerability. Human MS is characterized by distinct lipid abnormalities, such as deficiencies in polyunsaturated fatty acids [61], which rodent models are unable to reproduce.

Likewise, gene expression changes observed in human demyelination, especially concerning transcription start site selection, mRNA modifications, and non-coding RNA regulation [62, 63], occur differently in rodents due to species-specific transcriptional regulation [64]. Such as variations exist in myelin gene sequences [65, 66] and gene copy numbers [67–69]. Furthermore, while sex hormones do not significantly influence demyelination in the majority of rodent models, there are notable sex differences in humans: for example, MS occurs three times more in women compared to men [70].

Overall, due to the complexities inherent to the disease-model gap between humans and rodents, current rodent models fail to fully capture the intricate molecular and cellular vulnerabilities of human white matter. This restricts the translational potential of research, as evidenced by the failure of many treatments tested on rodents to succeed in human clinical trials [71–74], consequently highlighting the pressing need for more relevant models.

## Rodent models of autoimmune diseases

Immune attack is the major etiological factor in the pathogenesis of MS, neuromyelitis optica spectrum disorders (NMOSD), and acute disseminated encephalomyelitis (ADEM) [75–78]. Although disease-modifying therapies significantly improved immune-oriented therapeutic effects, there is still a notable lack of remyelination-oriented therapies aimed at rebuilding the white matter connectome and restoring neural functions. Rodent models of white matter injuries provide a myelin-focused and space-time identified tool to study the demyelinating and remyelinating of autoimmune diseases.

### Multiple Sclerosis

MS is an autoimmune chronic inflammatory demyelinating disease affecting the CNS, which is classified into three subtypes according to the clinical course: relapsing-remitting MS (RR-MS), secondary progressive MS (SP-MS), and primary progressive MS (PP-MS) [79]. Its primary pathological changes include mild to moderate severity progressive demyelination and deficient myelin regeneration, which often occur in the periventricular white matter, optic nerve, spinal cord, brainstem and cerebellum [80]. Experimental models of MS include the experimental autoimmune encephalomyelitis (EAE) model, the cuprizone model, and the lysolecithin-induced demyelination model.

#### Experimental autoimmune encephalomyelitis (EAE)

The EAE model can be induced either through active immunization with basic myelin proteins, which instigates production of autoantibody against myelin proteins, or by passive transfer of myelin-reactive T cells. The former is referred to as an active EAE, whereas the latter is known as a passive EAE [49, 81]. In murine active EAE models, C57BL/6 mice are immunized with myelin oligodendrocyte glycoprotein (MOG35-55) peptides, SJL mice with myelin proteolipid protein (PLP139-151) peptides, and Lewis rats with myelin basic protein (MBP63-88). Pathological changes differ between these animals. The MOG35-55 model is the most commonly used, producing a chronic, non-relapsing disease that embraces features of all three subtypes of MS. This model shows multifocal, confluent areas of mononuclear inflammatory infiltration and demyelination in the white matter of the spinal cord, along with meningitis and appearance of perivascular inflammatory cuffs in the cerebellum and hindbrain white matter [82–85]. The PLP-EAE mimics RR-MS, featuring prominent perivascular inflammatory infiltration and axonal demyelination. This pathology is characterized by the presence of macrophages containing myelin debris, which typically occurs 12-18 days post-induction [86, 87]. Characteristic lesions appear in the optic nerve, brainstem, spinal cord, cerebellum and cerebral cortex [83]. In addition, MBP-EAE follows a primary progressive course, exhibiting pronounced inflammation in the spinal cord, cerebellum, and brainstem, but only mild demyelination [83, 88]. On the other hand, passive EAE is not associated with extensive primary demyelination. Instead, it mainly presents as prolonged confluent inflammatory lesioning in the brainstem, which is useful to understanding the role of CD4^+^ T cells in the disease [89, 90].

However, there are fundamental differences between the EAE models and human MS. The EAE is primarily mediated by CD4^+^ T-cells, while CD8^+^ T-cells and B-cells play significant roles in MS [91]. Additionally, demyelinating lesions in EAE are more commonly found in the spinal cord, whereas MS predominantly affects the cerebral and cerebellar cortex [92]. Therefore, the development of ‘humanized’ immune mice is crucial for a better understanding of the immunopathogenesis of MS. A patient-derived peripheral blood mononuclear cell (PBMC) transplantation model can create a humanized mouse that replicates several key features of MS. This model exhibits a mixture of human CD4^+^ and CD8^+^ T-cell lesions in the white matter of the brain, particularly in the corpus callosum and optic tract [93]. Furthermore, considering the effects of overactivated Wnt pathway in OPCs in human MS [94, 95], a new mouse model has been developed that combines EAE with excessive Wnt activation, which is induced by the conditional loss of the crucial pathway repressor, adenomatous polyposis coli (APC) in OPCs. This model effectively mimics the immune cell infiltration observed in the brains of patients [29].

#### Cuprizone-induced demyelination

Cuprizone, a copper chelator that acts specifically on mature oligodendrocytes, impairs, when added to the diet of mice, metabolism of oligodendrocytes and trigger their apoptosis, leading to demyelination. To establish this model, C57BL/6 mice are fed with low doses of cuprizone for 4 - 6 weeks to induce acute demyelination, or for 12 weeks to induce chronic demyelination. Both conditions primarily affect the corpus callosum [96–98]. After cuprizone is removed from the diet, the toxin-induced demyelination recovers through endogenous myelin regeneration [98].

The cuprizone model has several advantages, including good reproducibility and ease of use. However, a significant drawback is that cuprizone is commonly used to evaluate the effects of drugs on axonal repair [99] and lacks the inflammatory microenvironment present in MS lesions [100]. To address these limitations, the EAE model and cuprizone model were combined into an enhanced cuprizone model [101], which introduces an inflammatory response, T-cell activation, and reactive gliosis. In this new model, myelin-reactive CD4^+^ T-cells, polarized to a Th17 profile, are adoptively transferred into mice fed with cuprizone for 4 weeks. The inflammatory T-cells migrate to the site of demyelination and inhibit the differentiation of OPCs, thereby affecting the spontaneous remyelination that typically occurs following the cessation of cuprizone treatment [101, 102].

#### Lysolecithin-induced demyelination

Lysolecithin, also known as lysophosphatidylcholine (LPC) is an endogenous lysophospholipid that increases cell membrane permeability, thus triggering Ca^2+^ influx and Ca^2+^-mediated degeneration of myelin, ultimately leading to demyelination [103]. This model is established through stereotaxic injections of 1% LPC into specific regions of the spinal cord or corpus callosum of C57BL/6 mice [104]. This procedure evokes rapid localized demyelination while preserving axonal integrity, allowing for precisely controlled timing and location of the injury [103, 105]. In contrast to the cuprizone model, which induces demyelination by influencing the metabolism of mature oligodendrocytes [98], the LPC model reveals direct toxic effects of LPC on myelin [105, 106], serving as a valuable platform for studying mechanisms of remyelination and evaluating therapeutic strategies. However, it does not replicate the immune response characteristic of MS lesions [89].

In clinical practice, over a half of patients experience varying degrees of dyskinesia related to demyelination [107]. In animal studies, both cuprizone- and LPC-induced demyelination do not result in significant motor dysfunction, whereas EAE leads to dyskinesia without recovery due to extensive lesions [108]. To address this issue, a new internal capsule local demyelination model [108] was recently established by injecting 1 μl of 1% LPC into the right posterior internal capsule of C57BL/6 mice using stereotactic techniques. This model presented acute demyelination with motor deficits at 7 days post-lesion. Notably, mice showed recovery of motor function at 14 days and complete recovery by 28 days. Immunofluorescence staining showed a large number of OPCs recruited to the lesion, differentiating into mature oligodendrocytes to form myelin sheaths along with a marked decrease in microglia and macrophages, indicating myelin regeneration and resolution of inflammation. This model provides an experimental tool for investigating the relationship between myelin regeneration and motor function recovery, as well as for developing drugs to promote these processes [109].

### Neuromyelitis optica spectrum disorders

Neuromyelitis optica and its related disorders (including unilateral optic neuritis, isolated or recurrent transverse myelitis, longitudinally extensive transverse myelitis or isolated autoimmune brain lesions) are classified as NMOSD [110]. These are rare autoimmune diseases affecting the CNS, particularly targeting the optic nerves and spinal cord. As a result, they can lead to vision loss, varying degrees of paralysis, and conditions such as area postrema syndrome, brainstem syndrome, and diencephalic syndrome. Research indicates that whole-brain atrophy in patients with NMOSD predominantly occurs in the white matter. Additionally, there is widespread demyelination and necrosis, accompanied by the loss of axons and astrocytes [111, 112].

The majority of individuals affected by NMOSD exhibit seropositivity for IgG autoantibodies that target aquaporin-4 (AQP4). As a result, most current experimental models are based on AQP4 and focus on astrocytes [113, 114]. To date, two strategies were developed for generating animal models of NMOSD [115–119]: (i) immunization against NMOSD-IgG in an EAE model [117, 120–122]; and (ii) co-injecting NMOSD-IgG with human complement into rodents, which directly mimics the pathophysiology observed in specific tissues or organs such as the optic nerve, spinal cord, and cerebellum [123–128]. These experimental models have effectively emulated several pathological manifestations characteristic of NMOSD, including loss of AQP4 expression, demyelination, astrocytic pathology, neuronal loss, infiltration of inflammatory cells, and complement activation [119].

Subcutaneous immunization with the AQP4 p201-220 peptide fragment induces NMOSD pathology in C57BL/6J mice, albeit with a low success rate [129]. However, intradermal injection of the AQP4 p201-220 peptide is much more effective, achieving a manifestation rate exceeding 80% [130]. This high success rate may be attributed to increased antigen-specific IgG production resulting from intradermal immunity, thereby providing new insights into understanding NMOSD pathogenesis [131].

Despite well-established role for AQP4-IgG antibodies in disease progression, the passive transfer of AQP4-IgG alone into animals does not reproduce the full spectrum of NMOSD features in human [132]. This discrepancy may stem from the difference of immune tolerance [133]. Consequently, an EAE model or human complement are incorporated to actively induce inflammation [132]. Notably, there are arguments that in rats with an intact complement system, the additional transfer of human complement may be unnecessary. This is because the AQP4-IgG antibody alone can elicit a pathological response, which contrasts with the need to co-transfer AQP4-IgG and human complement in mouse [119, 134]. Injection techniques also contribute to discrepancies between rodent models and humans. A new rodent model has been developed that opens the blood-brain barrier using low-frequency ultrasound, thereby enhancing the effects of low-dose NMO-IgG and complement derived from NMOSD patients when administered via tail vein injection to mice suffering from pre-existing neuroinflammation induced by EAE [135]. This methodology effectively reproduced key pathological characteristics of NMOSD, inducing an enhanced inflammatory response and demyelination specifically in the spinal cord and optic nerves.

Collectively, although immune regulations are crucial for demyelination and remyelination in NMOSD, the underlying mechanisms and the types of subset immune cells engaged may vary. For example, the pathogenesis of NMOSD in humans involves specific antibodies produced by sensitized B lymphocytes that bind complement, leading astrocyte endfeet damage and breaching the blood-brain barrier, which leads to demyelination [40, 123]. In rodent models, demyelination is also primarily mediated by immune cells, but these are macrophages, CD4 + T cells, and CD8 + T cells, rather than B cells in humans, orchestrate the demyelination process [40–42]. To better understand the NMOSD, future researches are required to deeply analyze the functional differentiation of cellular heterogeneity in immune responses, signal transduction, etc.

### Acute Disseminated Encephalomyelitis: TMEV model

ADEM is an acute inflammatory demyelinating disease that affects children, manifests rapidly, and is characterized by widespread damage of white matter in the brain and spinal cord [136]. The exact etiology of ADEM remains unclear; however, it may be linked to immune system dysregulation resulting from vaccinations and viral infections [137–139]. Given the complexity of ADEM’s pathogenesis, there are currently no well-established animal models for studying this condition [132].

In the past, the Theiler’s murine encephalomyelitis (TME) in mice was suggested to be a rodent model to simulate ADEM. After susceptible mouse strains are inoculated with TME virus (TMEV), they exhibit subacute encephalitis and significant demyelination in the CNS [140]. Ultrastructural analysis of TMEV-induced demyelinating mice revealed axonal degeneration, demyelination, and remyelination in the white matter of the spinal cord [141, 142].

### Recapitulation

Existing rodent models have advantages and disadvantages, highlighting the necessity for further refinement, especially as novel pathological characteristics and mechanisms are continually being identified. For example, it is essential to take into account the effects of overactivated Wnt pathway in OPCs regarding the initiation and advancement of the disease [94, 95] within the framework of injury when developing MS rodent models. Furthermore, non-human primate (NHP) EAE models more closely mimic the manifestations of human disease. For instance, the accumulation of iron in the brains of rhesus monkey EAE models is similar to that found in human brains, which may correlate with elevated levels of oxidative stress [143]. Conversely, iron accumulation in the brains of rodents is relatively low, and there is a recognized inadequacy in replicating oxidative stress and injury in rodent EAE models [144]. As a result, NHP models are recently considered [132].

## Rodent models of neurodegenerative diseases

Impaired integrity of white matter represents a fundamental pathological feature of age-dependent neurodegenerative disorders. Aging is associated with significant reduction in the number of oligodendroglia, particularly in the white matter [145]. Magnetic resonance imaging (MRI) and autopsy indicate a significant reduction in white matter volume in AD [146]. Additionally, both AD and PD patients exhibit compromised myelination and impaired repair mechanisms [147]. Although the primary pathological hallmark of PD is the degeneration of dopaminergic neurons in the substantia nigra, disruption in myelin is also detected [148].

### Alzheimer’s disease

AD is characterized by formation of β-amyloid plaques and intraneuronal neurofibrillary tangles composed of hyperphosphorylated tau protein [149–151]. Recent studies demonstrated abnormal oligodendroglia and myelin in the brains of patients with AD [152–155], suggesting that myelin degeneration may be a key pathophysiological aspect of AD, potentially occurring before the onset of amyloid pathology and cognitive decline [156, 157]. Defects in myelin contribute directly and indirectly to the formation of β-amyloid plaques [158]. Specifically, the deletion of BACE1 (β-site APP-cleaving enzyme 1) from oligodendroglia using an Olig2-Cre mouse line, significantly reduced β-amyloid plaque formation in a mouse model of AD, highlighting the contribution of oligodendroglia to Aβ plaque development [159]. Therefore, enhancing the integrity of myelin and oligodendroglia could represent a promising therapeutic target for AD.

Murine models of AD are mainly based on over-expression of familial AD-related genes, including amyloid precursor protein (APP) and presenilin-1 (PS-1), to reproduce β-amyloid plaque formation, alongside with misohosphorylated tau protein to mimic neurofibrillary tangles [160]. Usually, transgenic mice carrying multiple familial Alzheimer’s disease (FAD) mutations, such as APPswe/PSEN1ΔE9 (APP/PS1), 5xFAD and in triple-transgenic (3xTg), exhibit more significant Aβ deposition and progression of behavioral deficits compared to single APP transgenic mice [161–163].

#### APP/PS1 model

At 2 months of mouse age, APP/PS1 mice exhibit substantial alterations in myelin morphology and oligodendroglial development [163]. By 6 months, these mice show cognitive deficits alongside corpus callosum shrinkage and impaired maturation of oligodendrocytes [164]. In the hippocampus of 9 months old APP/PS1 mouse, a decrease in the density of OPCs was detected. At 14 months, these OPCs exhibit a shrunken and fibrous morphology, which highlights the relevance of OPC disruption as a pathological signature in the AD [165].

#### 3xTg model

The 3xTg AD mouse model, which incorporates the mutated tau protein gene in addition to APP/PS1 mutations, replicates key histopathological features of AD [160], including both β-amyloid plaques and tangles [43, 166, 167]. In this model, myelin loss accelerates with age, and OPCs exhibit significant disruption at six months during disease progression, marked by striking morphological atrophy and a clear decrease in self-renewal capacity [168].

#### 5xFAD model

The 5xFAD mouse model is recognized for its rapid progression of disease, displaying substantial pathological characteristics at an early stage. These mice begin to express intracellular β-amyloid at 1.5 months, followed by extracellular β-amyloid deposition, formation of senile plaques, and neuronal deficits by 2 months. Cognitive decline occurs significantly earlier (4 to 6 months) compared to other AD models, making it a valuable tool for investigating early disease pathogenesis [169, 170]. In 5xFAD mice, significant ultrastructural disorganization of myelin in hippocampal nerve fibers emerges with age, mirroring observations seen in human AD. Specifically, this deterioration of myelin integrity in 5xFAD mice (from 6 months onward) is localized in the CA1 region of the hippocampus, rather than a general loss of myelin structural proteins or thinning of myelin sheaths [171].

Several newer AD models utilize knock-in technology to integrate humanized β-amyloid sequences and mutations associated with FAD into the endogenous *App* locus of murine models. These models more accurately reflect the pathological characteristics of human AD, reducing the artifacts often seen in overexpression models [172]. The observations in these models showed that changes in oligodendrocytes preceded the structural changes in white matter, implying that interruption of oligodendrocyte homeostasis may be a trigger to white matter alternation in the pathological progression of AD [173].

### Parkinson’s disease

PD is a chronic neurodegenerative disorder, characterized by the progressive loss of dopaminergic neurons in the substantia nigra compacta of the midbrain, along with the abnormal accumulation of α-synuclein in the form of Lewy bodies [174–178]. Although traditionally regarded as a grey matter disorder, significant alterations in white matter in individuals with PD was detected, showing oligodendroglial malfunction, with a predilection for demyelination in the periventricular and deep white matter, as well as disconnections in white matter tracts [179]. These changes can exacerbate both motor and non-motor symptoms and may contribute to the progression of PD, independent of the loss of dopaminergic neurons in the substantia nigra [180]. SnRNA-seq analysis of post-mortem brain tissue from prefrontal cortex and anterior cingulate regions have further revealed a strong association between oligodendrocytes and OPCs with risk loci linked to PD [181].

Most of PD models are based on either neurotoxin exposure or genetic modification [174]. Injection of neurotoxin such as 1-Methyl-4-phenyl-1,2,3,6-tetrahydropyridin (MPTP) and 6-hydroxy-dopamine (6-OHDA) induces PD-like pathology by triggering oxidative stress and promoting the degeneration and death of dopaminergic neurons.

#### MPTP model

The MPTP (a lipid-soluble compound capable of crossing the blood-brain barrier) is commonly administered by intraperitoneal injection in C57BL/6 mice. This model reproduces several core pathological features of Parkinson’s disease [182]. The MPTP-induced oxidative stress [183] may directly damage oligodendrocyte mitochondria, resulting in disordered and discrete myelin structure in the spinal cord [184]. Due to its simplicity and reproducibility, this model is widely used in Parkinson’s disease studies [185, 186]. In the MPTP-induced Parkinson’s disease mouse model, a decrease in oligodendrocytes in the striatum was observed at days 3 and 7 following injection [187].

#### 6-OHDA model

The 6-OHDA cannot cross the blood-brain barrier hence stereotactic injection into relevant areas of the substantia nigrostriatal pathway, including the substantia nigra pars compacta (SNpc), striatum, and mediodorsal forebrain bundle is required. This induces acute degeneration of dopaminergic neurons [188, 189]. Injection of 6-OHDA administration leads to complete degeneration of nigral dopaminergic neurons. Although no remarkable changes in the ratio of the various glial types and in the myelin structures in 6-OHDA model were detected an insufficient maturation of oligodendrocytes resulted in a 5-days delay in the onset of myelinogenesis, suggesting that the myelin degeneration in the mesencephalic tract specifically impaired by 6-OHDA [190]. Anti-inflammatory therapy can mobilize adult neural stem cells, which subsequently differentiate into oligodendrocytes within the striatum. These newly generated oligodendrocytes contribute to enhancing axonal stability and functionality in this model [191]. However, the 6-OHDA model diverges from the chronic progressive pathology of human Parkinson’s disease, as it predominantly elicits acute effects - most notably the rapid degeneration of dopaminergic neurons [174, 192]. In contrast, human PD pathogenesis involves progressive α-synuclein accumulation in oligodendrocytes, leading to chronic myelin defects, a feature absent in the 6-OHDA model. This discrepancy limits the model’s utility for studying glia-driven neurodegeneration [178].

#### Transgenic PD model

Transgenic models are instrumental to characterize the impact of specific genetic variants on PD pathogenesis, including the overexpression of α-synuclein and *Leucine-rich repeat serine/threonine kinase 2* (*LRRK2*), and the knockout/knockdown of genes like *Parkin, Dj-1*, *PTEN-induced putative kinase 1* (*Pink1*) [193]. Among these variants, LRRK2 mutations (e.g., G2019S) may impair oligodendroglia maturation by disrupting mitochondrial dynamics [194] and development, thereby exacerbating axonal demyelination in the corpus callosum [195–197]. *SNCA* is the first one linked to familial PD, with the protein it encodes, α-synuclein, being a key component of Lewy bodies [197–199]. The A53T-mutated form of *SNCA* (A53T-*SNCA*) is a well-studied familial mutation in PD [198, 200]. In this mouse model, α- synuclein is overexpressed in neurons, and hypomyelination of axons, along with age-dependent loss of MBP localized to the striatum, is correlated with the progression of α-synuclein pathology [201]. Furthermore, in the PLP-α-synuclein mouse model, oligodendroglial overexpression of human α-synuclein induces progressive degeneration of specific brain regions, including the substantia nigra pars compacta (SNpc), locus coeruleus, nucleus ambiguus, pedunculopontine tegmental nucleus, laterodorsal tegmental nucleus, and Onuf’s nucleus [202, 203]. In contrast, neurodegeneration in the MBP-α-synuclein mouse model correlates with dysregulation of oligodendrocyte-derived neurotrophic factors particularly with reduced glial cell line-derived neurotrophic factor (GDNF) [204]. These findings highlight distinct pathogenic mechanisms underlying oligodendroglial α-synucleinopathy and suggest novel insights into its contribution to neurodegeneration [205].

### Amyotrophic lateral sclerosis: SOD1 model

Amyotrophic lateral sclerosis (ALS) is characterized by the progressive degeneration of motor neurons in the spinal cord, brainstem, and motor cortex. About 10–15% of ALS cases are familial, typically inheriting dominant traits [14, 206]. Pathological changes in white matter regions may occur before the structural changes in motor neurons become apparent [207, 208]. Progressive microstructural alterations in white matter, as measured by diffusion tensor imaging (DTI), are particularly evident in the corticospinal tract and corpus callosum in ALS patients [209–211]. Additionally, abnormalities in myelin sheaths and the aggregation of pathological proteins within oligodendrocytes suggest the involvement of these cells in the pathogenesis of ALS [212, 213].

Since the identification of mutations in Cu, Zn superoxide dismutase-1 (*Sod1*) [214], over 50 genes were linked with ALS [215, 216]. Most of current ALS mouse models are transgenic, created by random insertion of human mutant ALS genes. *Sod1* (encoding superoxide dismutase) [215], *C9orf72* (encoding guanine nucleotide exchange factor) [217], *Fus* (encoding RNA-binding protein FUS) [218], and *Tardbp* (encoding TAR DNA-binding protein 43, TDP43) [219], are the most extensively studied mutations [220]. Conditional knockout mouse model in which *Fus* gene is selectively depleted in oligodendrocytes demonstrated an increase in myelination and the ensheathment of axons [221]. Furthermore, selective deleting of TDP-43 in mature oligodendrocytes can lead to progressive myelin defects in both the gray and white matter of the brain and spinal cord [222].

With the identification of multiple ALS-associated variants, several *Sod1* transgenic models were developed, including the G93A, D83G, D85G, D86G, D90A, and G37R variants [220]. Among these, SOD1^G93A^ mouse strain was the first created to model ALS [223]. Although representing only 15-20% of point-specific mutations in familial ALS cases [224], this model has become a significant focus of research. In the white matter of the ventral lumbar spinal cord in SOD1^G93A^ mice at postnatal day 30 (P30), researchers observed a mild yet statistically significant decrease in the number of mature oligodendrocytes, with this trend worsening as the disease progressed to the late stage at P120. There was also a substantial reduction in the number of newly matured CC1^+^ oligodendrocytes within the ventral spinal cord white matter, suggesting potential impairments in differentiation during the early developmental stages of the spinal cord in SOD1^G93A^ mice [225].

### Recapitulation

To date, no animal model fully replicates all the pathological hallmarks of AD and PD, there are notable differences in myelination patterns observed in humans compared to those in animal models. For instance, postmortem examinations of human AD patients reveal a higher rate of abnormal myelination in the alveus, while the perforant path in AD mice shows a lower rate of myelin abnormalities [163, 171]. In the context of PD, models based on neurotoxin exposure typically recreate specific lesion sites; however, most genetic modification models have been unsuccessful in mimicking the loss of dopaminergic neurons [226]. Furthermore, variations among species can lead to distinct characteristics in these models. Therefore, new approaches should be considered to address the gaps that exist between the pathogenesis of these models and the actual diseases.

## Rodent models of vascular damage-related disorders

Vascular damage-related disorders and white matter injury are closely interconnected through shared mechanisms involving impaired hypoxia-ischemia, restricted blood flow, and endothelial malfunction. In clinical and related animal studies, acute stroke or chronic hypoxia directly disrupt the blood-brain barrier, allowing toxins and inflammatory mediators to infiltrate white matter, triggering oligodendroglial damage and death resulting in demyelination. White matter damage resulting from vascular causes clinically manifests ins gait disturbances, cognitive impairment, and executive deficits [15, 227].

### Ischemic stroke

Stroke is a leading cerebrovascular disease; approximately 80% - 85% of strokes are ischemic, and the rest are hemorrhagic. Compared with gray matter, white matter has limited collateral circulation, making it highly susceptible to ischemic injury; therefore, white matter damage often accounts for half of the lesion volume in patients with ischemic stroke [228]. Additionally, there is a positive correlation between the severity of white matter damage and neurological deficits, leading to demyelination and axonal degeneration [229–231].

#### Middle cerebral artery occlusion model

In humans the middle cerebral artery is a common site for occlusion. Consequently, the middle cerebral artery occlusion (MCAO) model is widely accepted as the standard animal model for studying focal permanent cerebral ischemia [232]. This model typically results in severe nerve fiber disorders, vacuolization, and loss of myelin fibers during the early stages, with little to no significant recovery [233].

In recent years, the transient middle cerebral artery occlusion (tMCAO) model, created using a thread occlusion method, gained popularity for investigating neurological recovery following treatments [234, 235]. In this model, a suture is inserted through the external carotid artery to temporarily block the middle cerebral artery, which is then removed to restore blood flow. Usually, male Sprague-Dawley (SD) rats [236, 237] and C57BL/6 J mice [234] are used for tMCAO modeling. Arguably, the BALB/c mouse strain, which has a limited ability to compensate for collateral circulation, can be used to establish a no-reflow model, showing more severe white matter damage compared to C57BL/6J tMCAO, while exhibiting similar damage to that observed in the permanent MCAO model [238]. In tMCAO, the myelin sheaths suffer extensive destruction. The most severe white matter damage occurs 7 days after ischemic stroke, followed by a gradual recovery that continues from day 7 to day 30 [234]. Although the density of OPCs in the lesion area doubles after 8 weeks, their differentiation is inhibited, leading to slow remyelination. Furthermore, the capacity for myelin regeneration significantly declines with aging, resulting in virtually no new myelin sheaths in the brains of aged rats that experience a stroke [239].

#### Bilateral common carotid artery occlusion model

Bilateral common carotid artery occlusion (BCCAO) in rats [240–242] and bilateral common carotid artery stenosis (BCAS) in mice [243–245], model global cerebral ischemia, allowing for examination of vascular cognitive deficits and white matter damage resulting from chronic cerebral hypoperfusion. The BCAS model gained considerable acceptance due to its reproducibility; a refined version of this model has been developed that employs 0.18 mm microcoils to induce bilateral common carotid artery stenosis in C57BL/6J mice. This enhanced model effectively reproduces cognitive impairments and white matter lesions akin to those observed in human vascular cognitive impairment over a four-week postoperative duration [246]. Notably, it is characterized by compromised myelin integrity and a significant increase in the populations of microglia and astrocytes, particularly in the medial region of the corpus callosum adjacent to the lateral ventricle [244]. Furthermore, demyelination within the corpus callosum of the model mice can persist for up to 8 weeks and is found to be more pronounced than axonal loss [247]. To investigate the ischemic stroke associated with tauopathy, the BCCAO surgery to mimic chronic cerebral hypoperfusion, is combined with subsequent MCAO surgery in rats to mimic acute ischemic stroke. In this model, microtubules in myelin sheath showed partial colocalization with the hyperphosphorylated tau, whereas oligodendrocytes contain high levels of tau [248].

#### Endothelin-1 induced model

Endothelin-1 (ET-1) is a powerful vasoconstrictor peptide associated with potential damage to brain cells. Elevated levels of ET-1 in stroke patients who have suffered a stroke correlate with a poor prognosis [249]. When ET-1 is administered into the internal capsule using stereotaxic techniques, it leads to lacunar cerebral infarction in SD rats [250, 251]. This injury is confined to the white matter and is marked by considerable demyelination, while the axonal structures remain preserved [252]. Additionally, there is a significant increase in the proliferation of OPCs in the demyelinated regions 7 days post-injection [253]. Adapting this model for use in mice presents challenges due to the higher expression of vasodilatatory endothelin_B_ receptors in murine brains, which are associated with nitric oxide production that counteracts vasoconstriction. Nevertheless, the co-administration of ET-1 and L-NAME, a nitric oxide synthase inhibitor, successfully induced focal cortical ischemia in the sensorimotor cortex of C57BL/6 mice, resulting in substantial infarcts and enduring sensorimotor deficits, thus offering an alternative method for stroke research [254].

### Intracerebral Hemorrhage

Intracerebral hemorrhage (ICH), while less prevalent than ischemic stroke [255], presents a significantly higher mortality rate, estimated at 30–40% during the acute phase [256, 257]. The white matter damage caused by ICH can be divided into two types: primary and secondary. Primary white matter injury occurs due to the compression of adjacent brain tissue by the hematoma or perihematomal edema, leading to mechanical deformation of nerve fibers. In contrast, secondary white matter injury arises from the detrimental effects of various metabolites found within the hematoma, which inflict both direct and indirect harm to white matter through neuroinflammatory processes and oxidative stress [258, 259]. These pathological changes include demyelination, axonal damage, and the degeneration of oligodendrocytes in specific focal regions, with a notable prevalence in the basal ganglia [260]. Currently, autologous blood injection and collagenase injection are widely utilized methods for inducing ICH, with spontaneous cerebral hemorrhage serving as a more optimal animal model.

#### Autologous blood injection model

The autologous blood infusion technique entails the injection of non-heparinized autologous arterial blood into specific brain regions to promote hematoma development [261, 262]. For example, a mouse model of ICH was created by administering autologous blood into the lateral ventricle [263]. In comparison to the sham-operated group, significant edema and demyelination were observed in the bilateral corpus callosum following the induced hemorrhage. Additionally, there was a significant reduction in the number of oligodendroglial lineage cells in the ipsilateral periventricular area as the disease progressed, with the most pronounced decrease occurring three days post-injection [263]. In the mouse basal ganglia ICH model, oligodendrocytes in the ipsilateral basal ganglia region experienced extensive apoptosis within 24 hours of injection, leading to severe demyelination, which was confirmed by transmission electron microscopy revealing a thin and fragile myelin layer [264]. By increasing the times of injections, the model’s stability and reproducibility can be significantly enhanced [261, 265, 266]. Of note, this model exhibits a time-dependent recovery from neurological deficits [267], which may restrict long-term studies; however, it remains a valuable tool for investigating the brain’s responses to blood exposure, including mild inflammatory reactions, apoptotic processes, and mechanisms of blood clearance [268].

#### Collagenase injection model

The collagenase injection model employs collagenase to degrade the extracellular matrix surrounding capillaries, resulting in hemorrhage [269, 270]. Collagenase type IV is predominantly used due to its high efficacy. This model shows myelin damage characterized by progressive swelling and fragmentation of myelin bundles. This model has a propensity for spontaneous recovery, as indicated by a significant rise in both OPCs and mature oligodendrocytes in the vicinity of the hematoma within the first seven days. These cells were primarily concentrated in white matter tracts, resulting in myelinated axon bundles that were denser, more swollen, and less fragmented after seven days [271]. When compared to the autologous blood infusion model, the collagenase injection model effectively replicates the essential pathological changes associated with ICH. However, the collagenase method more accurately reflects the clinical stages of hemorrhagic stroke, including vascular rupture, hematoma formation, and hematoma expansion, thus providing a more realistic experimental setting for research [272, 273]. Nevertheless, there are concerns regarding the potential for increased animal mortality due to excessive administration of collagenase [274].

#### Spontaneous cerebral hemorrhage model

The model for spontaneous cerebral hemorrhage employs the spontaneous hypertension stroke-prone (SHRsp) rats, with a stroke incidence of up to 80%. In SHRsp rats, alterations in white matter are often observed at 4 to 5 months of age, occurring prior to the infarction and hemorrhage that manifest at 9 months [275]. These pathological features closely mimic those found in human small vessel disease characterized by varying degrees of cerebral hemorrhage, abnormal dilatation of small blood vessels, softening of brain tissue, and disorganized or absent alignment of nerve fibers [276]. Additionally, white matter hypoxia activates matrix metalloproteinases (MMP), which can compromise the integrity of the blood-brain barrier, resulting in vasogenic edema and damage to deep white matter myelinated nerve fibers, leading to non-immune demyelination [277, 278]. However, the high costs and maintenance challenges associated with this model limit its widespread use; furthermore, a diet rich in salt and fat, combined with low protein intake, can accelerate lesion development and reduce the lifespan of these rats, thereby constraining the duration of experiments [279–281].

### White Matter Injury in Preterm Infants

Although the survival rate of preterm infants has markedly improved over the years, the neurological and neuropsychiatric outcomes, especially in relation to white matter injury (WMI), remain a problem [282]. In preterm infants, white matter injury primarily manifests as periventricular WMI, particularly affecting the areas adjacent to the lateral ventricles and centra semiovalia [283]. This condition is pathologically defined by a continuous loss of myelin and subsequent disturbances in white matter maturation [284].

#### White matter injury model

The first white matter injury model (known as Vanucci model) was established in 7-day-old Sprague-Dawley rats. This model incorporated unilateral ligation of the common carotid artery alongside a 3.5-hour exposure to 8% oxygen hypoxia. Consequently, a 90% injury rate was achieved’ the model was characterized by moderate to severe cystic necrosis in the cerebral cortex, striatum, and hippocampus [285]. In this model, the rat brains are thought to be similar to that of human fetuses at 32 to 34 weeks of gestation, marked by rapid myelination in the white matter [286]. Thus, the white matter damage is primarily localized in dorsal foci and extends laterally along the corpus callosum, with significant myelinogenic activity occurring in the affected regions [285]. For instance, between 15- and 26-hours post-injury, a significant reduction in myelin structures, exposure of axons, and disorganized nerve fiber arrangement was observed. By 36 to 50 hours, there was an increase reactive astrocytes and microglia and infiltration of macrophages, along with a considerable number of mitotic divisions [285]. However, factors such as the body weight of the rats, duration of anesthesia, and site of ligation can influence the severity of the injury, resulting in variability and diminished reproducibility of this rat model [287].

The most widely used refinement involves unilateral common carotid artery ligation followed by a 10-minute hypoxic exposure (6% oxygen) in P3 rats. This model effectively reproduces injuries observed in preterm infants at 24 to 28 weeks of gestation, demonstrating less white matter damage compared to the Vannucci model [288]. The damage is primarily restricted to the corpus callosum, where structural changes are most evident at P14, revealing an irregular arrangement of nerve fibers, a reduction in callosal volume, vacuolization, atrophy, a loss of oligodendrocytes, and hypomyelination [289, 290].

#### Diffuse white matter injury model

Recent developments in obstetric and neonatal care significantly altered the characteristics of white matter injury in preterm infants, shifting from cystic necrosis to a more complex form of diffuse gliosis, which is characterized by an ongoing myelin degradation [291]. This type of injury primarily results from the impacts of hypoxia and inflammation on O4+ premyelinating oligodendrocytes, which represent a crucial stage of the oligodendroglial lineage during the gestational period of 24 to 32 weeks [291]. To investigate the connection between systemic inflammation and myelin damage, a mouse model of diffuse white matter injury (DWMI) was developed by inducing an inflammatory response through intraperitoneal administration of interleukin-1β (IL-1β) which was given twice daily from postnatal days P1 to P4 and once in the morning on day P5 [292, 293]. Inflammation induced by IL-1β disrupts the expression of essential transcription factors that regulate oligodendrocyte maturation (such as OLIG1, SOX10, NKX2.2), leading to a reduction in the population of mature oligodendrocytes and an increase in population of oligodendrocyte precursor cells (OPCs). This imbalance results in diminished myelination, as evidenced by a higher ratio of unmyelinated axons and a reduction in the diameter of myelinated axons [294]. As a result, white matter injury occurs diffusely across various regions, including the corpus callosum, cingulate gyrus, external capsule, basal ganglia, and cerebral cortex [294]. This model provides valuable insights into the interplay between systemic inflammation, myelin development, and white matter damage.

### Recapitulation

Overall, the existing models are capable of reproducing the occurrence as well as the pathological characteristics and mechanisms associated with white matter injuries in ischemic stroke, ICH and WMI of preterm infants. However, the specific mechanisms may differ from those observed in the actual diseases. Future efforts in the development and refinement of models need to concentrate on enhancing the replication of the precise location, severity, and progression of disease manifestation in human patients.

## Rodent models of traumatic and infectious diseases

White matter injury accompanies both traumatic injury and infectious diseases, as both disrupt myelin health. For instance, traumatic brain injury leads to white matter hyperintensities, followed by secondary injuries such as ischemia, excitotoxicity, and neuroinflammation, all of which exacerbate white matter damage [295]. Infections caused by bacteria or viruses or also induce white matter lesions [296], ultimately resulting in leukoencephalopathy, motor dysfunction, cognitive deficits, and neuropsychiatric symptoms.

### Traumatic brain injury

Traumatic brain injury (TBI) is an acquired acute brain trauma resulting from external mechanical forces which is among the primary causes of death and disability globally [297]. The development of TBI involves both primary and secondary injury mechanisms. Primary injury refers to the immediate localized damage to the brain tissue, which may include intracranial hematomas, skull fractures, lacerations, contusions, and penetrating injuries [298]. Secondary injury triggers a series of metabolic, cellular, and molecular processes that occur following the primary injury, ultimately leading to neuronal death, tissue damage, and atrophy [295]. White matter damage is a major component of TBI. White matter atrophies can persist in the corpus callosum for more than 20 months after trauma. Four models are widely used to study secondary injury after TBI: the controlled cortical impact injury (CCI), weight drop impact acceleration injury, the fluid percussion injury (FPI) and blast-induced injury model [295].

#### CCI model

The CCI model is a well-established open brain injury model that utilizes a pneumatic or electromagnetic device to deliver an impact to the exposed, intact dura mater [299–302]. This model provides precise control over various parameters such as impact location, depth, velocity, and duration, facilitating the simulation of TBIs with varying degrees of severity [303]. Additionally, the impact head can be swiftly retracted following the impact, thereby reducing the risk of rebound injury [295]. Notable pathological changes, including demyelination and reduction in myelinated axons within the corpus callosum, were documented 3 days after TBI [304].

#### Weight drop model

The weight drop impact acceleration injury model includes Feeney’s weight drop model, Shohami’s weight drop model, Marmarou weight drop model and Maryland’s weight drop model. Feeney’s model applies weight directly onto the intact dura mater exposed through craniotomy, causing cortical contusion and white matter hemorrhage, with oligodendrocyte death and focal myelin loss observed in perilesional regions [305]. The Shohami’s model represents a closed head injury, where focal blunt force is applied to the unprotected skull, resulting in reactive gliosis and acute oligodendrocyte apoptosis, accompanied by myelin degradation [306]. The Marmarou model designed to reproduce diffuse TBI, typically produces widespread axonal injury in myelinated tracts (corpus callosum, brainstem), marked by oligodendrocyte necrosis, and demyelination [307]. This model is characterized by widespread bilateral damage affecting neurons, axons, dendrites, and microvasculature, with significant axonal injury particularly noted in the corpus callosum, internal capsule, optic tract, and long tracts within the brain stem [308]. Lastly, Maryland’s weight drop model, which builds upon the Marmarou framework, focuses on the impact force directed at the forehead to induce TBI through anterior-posterior and sagittal rotational acceleration of the brain within the intact skull. This model simulates the frontal impacts often encountered in motor vehicle and sports-related accidents, characterized by the absence of cortical contusion, skull fracture, prolonged apnea, and low mortality rates, while still demonstrating axonal injury [309].

#### Fluid percussion injury model

FPI causes damage by rapidly injecting fluid into the epidural space. In rodent studies, this is primarily achieved through a lateral fluid percussion model (LFPI). The LFPI method results in localized cortical contusions and widespread subcortical neuronal injury, predominantly affecting the thalamus, the ipsilateral hippocampus, the septum pellucida, the septum striatum, and the amygdala [310, 311]. Mature oligodendrocytes are specifically vulnerable to this injury model [312]. In the focal but not distal corpus callosum, FPI caused a decrease in CC1^+^ as well as BCAS1^+^ actively myelinating oligodendrocytes and reduced intensity off FluoroMyelin staining. Disruption in node-paranode organization and loss of Na_v_1.6^+^ were observed even in areas without obvious axonal damage [312]. The number of apoptotic oligodendrocytes, and degeneration of myelinated tracts are frequently observed post-FPI; recent studies highlight a concomitant increase in the number of OPCs from day 2 (Olig2^+^) to day 7 (Tcf4^+^) post-injury [313].

#### Blast-induced model

Non-impact blast-induced neurotrauma (BINT) refers to diffuse axonal injury caused by the detonation of compressed air or explosives. This model is characterized by diffuse cerebral edema, extreme hyperemia, and delayed vasospasm [314]. In particular, axonal injury was the most notable characteristic observed in the initial two weeks following blast exposure in rats that were provided with body shielding [315]. Oligodendroglial marker 2’,3’-cyclic nucleotide 3’-phosphodiesterase, as well as vascular endothelial growth factor, both displayed delayed (14-28 days) and pressure-dependent responses [316].

### Traumatic spinal cord injury

Traumatic spinal cord injury (SCI) represents a serious medical challenge, leading to profound impairments in motor, sensory, and autonomic functions. Demyelination caused by secondary Wallerian degeneration or retrograde degeneration after SCI [317]. This demyelination is driven by oligodendroglial death by necrosis and apoptosis [318], triggered by ischemia, excitotoxicity, free radical production, and immune cell infiltration [319]. In parallel, a rapid and protracted OPCs proliferative response occurs, especially at the lesion borders, albeit posttraumatic endogenous remyelination is rarely complete [320, 321]. OPCs also contribute to perilesional border formation and restrict the regeneration of injured axons after SCI alter gene expressions of cytokines and perpetuate the immune response [321]. Without timely intervention, it may progress to a chronic secondary injury characterized by the development of cystic cavities, axonal dieback, and the maturation of glial scars [322]. Current treatments for SCI encompass pharmacological interventions, neuronal implantation, and stem cell therapies, all designed to enhance axonal regeneration, facilitate myelin repair, manage neuroinflammation, and reduce lesion cavities. However, these approaches provide only temporary benefits and do not effectively prevent chronic complications [322].

Injury models of the spinal cord are categorized into several types: contusion, compression, dislocation, distraction, transection, and chemical lesion [323]. Rats are most frequently used due to low cost, ease of handling, and low surgical infection risk [324]. Although the cervical segments are the most common site of SCI in clinical practice, most animal models are based on thoracic spinal injuries. This preference arises from the fact that the dysfunction resulting from reduced gray matter in the thoracic spinal cord is less pronounced than that seen in cervical injuries, allowing for a focused analysis of white matter damage. Additionally, thoracic spinal injuries tend to exhibit higher survival rates in animal studies, making them both reliable and reproducible. Nonetheless, the anatomical distinctions between the cervical and thoracic regions pose significant challenges for translating experimental findings to clinical settings [324].

The contusion model is the most clinically relevant [325]. Various devices can produce this type of injury, including the weight drop method, electromagnetic strikers, and air gun projectiles. The weight drop method, initially introduced by Alfred Allen in 1911, allows for the simulation of SCI by controlling the impact location and varying the mass and drop height of the hammer [326], resembling abnormal swelling of myelin, the presence of myelin debris, reduced mean myelin thickness, and reactive astrogliosis [327–329]. However, if the heavy hammer is not removed promptly, it may lead to prolonged compression and secondary injury. Additionally, any deflection or movement of the spinal cord can create discrepancies between the expected and actual areas and severity of injury. To mitigate these issues, the traditional method has been enhanced through the incorporation of electromagnetic strikers [330] or air gun strikers [331]. Recently, electromagnetic strikers have gained popularity for detailed investigations by allowing adjustments to parameter settings [332].

### Progressive Multifocal Leukoencephalopathy: JCPyV model

Progressive multifocal leukoencephalopathy (PML) is caused by human polyomavirus 2 (also known as John Cunningham virus, or JCPyV). In individuals with severe immunosuppression, JCPyV can penetrate the blood-brain barrier, infecting and replicating within oligodendrocytes ultimately resulting in demyelination, neurodegeneration, and mortality [296]. A notable challenge in studying JCPyV is its inability to replicate in non-human cells, attributed to the absence of a species-specific factor, likely a component of the JCPyV DNA polymerase [333]. This limitation hinders the creation of animal models that can effectively mimic PML.

Previous attempts using neonatal syrian golden hamsters [334], owl monkeys [335], squirrel monkeys [336], transgenic mice [337] showed tumor formation but lacked myelin loss. In 2014, a novel mouse model was established by transplanting bipotential glial progenitor cells (GPCs) derived from fetal human brain tissue into newborn immunodeficient and myelin-deficient homozygous shiverer mice (*Rag2* ^*-/-*^ *MBP* ^*shi/shi*^). As the mice grew, the fetal GPCs migrated throughout the white matter, differentiating into oligodendrocytes and astrocytes, which resulted in significant myelination, particularly of the fully humanized myelin sheath [338, 339]. Following intracerebral injection of JCPyV, this virus infects human-derived astrocytes and oligodendrocytes. After 4 weeks, focal areas of demyelination and infection-associated astrogliosis were seen within the forebrain white matter, particularly in discrete foci abutting the callosal and fimbrial walls of the lateral ventricle. Twelve weeks later, extensive demyelination was observed [339]. However, in this model, JCPyV predominantly infects astrocytes, with oligodendrocyte apoptosis and demyelination occurring as secondary effects of astrocyte infection, which contrasts with the intense productive infection of oligodendrocytes seen in clinical PML [340]. Mouse polyomavirus (MuPyV), which shares characteristics with JCPyV, was also studied. After intracranial inoculation into C57BL/6 mice, demyelination and vacuolation of the cingulate tract and external capsule appear on day 9, with progressive ventricular enlargement and white matter edema observed on day 30 [341]. This model has advanced the understanding of JCPyV CNS invasion, but it does not fully replicate JCPyV [342].

Recently, a novel brain organoid model derived from human cells has been established, facilitating the efficient identification of JCPyV infection [343]. Immunocytochemical confocal imaging visualized viral inclusion bodies located within the enlarged, rounded nuclei of oligodendrocytes, while some were also detected in astrocytes. This model enables the visual monitoring of viral replication and sustains productive JCPyV infection for a duration of up to 3 weeks, thereby providing new insights into JCPyV research and potential therapeutic approaches [343].

### Recapitulation

The key TBI model, CCI, is suitable for investigating the biomechanics of traumatic brain injury due to its consistency and reliability. Other models may serve as alternative options for various research purposes. The SCI models, contusion, compression, transection, prioritize thoracic segments in animals due to higher survival rates, despite clinical prevalence of cervical injuries. PML, driven by JCPyV, lacks robust animal models due to species-specific replication barriers. While these models enhance mechanistic understanding, clinical applicability requires optimization to address pathological discrepancies and species limitations.

## Rodent models of genetic diseases

Gene mutation-related disorders arise from myelin malfunction, metabolic errors, or vascular defects, and are a major cause of myelin deficiency or white matter degeneration. These disorders lead to symmetric white matter abnormalities, which can manifest as motor deficits, cognitive decline, seizures, and early-onset neurological regression. For instance, leukodystrophies [344], such as X-linked adrenoleukodystrophy (X-ALD), involve progressive white matter degeneration caused by lysosomal or enzyme defects [345]. Alexander disease, associated with glial fibrillary acidic protein (GFAP) sporadic mutations, is characterized by the accumulation of Rosenthal fibers in astrocytes, resulting in the destruction of white matter [346]. Additionally, hereditary small vessel disease, exemplified by Notch3 mutations in cerebral autosomal arteriopathy with subcortical infarcts and leukoencephalopathy (CADASIL), leads to ischemic white matter lesions due to microvascular dysfunction [347].

### X-linked Adrenoleukodystrophy: Abcd1-knockout model

X-ALD is the most prevalent inherited disorder associated with aberrant *Abcd1* gene located in the X chromosome and affecting peroxisomes [348]. The core defect in X-ALD is attributed to the malfunction of the ALD protein, which leads to compromised transport and β-oxidation of very long-chain fatty acids (VLCFAs). This malfunction results in the accumulation of VLCFAs within the white matter and adrenal cortex, ultimately causing demyelination of the CNS and hypoplasia of the adrenal cortex [345]. Variations in the function of the *Abcd1* gene can produce a range of phenotypes, from asymptomatic individuals to those experiencing adrenal insufficiency and adrenomyeloneuropathy [349]. Consequently, the knockdown of the *Abcd1* gene is the predominant approach to establish experimental models of X-ALD [350–352].

*Abcd1*-deficient mice show accumulation of VLCFAs similar to that observed in human patients, however, they did not exhibit myelin abnormalities in the brain [353, 354]. The use of the cuprizone model for studying X-ALD in mice was proposed [355]. The diet of 10-week-old *Abcd1* knockout mice was supplemented with cuprizone for 5 weeks, with the aim of inducing acute brain demyelination. The results indicated that *Abcd1*-deficient mice displayed a more severe demyelination response in the context of demyelination injury. In the early stages of demyelination, *Abcd1*-deficient mice showed a more pronounced loss of mature oligodendrocytes and a higher degree of acute axonal injury compared to wild-type mice. However, the absence of *Abcd1* did not inhibit the proliferation and differentiation capabilities of OPCs in response to cuprizone-induced demyelination [355]. This finding contrasts with the previous hypothesis suggesting that abnormal epigenetic regulation in X-ALD impedes the regeneration capabilities of oligodendroglia [356]. This difference may arise from the possibility that X-ALD exhibits a more complex pathological environment compared to cuprizone-induced demyelination, or that the metabolic effects of the genetic deficiency do not escalate to a degree that disrupts oligodendroglial function [355].

The cerebral ALD (cALD) is the most severe form of cerebral inflammatory demyelination associated with X-ALD, characterized by a rapidly advancing confluent inflammatory demyelination within the brain, typically beginning in the corpus callosum, spreading through both cerebral hemispheres, and associated with extensive oligodendroglial death [355, 357]. A mouse model for cALD [358, 359] employed dual strategy to effectively induce a cALD phenotype in 8-week-old male *Abcd* knockout mice using a combination of cuprizone and EAE models. This model faithfully reproduces the essential pathological characteristics of human cALD, including persistent brain demyelination, disruption of blood-brain barrier integrity, infiltration of both innate and adaptive immune cells around blood vessels, and a marked increase in oxidative stress markers. In contrast to mice that experience spontaneous myelin regeneration following cuprizone exposure [360], this cALD model demonstrates more pronounced active demyelinating lesions that closely resemble those found in human cALD.

### Metachromatic Leukodystrophy: ASA-deficient model

Metachromatic leukodystrophy **(**MLD) is a rare autosomal recessive neurodegenerative disorder, with most cases caused by pathological variants in the arylsulfatase A gene (*ARSA*), encoding arylsulfatase A enzyme (ASA) [361]. Clinical investigations utilizing cranial magnetic resonance imaging (MRI) demonstrated significant white matter abnormalities within the corpus callosum, periventricular areas, and central white matter, while subcortical fibers remain largely unaffected [362]. Furthermore, various mechanistic studies indicate that the extensive demyelination and oligodendrocyte loss characteristic of MLD are closely linked to a chronic inflammatory response instigated by sulfatides [363]. This is evidenced by the abnormal buildup of sulfatides that triggers demyelination and neuronal injury in both the central and peripheral nervous systems (PNS) [364].

In 1996, the first *ASA*-deficient mouse model was created [365], revealing sulfatide accumulation patterns similar to those observed in human patients. This model exhibits ongoing cerebroside sulfate accumulation in the CNS and is linked to behavioral disorders in adulthood. Hence, this model is widely acknowledged as a prototypical murine model for late infantile or early juvenile MLD in humans [366], and is frequently employed to assess the efficacy of novel MLD therapies [367, 368]. However, *ASA*-deficient mice do not display pathological features of demyelination [362, 369, 370] until after two years of age, while the disease progression is considerably slower than that seen in humans [365]. To address the limitations regarding demyelination, an MLD mouse model was modified by using the transgenic ASA-null [tg/ASA^-/-^] mice, which overexpresses galactose-3-O-sulfotransferase-1 in myelinating cells. By crossing these transgenic mice with *ASA*-deficient mice, the disease phenotype was effectively intensified. These mice showed a significant increase in brain cerebroside sulfate level, along with clear demyelination in both the CNS and PNS [371].

### Cerebral autosomal dominant arteriopathy with subcortical infarcts and leukoencephalopathy: Notch3 transgenic mouse model

Mutations of the gene encoding NOTCH3 receptor may result in cerebral autosomal dominant arteriopathy with subcortical infarcts and leukoencephalopathy (CADASIL), a disorder primarily marked by the occurrence of strokes and dementia. The clinical features of CADASIL are often evident on brain MRI, displaying characteristic extensive white matter hyperintensities particularly in the external capsule and anterior temporal pole areas [347].

A CADASIL mouse model was developed by incorporating a pathogenic point mutation in the *Notch3* gene, which is linked to CADASIL, into a large P1-derived artificial chromosome (PAC). This model effectively reproduces the endogenous expression pattern of NOTCH3 and the key pathological characteristics of CADASIL, including the accumulation of the NOTCH3 extracellular domain in cerebral blood vessels, the deposition of granular osmiophilic material, a gradual decline in white matter integrity, and diminished brain perfusion [372, 373]. Notably, the Arg169Cys NOTCH3 mutant (TgPAC-NOTCH3^R169C^) mouse model, which integrates the NOTCH3 point mutation into the PAC [372], demonstrates the development of small vacuoles within myelin sheaths (with diameters not exceeding 1 μm), which are closely linked to localized myelin degradation and vacuolation, particularly in white matter regions such as the corpus callosum, anterior commissure, hippocampal fimbria, internal capsule, and striatum [374]. However, it is crucial to highlight that, despite successfully modeling numerous features of CADASIL, this model does not exhibit lacunar infarcts [372]. Other NOTCH3 transgenic mouse models, which overexpress NOTCH3 with mutations, were also reported, including TgNOTCH3^R182C^, TgNOTCH3^R90C^, and TgNOTCH3^R169C^ [375–377]. Among them, the TgNOTCH3^R169C^ model, is characterized by the overexpression of the human R169C-mutated NOTCH3 protein within an FVB/N genetic background [377]. This model not only exhibits CADASIL-specific vascular NOTCH3 aggregation, but also reveals the presence of microinfarcts and subsequent lesions in white matter [378, 379].

### Alexander disease: Gfap transgenic and knockin mouse models

Alexander disease (AxD) is a leukodystrophy caused by sporadic GFAP mutations, being hence primary astrocytopathy [380]. Oligodendroglial impairment and myelin degeneration, however, are critical contributors to pathogenesis AxD [346]. Briefly, murine models of AxD include transgenic (TG, such as mouse GFAP promoter-human GFAP cDNA with R239H mutation) mice carrying multiple copies of human GFAP [381, 382] and knockin (KI, such as mice with GFAP-R76H and -R236H mutations) mice with a GFAP missense mutation homologous to human AxD [383, 384]. While these models exhibit marked astrocytic pathology including GFAP accumulation and Rosenthal fiber formation, white matter and oligodendrocyte abnormalities are less severe than in human AxD [381–383]. For instance, KI mice overexpressing mutant human GFAP (e.g., R239H, R76H) exhibit normal white matter architecture and myelination [383]. However, TG and KI mice (≥6 months) display subtle myelin defects, notably swollen structures positive for myelin markers, which may represent axonal spheroids [346]; while oligodendrocyte apoptosis emerges in older mice, aligning with the age-dependent progression of AxD. In contrast, when TG mice are crossed with KI mice, pronounced oligodendrocyte loss and reduced spinal cord MBP levels resembling human AxD pathology were detected [346].

### Recapitulation

Together, rodent models of genetic leukodystrophies (e.g., X-ALD, MLD, CADASIL, AxD) provide mechanistic insights but incompletely reproduce human disease complexity. X-ALD *Abcd1*-konckout mice mirror VLCFA accumulation yet require external triggers for inflammatory demyelination [355]. MLD models demonstrate sulfatide-induced demyelination but diverge temporally from human pathology [362, 369, 370]. CADASIL *Notch3* mutants mimic vascular aggregation and white matter vacuolation without lacunar infarcts [378, 379]. AxD *Gfap*-transgenic and knockin mice reveal mild oligodendroglial malfunction and myelin defects but fail to reproduce advanced white matter degeneration [381–384]. Integrating spatially resolved omics and multifactorial approaches (genetic, environmental, and combinatorial interventions) is essential to better mimic disease progression and enhance translational relevance.

## Non-rodent models in studying human white matter pathology

### Zebrafish

Zebrafish (*Danio rerio*) offers essential evolutionary and translational insights into white matter biology. Zebrafish genetic tractability, optical transparency for live imaging, and conserved myelination mechanisms make this specie particularly useful for mechanistic studies of white matter injury. For instance, zebrafish larvae bearing fluorescent reporters enable visualization of oligodendroglia dynamics during development [385–387]. In adult zebrafish models of demyelination, damage can be induced by injecting lysophosphatidylcholine into small gelatin pieces located near the optic nerve [388], or directly into the dorsal spinal cord of zebrafish [389, 390]. Another established method for inducing demyelination in zebrafish involves bacterial nitroreductase (NTR) to convert the non-toxic pro-drug metronidazole into a cytotoxic agent under the regulation of a cell-specific promoter [391]. Several transgenic zebrafish lines have been developed to ablate oligodendrocytes, including Mbp:Gal4; UAS:nfsB-mcherry, Sox10:Gal4; UAS:nfsB-mcherry [392], and Mbp:nfsB-eGFP [393]. Additionally, under specific light illumination conditions, the KillerRed photosensitizer generates reactive oxygen species, which allows precise and rapid deletion of oligodendroglial cells [394]. However, the use of zebrafish models for studying white matter pathology also faces challenges: a zebrafish EAE model induced by MOG35-55 was developed for in vivo drug screening, but the factors driving autoimmunity remain unknown since MOG has yet to be identified in their genome [395].

### Ferrets

Ferrets (*Mustelo furo*), being gyrencephalic mammals have complex cortical folding, myelination patterns, and intricate social behaviors all relevant for replicating human pathologies [396, 397]. This makes ferret ideal for developmental studies of white matter, especially in investigating myelin pathologies related to developmental disorders, such as encephalopathy of prematurity and immature hydrocephalus [398, 399]. Hypoxia in neonatal ferrets induces an increase in OPCs numbers, vessel density and vessel branching in the white matter tracts, mirroring the condition of periventricular leukomalacia in preterm infants. This indicates a dependence on OPCs-endothelial cell interactions for neonatal white matter vascular development in white matter injury [400]. Studies on cortical folding in ferrets shed light on the mechanisms of white matter expansion, offering insights into human neurodevelopmental disorders such as autism [401]. Furthermore, ferrets are useful for modeling traumatic axonal injury, demonstrating that white matter damage can trigger neuroinflammation similar to human traumatic brain injury [402, 403].

### Non-human primates

Comparative studies of non-human primates (NHPs) highlight evolutionary divergence where primates exhibit higher white matter-to-gray matter ratios than rodents and have lifespan trajectories that closely resemble that of humans. Hence, studies on NHPs provide more translational insights than rodent models. For instance, magnetic resonance imaging (MRI) studies in rhesus macaques (*Macaca mulatta*) reveal age-related white matter changes associated with both intracortical and subcortical myelin pathologies [404]. Early-life stress in infant macaques leads to reduced white matter integrity in adolescence, which correlates with elevated cortisol levels and behavioral deficits [405–407]. Rhesus monkeys sensitized with homologous spinal cord homogenates or MBP purified from human brains and adjuvanted with CFA rapidly develop severe hyperacute neurological syndromes, leading to rapid deterioration and death. These pathological manifestations resemble the clinical signs of ADEM more than those of MS [408]. Due to the genetic and immunological similarity of common marmosets (*Callithrix jacchus*) and humans, EAE models in marmosets mimic human autoimmune demyelination, and provide a unique experimental platform for investigating the immunopathogenetic mechanisms of MS [409]. Furthermore, white matter demyelination has been shown to precede axonal injury following ischemic stroke in cynomolgus (or crab-eating) monkeys (*Macaca fascicularis*), highlighting the potential of NHP models to explore adaptive mechanisms after stroke [410, 411].

### Large animal models

Large animal models, such as pigs and sheep, are used for translational neuroimaging and surgical interventions. For instance, mini-pigs, including sinclair™ miniature pigs (*Sus scrofa domestica*) and danish landrace pigs, have been used to model human adolescent brain white matter development, TBI, and focal stroke [412–414]. Sheep are used for studying early brain development and surgical manipulation; they were also employed to study cerebral white matter injury in hypoxic-ischemic encephalopathy, affecting full-term infants, and in periventricular leukomalacia, which impacts preterm infants [415–417].

### Ex vivo models

Rodent ex vivo models have been widely used to dissect the cell death signaling pathways underlying white matter injury and repair following injury. For example, the retinal explant model has demonstrated that Wnt pathway-mediated OPC-intrinsic hypoxia-inducible factor (HIF) signaling promotes endothelial tip sprouting, white matter angiogenesis, and the onset of myelination in the mammalian forebrain, particularly in response to hypoxic conditions [418]. The retina contains a single white matter tract, the optic nerve. The optic nerve crush model has advantages over the optic nerve transection model, as it is relatively mild and does not disrupt ocular blood flow, making it particularly useful for examining oligodendroglial injury and remyelination [419–422].

Ex vivo models also allow for the testing the efficacy of therapeutic agents. Rodent brain and spinal cord slice cultures [423–426], along with co-cultures of exogenous oligodendrocytes with unmyelinated tissue slices [427], are among the easiest methods to faithfully generate myelination, demyelination, and subsequent remyelination within a three-dimensional neuronal network [425, 426]. This ex vivo model provides straightforward access for evaluating potential therapeutic strategies, particularly for some blood-brain barrier (BBB)-impermeable drug precursors and peptides, due to its ease of use in cell biological, histological, and electrophysiological experiments [94, 428]. For instance, Wnt pathway inhibitor UM206 peptide, miconazole and clobetasol have been found to effectively promote myelination in organotypic cerebellar slice cultures [94, 429, 430]. Recently, human post-mortem organotypic brain slice cultures, perfectly recapitulating age- and disease-related characteristics in humans, may offer an alternative non-rodent tool to study pathomechanisms and test therapies [431].

## Human iPSC-derived organoids

By capturing human-specific genetics and cellular vulnerabilities, human induced pluripotent stem cell (iPSC)-derived brain organoids advanced studies of human white matter pathology [432, 433]. A landmark study generated myelinating organoids (termed ‘myelinoids’) from patients with monogenetic disease affecting myelinated axons (Nfasc155 deficiency), recapitulated impaired paranodal axo-glial junction formation [432]. Additionally, human iPSC-derived OPCs can be transplanted, contributing to robust remyelination of demyelinated axons in models of mouse spinal cord injury, rat stroke-injured brain, and adult human cortical slices [434, 435].

This in vitro system also allows for high-throughput drug screening [436]. For example, 1,300 iPSC-derived organoids were obtained from 11 sporadic AD patients to identify FDA-approved drugs as candidates for repurposing [437]. Cocultures of motor neuron spheroids from a patient with sporadic ALS and three-dimensional muscle fiber bundles have been used to replicate ALS pathology, and test the efficacy of candidate drugs already used in phase 1 and 2 clinical trials [438].

Despite these advances, challenges exist in the study of human iPSC-derived organoids. Limitations in vascularization within organoids restrict nutrient diffusion, affecting long-term myelination studies. Additionally, standardizing protocols for cell subtype generation remains critical for disease-specific modeling [402, 403, 432, 433].

## Conclusions and perspectives

The prevalence of white matter injury-related diseases in humans shows continuous increase [439]. Investigating the pathogenesis of these diseases and developing new pharmacological treatments through improved animal models is essential. This review provides a comprehensive resource by detailing the features, benefits, and limitations of different rodent models used in studies of white matter pathologies across wide range of diseases.

This review also raises questions regarding model development that require attention: (i) How can we modify rodent models at genetic and cellular levels to make it more representative of the pathological processes of human diseases? (ii) As clinical sample research becomes more popular, a growing number of pathological signatures, molecular alterations, and etiological mechanisms of diseases are identified. However, it raises the question of whether rodent models should be continuously refined according to these new findings. The challenge lies in determining how to effectively enhance these models. While such optimizations may help make the models more closely resemble the diseases they aim to study, substantial effort is also required. Moreover, it remains uncertain whether the biological differences between humans and rodents can be sufficiently minimized. (iii) With advancements in research technology, models such as NHPs, humanized mouse models, and human-iPSC organoids are integrated into the study of human diseases. These developments promise to enhance our understanding of white matter diseases in humans and represent a critical focus for future research. (iv) Thanks to advancements ‘in silico’, utilizing computer and machine learning to complement traditional in vivo and in vitro techniques, computer simulations and informatics can be used to predict drug effects, cell-to-cell communication, and disease progression. This innovative approach not only optimizes experimental protocols for animal experiments but also minimizes the need for unnecessary animal testing. Additionally, it may address the time-consuming limitations associated with conventional animal models [440, 441].

In the end, the authors would like to express gratitude to all experimental animals that have endured suffering and dedicated their lives for the advancement of research in human diseases.

## References

[1] Boyle R New experiments physico-mechanical, touching the spring of the air, and its effects, : (made, for the most part, in a new pneumatical engine) written by way of letter to the Right Honorable Charles Lord Vicount of Dungarvan, eldest son to the Earl of Corke. In: Line F, Hobbes T, Hall H, Lindsey G, Sachs AP, editors. The second edition. ed. Oxford : Printed by H. Hall, printer to the University, for Tho: Robinson; (1660).

[2] West JB. Robert Boyle’s landmark book of 1660 with the first experiments on rarified air. J Appl Physiol (1985). 2005;98:31–39. 15591301 10.1152/japplphysiol.00759.2004

[3] West JB. Robert Hooke: early respiratory physiologist, polymath, and mechanical genius. Physiology. 2014;29:222–33. 24985326 10.1152/physiol.00005.2014

[4] Pennisi E. A mouse chronology. Science. 2000;288:248–57. 10.1126/science.288.5464.248b 10777402

[5] Ellenbroek B, Youn J. Rodent models in neuroscience research: is it a rat race?. Dis Model Mech. 2016;9:1079–87. 27736744 10.1242/dmm.026120 PMC5087838

[6] Marner L, Nyengaard JR, Tang Y, Pakkenberg B. Marked loss of myelinated nerve fibers in the human brain with age. J Comp Neurol. 2003;462:144–52. 12794739 10.1002/cne.10714

[7] Pakkenberg B, Pelvig D, Marner L, Bundgaard MJ, Gundersen HJ, Nyengaard JR, et al. Aging and the human neocortex. Exp Gerontol. 2003;38:95–99. 12543266 10.1016/s0531-5565(02)00151-1

[8] Zhang K, Sejnowski TJ. A universal scaling law between gray matter and white matter of cerebral cortex. Proc Natl Acad Sci USA. 2000;97:5621–6. 10792049 10.1073/pnas.090504197 PMC25878

[9] Piekarski DJ, Zahr NM, Zhao Q, Ferizi U, Pohl KM, Sullivan EV, et al. White matter microstructural integrity continues to develop from adolescence to young adulthood in mice and humans: Same phenotype, different mechanism. Neuroimage Rep. 2023;3:100179. 10.1016/j.ynirp.2023.100179 PMC10619509 37916059

[10] Tirapu-Ustárroz J, Luna-Lario P, Hernáez-Goñi P, García-Suescun I. [Relation between white matter and cognitive functions]. Rev Neurol. 2011;52:725–42. 21594858

[11] Baumann N, Pham-Dinh D. Biology of oligodendrocyte and myelin in the mammalian central nervous system. Physiol Rev. 2001;81:871–927. 11274346 10.1152/physrev.2001.81.2.871

[12] Raine CS. On the association between perinodal astrocytic processes and the node of Ranvier in the C.N.S. J Neurocytol. 1984;13:21–27. 6707711 10.1007/BF01148316

[13] Merrill JE, Scolding NJ. Mechanisms of damage to myelin and oligodendrocytes and their relevance to disease. Neuropathol Appl Neurobiol. 1999;25:435–58. 10632895 10.1046/j.1365-2990.1999.00200.x

[14] Festa LK, Grinspan JB, Jordan-Sciutto KL. White matter injury across neurodegenerative disease. Trends Neurosci. 2024;47:47–57. 38052682 10.1016/j.tins.2023.11.003 PMC10842057

[15] Zhang H, Yang Y, Zhang J, Huang L, Niu Y, Chen H, et al. Oligodendrocytes play a critical role in white matter damage of vascular dementia. Neuroscience. 2024;538:1–10. 37913862 10.1016/j.neuroscience.2023.10.018

[16] Rosenberg GA. Willis Lecture: Biomarkers for inflammatory white matter injury in Binswanger disease provide pathways to precision medicine. Stroke. 2022;53:3514–23. 36148658 10.1161/STROKEAHA.122.039211 PMC9613611

[17] Braun M, Vaibhav K, Saad NM, Fatima S, Vender JR, Baban B, et al. White matter damage after traumatic brain injury: A role for damage associated molecular patterns. Biochim Biophys Acta Mol Basis Dis. 2017;1863:2614–26. 28533056 10.1016/j.bbadis.2017.05.020 PMC5653450

[18] Dutta R, Trapp BD. Mechanisms of neuronal dysfunction and degeneration in multiple sclerosis. Prog Neurobiol. 2011;93:1–12. 20946934 10.1016/j.pneurobio.2010.09.005 PMC3030928

[19] Xin W, Chan JR. Myelin plasticity: sculpting circuits in learning and memory. Nat Rev Neurosci. 2020;21:682–94. 33046886 10.1038/s41583-020-00379-8 PMC8018611

[20] Khelfaoui H, Ibaceta-Gonzalez C, Angulo MC. Functional myelin in cognition and neurodevelopmental disorders. Cell Mol Life Sci. 2024;81:181. 38615095 10.1007/s00018-024-05222-2 PMC11016012

[21] Grotheer M, Rosenke M, Wu H, Kular H, Querdasi FR, Natu VS, et al. White matter myelination during early infancy is linked to spatial gradients and myelin content at birth. Nat Commun. 2022;13:997. 35194018 10.1038/s41467-022-28326-4 PMC8863985

[22] Franklin RJM, Bodini B, Goldman SA. Remyelination in the central nervous system. Cold Spring Harb Perspect Biol. 2024;16:a041371. 10.1101/cshperspect.a041371 PMC10910446 38316552

[23] Alizadeh A, Dyck SM, Karimi-Abdolrezaee S. Myelin damage and repair in pathologic CNS: challenges and prospects. Front Mol Neurosci. 2015;8:35. 26283909 10.3389/fnmol.2015.00035 PMC4515562

[24] Franklin RJ, Ffrench-Constant C. Remyelination in the CNS: from biology to therapy. Nat Rev Neurosci. 2008;9:839–55. 18931697 10.1038/nrn2480

[25] Brown RA, Narayanan S, Arnold DL. Imaging of repeated episodes of demyelination and remyelination in multiple sclerosis. Neuroimage Clin. 2014;6:20–25. 25610760 10.1016/j.nicl.2014.06.009 PMC4299955

[26] Chen JF, Wang F, Huang NX, Xiao L, Mei F. Oligodendrocytes and myelin: Active players in neurodegenerative brains?. Dev Neurobiol. 2022;82:160–74. 35081276 10.1002/dneu.22867

[27] Kalafatakis I, Karagogeos D. Oligodendrocytes and Microglia: Key Players in Myelin Development, Damage and Repair. Biomolecules. 2021;11:1058. 10.3390/biom11071058 PMC8301746 34356682

[28] López-Muguruza E, Matute C. Alterations of Oligodendrocyte and Myelin Energy Metabolism in Multiple Sclerosis. Int J Mol Sci. 2023;24:12912. 10.3390/ijms241612912 PMC10454078 37629092

[29] Wang Q, Huang T, Zheng Z, Su Y, Wu Z, Zeng C, et al. Oligodendroglial precursor cells modulate immune response and early demyelination in a murine model of multiple sclerosis. Sci Transl Med. 2025;17:eadn9980. 40173259 10.1126/scitranslmed.adn9980

[30] Fang L, Kang X, Wang Z, Wang S, Wang J, Zhou Y, et al. Myelin Oligodendrocyte Glycoprotein-IgG Contributes to Oligodendrocytopathy in the presence of complement, distinct from astrocytopathy induced by AQP4-IgG. Neurosci Bull. 2019;35:853–66. 31041694 10.1007/s12264-019-00375-8 PMC6754494

[31] Baaklini CS, Rawji KS, Duncan GJ, Ho MFS, Plemel JR. Central nervous system remyelination: roles of Glia and innate immune cells. Front Mol Neurosci. 2019;12:225. 31616249 10.3389/fnmol.2019.00225 PMC6764409

[32] Zveik O, Rechtman A, Ganz T, Vaknin-Dembinsky A. The interplay of inflammation and remyelination: rethinking MS treatment with a focus on oligodendrocyte progenitor cells. Mol Neurodegener. 2024;19:53. 38997755 10.1186/s13024-024-00742-8 PMC11245841

[33] Semple BD, Blomgren K, Gimlin K, Ferriero DM, Noble-Haeusslein LJ. Brain development in rodents and humans: Identifying benchmarks of maturation and vulnerability to injury across species. Prog Neurobiol. 2013;106-107:1–16. 23583307 10.1016/j.pneurobio.2013.04.001 PMC3737272

[34] Smith AM, Dragunow M. The human side of microglia. Trends Neurosci. 2014;37:125–35. 24388427 10.1016/j.tins.2013.12.001

[35] Lambert C, Desbarats J, Arbour N, Hall JA, Olivier A, Bar-Or A, et al. Dendritic cell differentiation signals induce anti-inflammatory properties in human adult microglia. J Immunol. 2008;181:8288–97. 19050245 10.4049/jimmunol.181.12.8288

[36] Re F, Belyanskaya SL, Riese RJ, Cipriani B, Fischer FR, Granucci F, et al. Granulocyte-macrophage colony-stimulating factor induces an expression program in neonatal microglia that primes them for antigen presentation. J Immunol. 2002;169:2264–73. 12193691 10.4049/jimmunol.169.5.2264

[37] Esen N, Kielian T. Effects of low dose GM-CSF on microglial inflammatory profiles to diverse pathogen-associated molecular patterns (PAMPs). J Neuroinflammation. 2007;4:10. 17374157 10.1186/1742-2094-4-10 PMC1839084

[38] Bjornson-Hooper ZB, Fragiadakis GK, Spitzer MH, Chen H, Madhireddy D, Hu K, et al. A Comprehensive Atlas of immunological differences between humans, mice, and non-human primates. Front Immunol. 2022;13:867015. 35359965 10.3389/fimmu.2022.867015 PMC8962947

[39] Weisel F, Shlomchik M. Memory B Cells of Mice and Humans. Annu Rev Immunol. 2017;35:255–84. 28142324 10.1146/annurev-immunol-041015-055531

[40] Mitsdoerffer M, Kuchroo V, Korn T. Immunology of neuromyelitis optica: a T cell-B cell collaboration. Ann N Y Acad Sci. 2013;1283:57–66. 23617588 10.1111/nyas.12118 PMC3963833

[41] Kuchroo VK, Martin CA, Greer JM, Ju ST, Sobel RA, Dorf ME. Cytokines and adhesion molecules contribute to the ability of myelin proteolipid protein-specific T cell clones to mediate experimental allergic encephalomyelitis. J Immunol. 1993;151:4371–82. 7691946

[42] Park H, Li Z, Yang XO, Chang SH, Nurieva R, Wang YH, et al. A distinct lineage of CD4 T cells regulates tissue inflammation by producing interleukin 17. Nat Immunol. 2005;6:1133–41. 16200068 10.1038/ni1261 PMC1618871

[43] Desai MK, Sudol KL, Janelsins MC, Mastrangelo MA, Frazer ME, Bowers WJ. Triple-transgenic Alzheimer’s disease mice exhibit region-specific abnormalities in brain myelination patterns prior to appearance of amyloid and tau pathology. Glia. 2009;57:54–65. 18661556 10.1002/glia.20734 PMC2584762

[44] Lee JT, Xu J, Lee JM, Ku G, Han X, Yang DI, et al. Amyloid-beta peptide induces oligodendrocyte death by activating the neutral sphingomyelinase-ceramide pathway. J Cell Biol. 2004;164:123–31. 14709545 10.1083/jcb.200307017 PMC2171973

[45] Correale J, Gaitán MI, Ysrraelit MC, Fiol MP. Progressive multiple sclerosis: from pathogenic mechanisms to treatment. Brain. 2017;140:527–46. 27794524 10.1093/brain/aww258

[46] Correale J, Ysrraelit MC. Multiple Sclerosis and Aging: The Dynamics of Demyelination and Remyelination. ASN Neuro. 2022;14:17590914221118502. 35938615 10.1177/17590914221118502 PMC9364177

[47] McMurran CE, Mukherjee T, Brown JWL, Michell AW, Chard DT, Franklin RJM, et al. Remyelination in humans due to a retinoid-X receptor agonist is age-dependent. Ann Clin Transl Neurol. 2022;9:1090–4. 35587315 10.1002/acn3.51595 PMC9268872

[48] Gingele S, Henkel F, Heckers S, Moellenkamp TM, Hümmert MW, Skripuletz T, et al. Delayed Demyelination and Impaired Remyelination in Aged Mice in the Cuprizone Model. Cells. 2020;9:945. 10.3390/cells9040945 PMC7226973 32290524

[49] Procaccini C, De Rosa V, Pucino V, Formisano L, Matarese G. Animal models of Multiple Sclerosis. Eur J Pharm. 2015;759:182–91. 10.1016/j.ejphar.2015.03.042 PMC7094661 25823807

[50] Rone MB, Cui QL, Fang J, Wang LC, Zhang J, Khan D, et al. Oligodendrogliopathy in multiple sclerosis: low glycolytic metabolic rate promotes oligodendrocyte survival. J Neurosci. 2016;36:4698–707. 27122029 10.1523/JNEUROSCI.4077-15.2016 PMC6601725

[51] Huang S, Ren C, Luo Y, Ding Y, Ji X, Li S. New insights into the roles of oligodendrocytes regulation in ischemic stroke recovery. Neurobiol Dis. 2023;184:106200. 37321419 10.1016/j.nbd.2023.106200

[52] Fernandes MGF, Mohammadnia A, Pernin F, Schmitz-Gielsdorf LE, Hodgins C, Cui QL, et al. Mechanisms of metabolic stress induced cell death of human oligodendrocytes: relevance for progressive multiple sclerosis. Acta Neuropathol Commun. 2023;11:108. 37408029 10.1186/s40478-023-01601-1 PMC10320974

[53] Matute C, Alberdi E, Domercq M, Pérez-Cerdá F, Pérez-Samartín A, Sánchez-Gómez MV. The link between excitotoxic oligodendroglial death and demyelinating diseases. Trends Neurosci. 2001;24:224–30. 11250007 10.1016/s0166-2236(00)01746-x

[54] Deng W, Wang H, Rosenberg PA, Volpe JJ, Jensen FE. Role of metabotropic glutamate receptors in oligodendrocyte excitotoxicity and oxidative stress. Proc Natl Acad Sci USA. 2004;101:7751–6. 15136737 10.1073/pnas.0307850101 PMC419678

[55] Matute C, Torre I, Pérez-Cerdá F, Pérez-Samartín A, Alberdi E, Etxebarria E, et al. P2X(7) receptor blockade prevents ATP excitotoxicity in oligodendrocytes and ameliorates experimental autoimmune encephalomyelitis. J Neurosci. 2007;27:9525–33. 17728465 10.1523/JNEUROSCI.0579-07.2007 PMC6673129

[56] Deng W, Yue Q, Rosenberg PA, Volpe JJ, Jensen FE. Oligodendrocyte excitotoxicity determined by local glutamate accumulation and mitochondrial function. J Neurochem. 2006;98:213–22. 16606353 10.1111/j.1471-4159.2006.03861.x

[57] Ruiz A, Matute C, Alberdi E. Intracellular Ca2+ release through ryanodine receptors contributes to AMPA receptor-mediated mitochondrial dysfunction and ER stress in oligodendrocytes. Cell Death Dis. 2010;1:e54. 21364659 10.1038/cddis.2010.31 PMC3032558

[58] McDonald JW, Althomsons SP, Hyrc KL, Choi DW, Goldberg MP. Oligodendrocytes from forebrain are highly vulnerable to AMPA/kainate receptor-mediated excitotoxicity. Nat Med. 1998;4:291–7. 9500601 10.1038/nm0398-291

[59] Bernal-Chico A, Manterola A, Cipriani R, Katona I, Matute C, Mato S. P2x7 receptors control demyelination and inflammation in the cuprizone model. Brain Behav Immun Health. 2020;4:100062. 34589847 10.1016/j.bbih.2020.100062 PMC8474271

[60] Barcelos IP, Troxell RM, Graves JS. Mitochondrial dysfunction and multiple sclerosis. Biology (Basel). 2019;8:37. 10.3390/biology8020037 PMC6627385 31083577

[61] Ferreira HB, Neves B, Guerra IM, Moreira A, Melo T, Paiva A, et al. An overview of lipidomic analysis in different human matrices of multiple sclerosis. Mult Scler Relat Disord. 2020;44:102189. 32516740 10.1016/j.msard.2020.102189

[62] Chapman TW, Hill RA. Myelin plasticity in adulthood and aging. Neurosci Lett. 2020;715:134645. 31765728 10.1016/j.neulet.2019.134645 PMC6981290

[63] Shen S, Sandoval J, Swiss VA, Li J, Dupree J, Franklin RJ, et al. Age-dependent epigenetic control of differentiation inhibitors is critical for remyelination efficiency. Nat Neurosci. 2008;11:1024–34. 19160500 10.1038/nn.2172 PMC2656679

[64] Breschi A, Gingeras TR, Guigó R. Comparative transcriptomics in human and mouse. Nat Rev Genet. 2017;18:425–40. 28479595 10.1038/nrg.2017.19 PMC6413734

[65] Lin S, Lin Y, Nery JR, Urich MA, Breschi A, Davis CA, et al. Comparison of the transcriptional landscapes between human and mouse tissues. Proc Natl Acad Sci USA. 2014;111:17224–9. 25413365 10.1073/pnas.1413624111 PMC4260565

[66] Yue F, Cheng Y, Breschi A, Vierstra J, Wu W, Ryba T, et al. A comparative encyclopedia of DNA elements in the mouse genome. Nature. 2014;515:355–64. 25409824 10.1038/nature13992 PMC4266106

[67] Espino-Paisán L, Agudo-Jiménez T, Rosales-Martínez I, López-Cotarelo P, García-Martínez M, Domínguez-Mozo MI, et al. A polymorphism within the MBP gene is associated with a higher relapse number in male patients of multiple sclerosis. Front Immunol. 2020;11:771. 32431704 10.3389/fimmu.2020.00771 PMC7214696

[68] Perry GH. The evolutionary significance of copy number variation in the human genome. Cytogenet Genome Res. 2008;123:283–7. 19287166 10.1159/000184719 PMC2920192

[69] Pös O, Radvanszky J, Buglyó G, Pös Z, Rusnakova D, Nagy B, et al. DNA copy number variation: Main characteristics, evolutionary significance, and pathological aspects. Biomed J. 2021;44:548–59. 34649833 10.1016/j.bj.2021.02.003 PMC8640565

[70] Confavreux C, Vukusic S. Age at disability milestones in multiple sclerosis. Brain. 2006;129:595–605. 16415309 10.1093/brain/awh714

[71] Torre-Fuentes L, Moreno-Jiménez L, Pytel V, Matías-Guiu JA, Gómez-Pinedo U, Matías-Guiu J. Experimental models of demyelination and remyelination. Neurologia (Engl Ed). 2020;35:32–39. 28863829 10.1016/j.nrl.2017.07.002 PMC7115679

[72] Ransohoff RM. Animal models of multiple sclerosis: the good, the bad and the bottom line. Nat Neurosci. 2012;15:1074–7. 22837037 10.1038/nn.3168 PMC7097342

[73] Bradl M, Linington C. Animal models of demyelination. Brain Pathol. 1996;6:303–11. 8864286 10.1111/j.1750-3639.1996.tb00857.x PMC7161775

[74] Gold R, Hartung HP, Toyka KV. Animal models for autoimmune demyelinating disorders of the nervous system. Mol Med Today. 2000;6:88–91. 10652482 10.1016/s1357-4310(99)01639-1

[75] Filippi M, Bar-Or A, Piehl F, Preziosa P, Solari A, Vukusic S, et al. Multiple sclerosis. Nat Rev Dis Prim. 2018;4:43. 30410033 10.1038/s41572-018-0041-4

[76] Dutra BG, da Rocha AJ, Nunes RH, Maia ACMJ. Neuromyelitis Optica Spectrum Disorders: Spectrum of MR Imaging Findings and Their Differential Diagnosis. Radiographics. 2018;38:169–93. 29320331 10.1148/rg.2018170141

[77] De Strooper B, Karran E. The Cellular Phase of Alzheimer’s Disease. Cell. 2016;164:603–15. 26871627 10.1016/j.cell.2015.12.056

[78] van der Knaap MS, Bugiani M. Leukodystrophies: a proposed classification system based on pathological changes and pathogenetic mechanisms. Acta Neuropathol. 2017;134:351–82. 28638987 10.1007/s00401-017-1739-1 PMC5563342

[79] Lublin FD, Reingold SC, Cohen JA, Cutter GR, Sorensen PS, Thompson AJ, et al. Defining the clinical course of multiple sclerosis: the 2013 revisions. Neurology. 2014;83:278–86. 24871874 10.1212/WNL.0000000000000560 PMC4117366

[80] Barkhof F, Koeller KK IDKD Springer Series Demyelinating Diseases of the CNS (Brain and Spine). In: Hodler J, Kubik-Huch RA, von Schulthess GK (eds). Diseases of the Brain, Head and Neck, Spine 2020–2023: Diagnostic Imaging. Springer Copyright 2020, The Author(s). Cham (CH), 2020, pp 165-76.

[81] Dedoni S, Scherma M, Camoglio C, Siddi C, Dazzi L, Puliga R, et al. An overall view of the most common experimental models for multiple sclerosis. Neurobiol Dis. 2023;184:106230. 37453561 10.1016/j.nbd.2023.106230

[82] Hasselmann JPC, Karim H, Khalaj AJ, Ghosh S, Tiwari-Woodruff SK. Consistent induction of chronic experimental autoimmune encephalomyelitis in C57BL/6 mice for the longitudinal study of pathology and repair. J Neurosci Methods. 2017;284:71–84. 28396177 10.1016/j.jneumeth.2017.04.003 PMC5749979

[83] Constantinescu CS, Farooqi N, O’Brien K, Gran B. Experimental autoimmune encephalomyelitis (EAE) as a model for multiple sclerosis (MS). Br J Pharm. 2011;164:1079–106. 10.1111/j.1476-5381.2011.01302.x PMC3229753 21371012

[84] Zhao CB, Coons SW, Cui M, Shi FD, Vollmer TL, Ma CY, et al. A new EAE model of brain demyelination induced by intracerebroventricular pertussis toxin. Biochem Biophys Res Commun. 2008;370:16–21. 18339308 10.1016/j.bbrc.2008.02.161

[85] Peiris M, Monteith GR, Roberts-Thomson SJ, Cabot PJ. A model of experimental autoimmune encephalomyelitis (EAE) in C57BL/6 mice for the characterisation of intervention therapies. J Neurosci Methods. 2007;163:245–54. 17477973 10.1016/j.jneumeth.2007.03.013

[86] Ucar EA, Ozkan E, Shomalizadeh N, Sekerdağ-Kilic E, Akpunar F, Sapanci S, et al. Carbenoxolone mitigates extensive fibrosis formation in PLP-induced EAE model and multiple sclerosis serum-exposed pericyte culture. Front Cell Neurosci. 2024;18:1403974. 38746079 10.3389/fncel.2024.1403974 PMC11091252

[87] Burrows DJ, McGown A, Jain SA, De Felice M, Ramesh TM, Sharrack B, et al. Animal models of multiple sclerosis: From rodents to zebrafish. Mult Scler. 2019;25:306–24. 30319015 10.1177/1352458518805246

[88] van der Meide PH, de Labie MC, Ruuls SR, Groenestein RJ, Botman CA, Olsson T, et al. Discontinuation of treatment with IFN-beta leads to exacerbation of experimental autoimmune encephalomyelitis in Lewis rats. Rapid reversal of the antiproliferative activity of IFN-beta and excessive expansion of autoreactive T cells as disease promoting mechanisms. J Neuroimmunol. 1998;84:14–23. 9600704 10.1016/s0165-5728(97)00207-5

[89] Bjelobaba I, Begovic-Kupresanin V, Pekovic S, Lavrnja I. Animal models of multiple sclerosis: Focus on experimental autoimmune encephalomyelitis. J Neurosci Res. 2018;96:1021–42. 29446144 10.1002/jnr.24224

[90] Muller DM, Pender MP, Greer JM. A neuropathological analysis of experimental autoimmune encephalomyelitis with predominant brain stem and cerebellar involvement and differences between active and passive induction. Acta Neuropathol. 2000;100:174–82. 10963365 10.1007/s004019900163

[91] t Hart BA, Gran B, Weissert R. EAE: imperfect but useful models of multiple sclerosis. Trends Mol Med. 2011;17:119–25. 21251877 10.1016/j.molmed.2010.11.006

[92] Robinson AP, Harp CT, Noronha A, Miller SD. The experimental autoimmune encephalomyelitis (EAE) model of MS: utility for understanding disease pathophysiology and treatment. Handb Clin Neurol. 2014;122:173–89. 24507518 10.1016/B978-0-444-52001-2.00008-X PMC3981554

[93] Papazian I, Kourouvani M, Dagkonaki A, Gouzouasis V, Dimitrakopoulou L, Markoglou N, et al. Spontaneous human CD8 T cell and autoimmune encephalomyelitis-induced CD4/CD8 T cell lesions in the brain and spinal cord of HLA-DRB1*15-positive multiple sclerosis humanized immune system mice. Elife. 2024;12:RP88826. 10.7554/eLife.88826 PMC11189630 38900149

[94] Niu J, Yu G, Wang X, Xia W, Wang Y, Hoi KK, et al. Oligodendroglial ring finger protein Rnf43 is an essential injury-specific regulator of oligodendrocyte maturation. Neuron. 2021;109:3104–18.e3106. 34390652 10.1016/j.neuron.2021.07.018 PMC8547708

[95] Niu J, Tsai HH, Hoi KK, Huang N, Yu G, Kim K, et al. Aberrant oligodendroglial-vascular interactions disrupt the blood-brain barrier, triggering CNS inflammation. Nat Neurosci. 2019;22:709–18. 30988524 10.1038/s41593-019-0369-4 PMC6486410

[96] Zhan J, Mann T, Joost S, Behrangi N, Frank M, Kipp M. The Cuprizone Model: Dos and Do Nots. Cells. 2020;9:843. 10.3390/cells9040843 PMC7226799 32244377

[97] Vega-Riquer JM, Mendez-Victoriano G, Morales-Luckie RA, Gonzalez-Perez O. Five decades of Cuprizone, an updated model to replicate demyelinating diseases. Curr Neuropharmacol. 2019;17:129–41. 28714395 10.2174/1570159X15666170717120343 PMC6343207

[98] Matsushima GK, Morell P. The neurotoxicant, cuprizone, as a model to study demyelination and remyelination in the central nervous system. Brain Pathol. 2001;11:107–16. 11145196 10.1111/j.1750-3639.2001.tb00385.x PMC8098267

[99] Leo H, Kipp M. Remyelination in multiple sclerosis: findings in the Cuprizone model. Int J Mol Sci. 2022;23:16093. 10.3390/ijms232416093 PMC9783537 36555733

[100] Soulika AM, Lee E, McCauley E, Miers L, Bannerman P, Pleasure D. Initiation and progression of axonopathy in experimental autoimmune encephalomyelitis. J Neurosci. 2009;29:14965–79. 19940192 10.1523/JNEUROSCI.3794-09.2009 PMC2990681

[101] Gharagozloo M, Mace JW, Calabresi PA. Animal models to investigate the effects of inflammation on remyelination in multiple sclerosis. Front Mol Neurosci. 2022;15:995477. 36407761 10.3389/fnmol.2022.995477 PMC9669474

[102] Gharagozloo M, Bannon R, Calabresi PA. Breaking the barriers to remyelination in multiple sclerosis. Curr Opin Pharm. 2022;63:102194. 10.1016/j.coph.2022.102194 PMC8995341 35255453

[103] Plemel JR, Michaels NJ, Weishaupt N, Caprariello AV, Keough MB, Rogers JA, et al. Mechanisms of lysophosphatidylcholine-induced demyelination: A primary lipid disrupting myelinopathy. Glia. 2018;66:327–47. 29068088 10.1002/glia.23245

[104] Bosch-Queralt M, Tiwari V, Damkou A, Vaculčiaková L, Alexopoulos I, Simons M. A fluorescence microscopy-based protocol for volumetric measurement of lysolecithin lesion-associated de- and re-myelination in mouse brain. STAR Protoc. 2022;3:101141. 35141565 10.1016/j.xpro.2022.101141 PMC8810560

[105] Hall SM. The effect of injections of lysophosphatidyl choline into white matter of the adult mouse spinal cord. J Cell Sci. 1972;10:535–46. 5018033 10.1242/jcs.10.2.535

[106] Blakemore WF, Franklin RJ. Remyelination in experimental models of toxin-induced demyelination. Curr Top Microbiol Immunol. 2008;318:193–212. 18219819 10.1007/978-3-540-73677-6_8

[107] Abboud H, Yu XX, Knusel K, Fernandez HH, Cohen JA. Movement disorders in early MS and related diseases: A prospective observational study. Neurol Clin Pr. 2019;9:24–31. 10.1212/CPJ.0000000000000560 PMC6382384 30859004

[108] Yamazaki R, Ohno N, Huang JK. Acute motor deficit and subsequent remyelination-associated recovery following internal capsule demyelination in mice. J Neurochem. 2021;156:917–28. 32750162 10.1111/jnc.15142 PMC8048697

[109] Yamazaki R, Ohno N. The Mouse Model of Internal Capsule Demyelination: A Novel Tool for Investigating Motor Functional Changes Caused by Demyelination and for Evaluating Drugs That Promote Remyelination. Acta Histochem Cytochem. 2024;57:1–5. 38463203 10.1267/ahc.24-00005 PMC10918433

[110] Wingerchuk DM. Neuromyelitis optica: new findings on pathogenesis. Int Rev Neurobiol. 2007;79:665–88. 17531863 10.1016/S0074-7742(07)79029-3

[111] Tsai CC, Combes A, McMullen K, Kolind SH, Traboulsee AL. Exploring subcortical pathology and processing speed in neuromyelitis optica spectrum disorder with myelin water imaging. J Neuroimaging. 2025;35:e13250. 39511966 10.1111/jon.13250 PMC11625695

[112] Höftberger R, Lassmann H. Inflammatory demyelinating diseases of the central nervous system. Handb Clin Neurol. 2017;145:263–83. 28987175 10.1016/B978-0-12-802395-2.00019-5 PMC7149979

[113] Zhang L, Verkhratsky A, Shi FD. Astrocytes and microglia in multiple sclerosis and neuromyelitis optica. Handb Clin Neurol. 2025;210:133–45. 40148041 10.1016/B978-0-443-19102-2.00001-6

[114] Verkhratsky A, Li B, Niu J, Lin SS, Su Y, Jin WN, et al. Neuroglial Advances: New Roles for Established Players. J Neurochem. 2025;169:e70080. 40371609 10.1111/jnc.70080

[115] Duan T, Verkman AS. Experimental animal models of aquaporin-4-IgG-seropositive neuromyelitis optica spectrum disorders: progress and shortcomings. Brain Pathol. 2020;30:13–25. 31587392 10.1111/bpa.12793 PMC7034663

[116] Huang TL, Wang JK, Chang PY, Hsu YR, Lin CH, Lin KH, et al. Neuromyelitis optica spectrum disorder: from basic research to clinical perspectives. Int J Mol Sci. 2022;23:7908. 10.3390/ijms23147908 PMC9323454 35887254

[117] Kurosawa K, Misu T, Takai Y, Sato DK, Takahashi T, Abe Y, et al. Severely exacerbated neuromyelitis optica rat model with extensive astrocytopathy by high affinity anti-aquaporin-4 monoclonal antibody. Acta Neuropathol Commun. 2015;3:82. 26637322 10.1186/s40478-015-0259-2 PMC4670539

[118] Bradl M, Lassmann H. Experimental models of neuromyelitis optica. Brain Pathol. 2014;24:74–82. 24345221 10.1111/bpa.12098 PMC4065348

[119] Xu L, Xu H, Tang C. Aquaporin-4-IgG-seropositive neuromyelitis optica spectrum disorders: progress of experimental models based on disease pathogenesis. Neural Regen Res. 2025;20:354–65. 38819039 10.4103/NRR.NRR-D-23-01325 PMC11317952

[120] Bennett JL, Lam C, Kalluri SR, Saikali P, Bautista K, Dupree C, et al. Intrathecal pathogenic anti-aquaporin-4 antibodies in early neuromyelitis optica. Ann Neurol. 2009;66:617–29. 19938104 10.1002/ana.21802 PMC3180961

[121] Saini H, Rifkin R, Gorelik M, Huang H, Ferguson Z, Jones MV, et al. Passively transferred human NMO-IgG exacerbates demyelination in mouse experimental autoimmune encephalomyelitis. BMC Neurol. 2013;13:104. 23927715 10.1186/1471-2377-13-104 PMC3750922

[122] Lee CL, Wang KC, Chen SJ, Chen CM, Tsai CP, Chen SY. Repetitive intrathecal injection of human NMO-IgG with complement exacerbates disease severity with NMO pathology in experimental allergic encephalomyelitis mice. Mult Scler Relat Disord. 2019;30:225–30. 30825702 10.1016/j.msard.2019.02.025

[123] Saadoun S, Waters P, Bell BA, Vincent A, Verkman AS, Papadopoulos MC. Intra-cerebral injection of neuromyelitis optica immunoglobulin G and human complement produces neuromyelitis optica lesions in mice. Brain. 2010;133:349–61. 20047900 10.1093/brain/awp309 PMC2822632

[124] Soerensen SF, Wirenfeldt M, Wlodarczyk A, Moerch MT, Khorooshi R, Arengoth DS, et al. An Experimental Model of Neuromyelitis Optica Spectrum Disorder-Optic Neuritis: Insights Into Disease Mechanisms. Front Neurol. 2021;12:703249. 34367056 10.3389/fneur.2021.703249 PMC8345107

[125] Zhang Y, Bao Y, Qiu W, Peng L, Fang L, Xu Y, et al. Structural and visual functional deficits in a rat model of neuromyelitis optica spectrum disorders related optic neuritis. Exp Eye Res. 2018;175:124–32. 29913164 10.1016/j.exer.2018.06.011

[126] Morita Y, Itokazu T, Nakanishi T, Hiraga SI, Yamashita T. A novel aquaporin-4-associated optic neuritis rat model with severe pathological and functional manifestations. J Neuroinflammation. 2022;19:263. 36303157 10.1186/s12974-022-02623-7 PMC9615200

[127] Marignier R, Ruiz A, Cavagna S, Nicole A, Watrin C, Touret M, et al. Neuromyelitis optica study model based on chronic infusion of autoantibodies in rat cerebrospinal fluid. J Neuroinflammation. 2016;13:111. 27193196 10.1186/s12974-016-0577-8 PMC4872335

[128] Jiang S, Li X, Li Y, Chang Z, Yuan M, Zhang Y, et al. APOE from patient-derived astrocytic extracellular vesicles alleviates neuromyelitis optica spectrum disorder in a mouse model. Sci Transl Med. 2024;16:eadg5116. 38416841 10.1126/scitranslmed.adg5116

[129] Sagan SA, Winger RC, Cruz-Herranz A, Nelson PA, Hagberg S, Miller CN, et al. Tolerance checkpoint bypass permits emergence of pathogenic T cells to neuromyelitis optica autoantigen aquaporin-4. Proc Natl Acad Sci USA. 2016;113:14781–6. 27940915 10.1073/pnas.1617859114 PMC5187685

[130] Serizawa K, Miyake S, Katsura Y, Yorozu K, Kurasawa M, Tomizawa-Shinohara H, et al. Intradermal AQP4 peptide immunization induces clinical features of neuromyelitis optica spectrum disorder in mice. J Neuroimmunol. 2023;380:578109. 37210799 10.1016/j.jneuroim.2023.578109

[131] Yasuda T, Ura T, Taniguchi M, Yoshida H. Intradermal Delivery of antigens enhances specific IgG and diminishes IgE production: potential use for vaccination and allergy immunotherapy. PLoS One. 2016;11:e0167952. 27973543 10.1371/journal.pone.0167952 PMC5156430

[132] da Silva APB, Silva RBM, Goi LDS, Molina RD, Machado DC, Sato DK. Experimental models of neuroimmunological disorders: a review. Front Neurol. 2020;11:389. 32477252 10.3389/fneur.2020.00389 PMC7235321

[133] Sagan SA, Moinfar Z, Moseley CE, Dandekar R, Spencer CM, Verkman AS, et al. T cell deletional tolerance restricts AQP4 but not MOG CNS autoimmunity. Proc Natl Acad Sci USA. 2023;120:e2306572120. 37463205 10.1073/pnas.2306572120 PMC10372680

[134] Asavapanumas N, Ratelade J, Verkman AS. Unique neuromyelitis optica pathology produced in naïve rats by intracerebral administration of NMO-IgG. Acta Neuropathol. 2014;127:539–51. 24190619 10.1007/s00401-013-1204-8 PMC3954950

[135] Xiang W, Xie C, Luo J, Zhang W, Zhao X, Yang H, et al. Low frequency ultrasound with injection of NMO-IgG and complement produces lesions different from experimental autoimmune Encephalomyelitis mice. Front Immunol. 2021;12:727750. 34721390 10.3389/fimmu.2021.727750 PMC8551829

[136] Oertel FC, Hastermann M, Paul F. Delimiting MOGAD as a disease entity using translational imaging. Front Neurol. 2023;14:1216477. 38333186 10.3389/fneur.2023.1216477 PMC10851159

[137] Utumi Y, Iseki E, Murayama N, Ichimiya Y, Arai H. [Limbic encephalitis caused by herpes simplex virus infection after vaccination against the influenza virus]. Brain Nerve. 2010;62:615–9. 20548122

[138] Niemeyer B, Niemeyer R, Borges R, Marchiori E. Acute disseminated Encephalomyelitis following Zika virus infection. Eur Neurol. 2017;77:45–46. 27894121 10.1159/000453396

[139] El Ouni F, Hassayoun S, Gaha M, Mhabrech H, Mrad-Dali K, Tlili K. Acute disseminated encephalomyelitis following herpes simplex encephalitis. Acta Neurol Belg. 2010;110:340–4. 21305866

[140] Stüve O, Nessler S, Hartung HP, Hemmer B, Wiendl H, Kieseier BC. [Acute disseminated encephalomyelitis. Pathogenesis, diagnosis, treatment, and prognosis]. Nervenarzt. 2005;76:701–7. 15580467 10.1007/s00115-004-1842-0

[141] Djabirska I, Delaval L, Tromme A, Blomet J, Desmecht D, Van Laere AS. Longitudinal quantitative assessment of TMEV-IDD-induced MS phenotypes in two inbred mouse strains using automated video tracking technology. Exp Neurol. 2024;379:114851. 38876197 10.1016/j.expneurol.2024.114851

[142] Leitzen E, Jin W, Herder V, Beineke A, Elmarabet SA, Baumgärtner W, et al. Comparison of reported spinal cord lesions in progressive multiple sclerosis with Theiler’s Murine Encephalomyelitis virus induced demyelinating disease. Int J Mol Sci. 2019;20:989. 10.3390/ijms20040989 PMC6413032 30823515

[143] Dunham J, van de Vis R, Bauer J, Wubben J, van Driel N, Laman JD, et al. Severe oxidative stress in an acute inflammatory demyelinating model in the rhesus monkey. PLoS One. 2017;12:e0188013. 29136024 10.1371/journal.pone.0188013 PMC5685592

[144] Schuh C, Wimmer I, Hametner S, Haider L, Van Dam AM, Liblau RS, et al. Oxidative tissue injury in multiple sclerosis is only partly reflected in experimental disease models. Acta Neuropathol. 2014;128:247–66. 24622774 10.1007/s00401-014-1263-5 PMC4102830

[145] Peters A. The effects of normal aging on myelin and nerve fibers: a review. J Neurocytol. 2002;31:581–93. 14501200 10.1023/a:1025731309829

[146] Bartzokis G. Alzheimer’s disease as homeostatic responses to age-related myelin breakdown. Neurobiol aging. 2011;32:1341–71. 19775776 10.1016/j.neurobiolaging.2009.08.007 PMC3128664

[147] Clayton BLL, Tesar PJ. Oligodendrocyte progenitor cell fate and function in development and disease. Curr Opin Cell Biol. 2021;73:35–40. 34153742 10.1016/j.ceb.2021.05.003 PMC8678156

[148] Caldwell M, Ayo-Jibunoh V, Mendoza JC, Brimblecombe KR, Reynolds LM, Zhu Jiang XY, et al. Axo-glial interactions between midbrain dopamine neurons and oligodendrocyte lineage cells in the anterior corpus callosum. Brain Struct Funct. 2023;228:1993–2006. 37668732 10.1007/s00429-023-02695-y PMC10516790

[149] Nussbaum RL, Ellis CE. Alzheimer’s disease and Parkinson’s disease. N Engl J Med. 2003;348:1356–64. 12672864 10.1056/NEJM2003ra020003

[150] Armstrong RA. The molecular biology of senile plaques and neurofibrillary tangles in Alzheimer’s disease. Folia Neuropathol. 2009;47:289–99. 20054780

[151] Ittner LM, Götz J. Amyloid-β and tau-a toxic pas de deux in Alzheimer’s disease. Nat Rev Neurosci. 2011;12:65–72. 10.1038/nrn2967 21193853

[152] Kawade N, Yamanaka K. Novel insights into brain lipid metabolism in Alzheimer’s disease: Oligodendrocytes and white matter abnormalities. FEBS Open Bio. 2024;14:194–216. 10.1002/2211-5463.13661 PMC10839347 37330425

[153] Moscoso A, Silva-Rodríguez J, Aldrey JM, Cortés J, Pías-Peleteiro JM, Ruibal Á, et al. 18) F-florbetapir PET as a marker of myelin integrity across the Alzheimer’s disease spectrum. Eur J Nucl Med Mol Imaging. 2022;49:1242–53. 10.1007/s00259-021-05493-y PMC8921113 34581847

[154] Nasrabady SE, Rizvi B, Goldman JE, Brickman AM. White matter changes in Alzheimer’s disease: a focus on myelin and oligodendrocytes. Acta Neuropathol Commun. 2018;6:22. 29499767 10.1186/s40478-018-0515-3 PMC5834839

[155] Dean DC 3rd, Hurley SA, Kecskemeti SR, O’Grady JP, Canda C, Davenport-Sis NJ, et al. Association of amyloid pathology with myelin alteration in preclinical Alzheimer disease. JAMA Neurol. 2017;74:41–49. 27842175 10.1001/jamaneurol.2016.3232 PMC5195903

[156] Huang Z, Jordan JD, Zhang Q. Myelin pathology in Alzheimer’s disease: potential therapeutic opportunities. Aging Dis. 2024;15:698–713. 37548935 10.14336/AD.2023.0628 PMC10917545

[157] Habes M, Pomponio R, Shou H, Doshi J, Mamourian E, Erus G, et al. The Brain Chart of Aging: Machine-learning analytics reveals links between brain aging, white matter disease, amyloid burden, and cognition in the iSTAGING consortium of 10,216 harmonized MR scans. Alzheimers Dement. 2021;17:89–102. 32920988 10.1002/alz.12178 PMC7923395

[158] Depp C, Sun T, Sasmita AO, Spieth L, Berghoff SA, Nazarenko T, et al. Myelin dysfunction drives amyloid-β deposition in models of Alzheimer’s disease. Nature. 2023;618:349–57. 37258678 10.1038/s41586-023-06120-6 PMC10247380

[159] Ishii A, Pathoulas JA, MoustafaFathy Omar O, Ge Y, Yao AY, Pantalena T, et al. Contribution of amyloid deposition from oligodendrocytes in a mouse model of Alzheimer’s disease. Mol Neurodegener. 2024;19:83. 39548583 10.1186/s13024-024-00759-z PMC11568619

[160] Polis B, Samson AO. Addressing the discrepancies between animal models and human alzheimer’s disease pathology: implications for translational research. J Alzheimers Dis. 2024;98:1199–218. 38517793 10.3233/JAD-240058

[161] Sasaguri H, Nilsson P, Hashimoto S, Nagata K, Saito T, De Strooper B, et al. APP mouse models for Alzheimer’s disease preclinical studies. Embo j. 2017;36:2473–87. 28768718 10.15252/embj.201797397 PMC5579350

[162] Pádua MS, Guil-Guerrero JL, Prates JAM, Lopes PA. Insights on the use of transgenic mice models in Alzheimer’s disease research. Int J Mol Sci. 2024;25:2805. 10.3390/ijms25052805 PMC10931675 38474051

[163] Oakley H, Cole SL, Logan S, Maus E, Shao P, Craft J, et al. Intraneuronal beta-amyloid aggregates, neurodegeneration, and neuron loss in transgenic mice with five familial Alzheimer’s disease mutations: potential factors in amyloid plaque formation. J Neurosci. 2006;26:10129–40. 17021169 10.1523/JNEUROSCI.1202-06.2006 PMC6674618

[164] Dong YX, Zhang HY, Li HY, Liu PH, Sui Y, Sun XH. Association between Alzheimer’s disease pathogenesis and early demyelination and oligodendrocyte dysfunction. Neural Regen Res. 2018;13:908–14. 29863022 10.4103/1673-5374.232486 PMC5998637

[165] Chacon-De-La-Rocha I, Fryatt G, Rivera AD, Verkhratsky A, Raineteau O, Gomez-Nicola D, et al. Accelerated Dystrophy and Decay of Oligodendrocyte Precursor Cells in the APP/PS1 Model of Alzheimer’s-Like Pathology. Front Cell Neurosci. 2020;14:575082. 33343301 10.3389/fncel.2020.575082 PMC7744306

[166] Oddo S, Caccamo A, Kitazawa M, Tseng BP, LaFerla FM. Amyloid deposition precedes tangle formation in a triple transgenic model of Alzheimer’s disease. Neurobiol Aging. 2003;24:1063–70. 14643377 10.1016/j.neurobiolaging.2003.08.012

[167] Tian S, Ye T, Cheng X. The behavioral, pathological and therapeutic features of the triple transgenic Alzheimer’s disease (3 × Tg-AD) mouse model strain. Exp Neurol. 2023;368:114505. 37597764 10.1016/j.expneurol.2023.114505

[168] Vanzulli I, Papanikolaou M, De-La-Rocha IC, Pieropan F, Rivera AD, Gomez-Nicola D, et al. Disruption of oligodendrocyte progenitor cells is an early sign of pathology in the triple transgenic mouse model of Alzheimer’s disease. Neurobiol Aging. 2020;94:130–9. 32619874 10.1016/j.neurobiolaging.2020.05.016 PMC7453384

[169] Richard BC, Kurdakova A, Baches S, Bayer TA, Weggen S, Wirths O. Gene dosage dependent aggravation of the neurological phenotype in the 5XFAD Mouse Model of Alzheimer’s disease. J Alzheimers Dis. 2015;45:1223–36. 25697701 10.3233/JAD-143120

[170] Chen ZY, Zhang Y. Animal models of Alzheimer’s disease: Applications, evaluation, and perspectives. Zool Res. 2022;43:1026–40. 36317468 10.24272/j.issn.2095-8137.2022.289 PMC9700500

[171] Russo ML, Ayala G, Neal D, Rogalsky AE, Ahmad S, Musial TF, et al. Alzheimer’s-linked axonal changes accompany elevated antidromic action potential failure rate in aged mice. Brain Res. 2024;1841:149083. 38866308 10.1016/j.brainres.2024.149083 PMC11323114

[172] Watamura N, Sato K, Saido TC. Mouse models of Alzheimer’s disease for preclinical research. Neurochem Int. 2022;158:105361. 35618239 10.1016/j.neuint.2022.105361

[173] Morrissey ZD, Gao J, Shetti A, Li W, Zhan L, Li W, et al. Temporal alterations in white matter in an app knock-in mouse model of Alzheimer’s disease. eNeuro. 2024;11:ENEURO.0496-23.2024. 10.1523/ENEURO.0496-23.2024 PMC10897532 38290851

[174] Chia SJ, Tan EK, Chao YX. Historical perspective: Models of Parkinson’s disease. Int J Mol Sci. 2020;21:2464. 10.3390/ijms21072464 PMC7177377 32252301

[175] Obeso JA, Rodriguez-Oroz MC, Goetz CG, Marin C, Kordower JH, Rodriguez M, et al. Missing pieces in the Parkinson’s disease puzzle. Nat Med. 2010;16:653–61. 20495568 10.1038/nm.2165

[176] Kikuchi T, Morizane A, Doi D, Magotani H, Onoe H, Hayashi T, et al. Human iPS cell-derived dopaminergic neurons function in a primate Parkinson’s disease model. Nature. 2017;548:592–6. 28858313 10.1038/nature23664

[177] Spillantini MG, Schmidt ML, Lee VM, Trojanowski JQ, Jakes R, Goedert M. Alpha-synuclein in Lewy bodies. Nature. 1997;388:839–40. 9278044 10.1038/42166

[178] Chen R, Gu X, Wang X. α-Synuclein in Parkinson’s disease and advances in detection. Clin Chim Acta. 2022;529:76–86. 35176268 10.1016/j.cca.2022.02.006

[179] Yang K, Wu Z, Long J, Li W, Wang X, Hu N, et al. White matter changes in Parkinson’s disease. NPJ Parkinsons Dis. 2023;9:150. 37907554 10.1038/s41531-023-00592-z PMC10618166

[180] Jiang YQ, Chen QZ, Yang Y, Zang CX, Ma JW, Wang JR, et al. White matter lesions contribute to motor and non-motor disorders in Parkinson’s disease: a critical review. Geroscience. 2025;47:591–609. 10.1007/s11357-024-01428-1 PMC11872850 39576561

[181] Dehestani M, Kozareva V, Blauwendraat C, Fraenkel E, Gasser T, Bansal V. Transcriptomic changes in oligodendrocytes and precursor cells predicts clinical outcomes of Parkinson’s disease. Mol Brain. 2024;17:56. 10.1186/s13041-024-01128-z PMC11323592 39138468

[182] Khan E, Hasan I, Haque ME. Parkinson’s disease: exploring different animal model systems. Int. J. Mol. Sci. 2023;24:9088. 10.3390/ijms24109088 PMC10219045 37240432

[183] van Steijn L, Verbeek FJ, Spaink HP, Merks RMH. Predicting metabolism from gene expression in an improved whole-genome metabolic network model of Danio rerio. Zebrafish. 2019;16:348–62. 31216234 10.1089/zeb.2018.1712 PMC6822484

[184] Zhang QS, Heng Y, Mou Z, Huang JY, Yuan YH, Chen NH. Reassessment of subacute MPTP-treated mice as animal model of Parkinson’s disease. Acta Pharm Sin. 2017;38:1317–28. 10.1038/aps.2017.49 PMC5630672 28649132

[185] Jackson-Lewis V, Przedborski S. Protocol for the MPTP mouse model of Parkinson’s disease. Nat Protoc. 2007;2:141–51. 17401348 10.1038/nprot.2006.342

[186] Jackson-Lewis V, Jakowec M, Burke RE, Przedborski S. Time course and morphology of dopaminergic neuronal death caused by the neurotoxin 1-methyl-4-phenyl-1,2,3,6-tetrahydropyridine. Neurodegeneration. 1995;4:257–69. 8581558 10.1016/1055-8330(95)90015-2

[187] Takagi S, Hayakawa N, Kimoto H, Kato H, Araki T. Damage to oligodendrocytes in the striatum after MPTP neurotoxicity in mice. J Neural Transm (Vienna). 2007;114:1553–7. 17676428 10.1007/s00702-007-0790-9

[188] Simola N, Morelli M, Carta AR. The 6-hydroxydopamine model of Parkinson’s disease. Neurotox Res. 2007;11:151–67. 17449457 10.1007/BF03033565

[189] Ungerstedt U, Arbuthnott GW. Quantitative recording of rotational behavior in rats after 6-hydroxy-dopamine lesions of the nigrostriatal dopamine system. Brain Res. 1970;24:485–93. 5494536 10.1016/0006-8993(70)90187-3

[190] Imamoto K. Effects of neonatal administrations of 6-OHDA on brain development. III. Myelin degeneration in the mesencephalic tract of the trigeminal nerve. Arch Histol Jpn. 1982;45:275–84. 7149918

[191] Worlitzer MM, Bunk EC, Hemmer K, Schwamborn JC. Anti-inflammatory treatment induced regenerative oligodendrogenesis in parkinsonian mice. Stem Cell Res Ther. 2012;3:33. 22892385 10.1186/scrt124 PMC3580471

[192] Schober A. Classic toxin-induced animal models of Parkinson’s disease: 6-OHDA and MPTP. Cell Tissue Res. 2004;318:215–24. 15503155 10.1007/s00441-004-0938-y

[193] Gasser T. Molecular pathogenesis of Parkinson disease: insights from genetic studies. Expert Rev Mol Med. 2009;11:e22. 19631006 10.1017/S1462399409001148

[194] Pena N, Richbourg T, Gonzalez-Hunt CP, Qi R, Wren P, Barlow C, et al. G2019S selective LRRK2 kinase inhibitor abrogates mitochondrial DNA damage. NPJ Parkinsons Dis. 2024;10:49. 38429321 10.1038/s41531-024-00660-y PMC10907374

[195] Singh V, Menard MA, Serrano GE, Beach TG, Zhao HT, Riley-DiPaolo A, et al. Cellular and subcellular localization of Rab10 and phospho-T73 Rab10 in the mouse and human brain. Acta Neuropathol Commun. 2023;11:201. 38110990 10.1186/s40478-023-01704-9 PMC10726543

[196] Zhang ZH, Zhao WQ, Ma FF, Zhang H, Xu XH. Rab10 disruption results in delayed OPC maturation. Cell Mol Neurobiol. 2017;37:1303–10. 28132130 10.1007/s10571-017-0465-5 PMC11482111

[197] Giasson BI, Duda JE, Quinn SM, Zhang B, Trojanowski JQ, Lee VM. Neuronal alpha-synucleinopathy with severe movement disorder in mice expressing A53T human alpha-synuclein. Neuron. 2002;34:521–33. 12062037 10.1016/s0896-6273(02)00682-7

[198] Lee MK, Stirling W, Xu Y, Xu X, Qui D, Mandir AS, et al. Human alpha-synuclein-harboring familial Parkinson’s disease-linked Ala-53 -> Thr mutation causes neurodegenerative disease with alpha-synuclein aggregation in transgenic mice. Proc Natl Acad Sci USA 2002, 99: 8968–73. 10.1073/pnas.132197599 PMC124407 12084935

[199] Konnova EA, Swanberg M. Animal Models of Parkinson’s Disease. In: Stoker TB, Greenland JC (eds). Parkinson’s Disease: Pathogenesis and Clinical Aspects. Codon Publications Copyright: The Authors.: Brisbane (AU), (2018).

[200] Yoon HH, Ye S, Lim S, Jo A, Lee H, Hong F, et al. CRISPR-Cas9 gene editing protects from the A53T-SNCA overexpression-induced pathology of Parkinson’s Disease in vivo. Crispr j. 2022;5:95–108. 35191750 10.1089/crispr.2021.0025

[201] Grigoletto J, Pukaß K, Gamliel A, Davidi D, Katz-Brull R, Richter-Landsberg C, et al. Higher levels of myelin phospholipids in brains of neuronal α-Synuclein transgenic mice precede myelin loss. Acta Neuropathol Commun. 2017;5:37. 28482862 10.1186/s40478-017-0439-3 PMC5421332

[202] Stefanova N, Reindl M, Neumann M, Haass C, Poewe W, Kahle PJ, et al. Oxidative stress in transgenic mice with oligodendroglial alpha-synuclein overexpression replicates the characteristic neuropathology of multiple system atrophy. Am J Pathol. 2005;166:869–76. 15743798 10.1016/s0002-9440(10)62307-3 PMC1602361

[203] Stemberger S, Poewe W, Wenning GK, Stefanova N. Targeted overexpression of human alpha-synuclein in oligodendroglia induces lesions linked to MSA-like progressive autonomic failure. Exp Neurol. 2010;224:459–64. 20493840 10.1016/j.expneurol.2010.05.008 PMC2913120

[204] Ubhi K, Rockenstein E, Mante M, Inglis C, Adame A, Patrick C, et al. Neurodegeneration in a transgenic mouse model of multiple system atrophy is associated with altered expression of oligodendroglial-derived neurotrophic factors. J Neurosci. 2010;30:6236–46. 20445049 10.1523/JNEUROSCI.0567-10.2010 PMC2896284

[205] Fellner L, Jellinger KA, Wenning GK, Stefanova N. Glial dysfunction in the pathogenesis of α-synucleinopathies: emerging concepts. Acta Neuropathol. 2011;121:675–93. 21562886 10.1007/s00401-011-0833-z PMC4730553

[206] Taylor JP, Brown RH Jr, Cleveland DW. Decoding ALS: from genes to mechanism. Nature. 2016;539:197–206. 27830784 10.1038/nature20413 PMC5585017

[207] Zhou T, Ahmad TK, Gozda K, Truong J, Kong J, Namaka M. Implications of white matter damage in amyotrophic lateral sclerosis (Review). Mol Med Rep. 2017;16:4379–92. 28791401 10.3892/mmr.2017.7186 PMC5646997

[208] Rafałowska J, Dziewulska D. White matter injury in amyotrophic lateral sclerosis (ALS). Folia Neuropathol. 1996;34:87–91. 8791897

[209] Müller HP, Abrahao A, Beaulieu C, Benatar M, Dionne A, Genge A, et al. Temporal and spatial progression of microstructural cerebral degeneration in ALS: A multicentre longitudinal diffusion tensor imaging study. Neuroimage Clin. 2024;43:103633. 38889523 10.1016/j.nicl.2024.103633 PMC11231599

[210] Cheng L, Tang X, Luo C, Liu D, Zhang Y, Zhang J. Fiber-specific white matter reductions in amyotrophic lateral sclerosis. Neuroimage Clin. 2020;28:102516. 33396003 10.1016/j.nicl.2020.102516 PMC7724379

[211] Chen HJ, Zhan C, Cai LM, Lin JH, Zhou MX, Zou ZY, et al. White matter microstructural impairments in amyotrophic lateral sclerosis: A mean apparent propagator MRI study. Neuroimage Clin. 2021;32:102863. 34700102 10.1016/j.nicl.2021.102863 PMC8551695

[212] Traiffort E, Morisset-Lopez S, Moussaed M, Zahaf A. Defective Oligodendroglial Lineage and Demyelination in Amyotrophic Lateral Sclerosis. Int J Mol Sci. 2021;22:3426. 10.3390/ijms22073426 PMC8036314 33810425

[213] Zhu Y, Burg T, Neyrinck K, Vervliet T, Nami F, Vervoort E, et al. Disruption of MAM integrity in mutant FUS oligodendroglial progenitors from hiPSCs. Acta Neuropathol. 2024;147:6. 38170217 10.1007/s00401-023-02666-x PMC10764485

[214] Rosen DR. Mutations in Cu/Zn superoxide dismutase gene are associated with familial amyotrophic lateral sclerosis. Nature. 1993;364:362. 8332197 10.1038/364362c0

[215] Li HF, Wu ZY. Genotype-phenotype correlations of amyotrophic lateral sclerosis. Transl Neurodegener. 2016;5:3. 26843957 10.1186/s40035-016-0050-8 PMC4738789

[216] De Giorgio F, Maduro C, Fisher EMC, Acevedo-Arozena A. Transgenic and physiological mouse models give insights into different aspects of amyotrophic lateral sclerosis. Dis Model Mech. 2019;12:dmm037424. 10.1242/dmm.037424 PMC6361152 30626575

[217] Renton AE, Majounie E, Waite A, Simón-Sánchez J, Rollinson S, Gibbs JR, et al. A hexanucleotide repeat expansion in C9ORF72 is the cause of chromosome 9p21-linked ALS-FTD. Neuron. 2011;72:257–68. 21944779 10.1016/j.neuron.2011.09.010 PMC3200438

[218] Vance C, Rogelj B, Hortobágyi T, De Vos KJ, Nishimura AL, Sreedharan J, et al. Mutations in FUS, an RNA processing protein, cause familial amyotrophic lateral sclerosis type 6. Science. 2009;323:1208–11. 19251628 10.1126/science.1165942 PMC4516382

[219] Kapeli K, Martinez FJ, Yeo GW. Genetic mutations in RNA-binding proteins and their roles in ALS. Hum Genet. 2017;136:1193–214. 28762175 10.1007/s00439-017-1830-7 PMC5602095

[220] Zhu L, Li S, Li XJ, Yin P. Pathological insights from amyotrophic lateral sclerosis animal models: comparisons, limitations, and challenges. Transl Neurodegener. 2023;12:46. 37730668 10.1186/s40035-023-00377-7 PMC10510301

[221] Guzman KM, Brink LE, Rodriguez-Bey G, Bodnar RJ, Kuang L, Xing B, et al. Conditional depletion of Fus in oligodendrocytes leads to motor hyperactivity and increased myelin deposition associated with Akt and cholesterol activation. Glia. 2020;68:2040–56. 32187401 10.1002/glia.23825 PMC7772959

[222] Wang J, Ho WY, Lim K, Feng J, Tucker-Kellogg G, Nave KA, et al. Cell-autonomous requirement of TDP-43, an ALS/FTD signature protein, for oligodendrocyte survival and myelination. Proc Natl Acad Sci USA. 2018;115:E10941–e10950. 30373824 10.1073/pnas.1809821115 PMC6243235

[223] Gurney ME, Pu H, Chiu AY, Dal Canto MC, Polchow CY, Alexander DD, et al. Motor neuron degeneration in mice that express a human Cu,Zn superoxide dismutase mutation. Science. 1994;264:1772–5. 8209258 10.1126/science.8209258

[224] Al-Chalabi A, van den Berg LH, Veldink J. Gene discovery in amyotrophic lateral sclerosis: implications for clinical management. Nat Rev Neurol. 2017;13:96–104. 27982040 10.1038/nrneurol.2016.182

[225] Bonfanti E, Bonifacino T, Raffaele S, Milanese M, Morgante E, Bonanno G, et al. Abnormal upregulation of GPR17 receptor contributes to Oligodendrocyte dysfunction in SOD1 G93A Mice. Int J Mol Sci. 2020;21:2395. 10.3390/ijms21072395 PMC7177925 32244295

[226] Sanchez G, Varaschin RK, Büeler H, Marcogliese PC, Park DS, Trudeau LE. Unaltered striatal dopamine release levels in young Parkin knockout, Pink1 knockout, DJ-1 knockout and LRRK2 R1441G transgenic mice. PLoS One. 2014;9:e94826. 24733019 10.1371/journal.pone.0094826 PMC3986353

[227] Zhang LJ, Tian DC, Yang L, Shi K, Liu Y, Wang Y, et al. White matter disease derived from vascular and demyelinating origins. Stroke Vasc Neurol. 2024;9:344–50. 37699727 10.1136/svn-2023-002791 PMC11420911

[228] Wang Y, Liu G, Hong D, Chen F, Ji X, Cao G. White matter injury in ischemic stroke. Prog Neurobiol. 2016;141:45–60. 27090751 10.1016/j.pneurobio.2016.04.005 PMC5677601

[229] Cheng CY, Lin JG, Tang NY, Kao ST, Hsieh CL. Electroacupuncture-like stimulation at the Baihui (GV20) and Dazhui (GV14) acupoints protects rats against subacute-phase cerebral ischemia-reperfusion injuries by reducing S100B-mediated neurotoxicity. PLoS One. 2014;9:e91426. 24626220 10.1371/journal.pone.0091426 PMC3953388

[230] Marin MA, Carmichael ST. Mechanisms of demyelination and remyelination in the young and aged brain following white matter stroke. Neurobiol Dis. 2019;126:5–12. 30031782 10.1016/j.nbd.2018.07.023

[231] Georgakis MK, Duering M, Wardlaw JM, Dichgans M. WMH and long-term outcomes in ischemic stroke: A systematic review and meta-analysis. Neurology. 2019;92:e1298–e1308. 30770431 10.1212/WNL.0000000000007142

[232] Sokolowski JD, Soldozy S, Sharifi KA, Norat P, Kearns KN, Liu L, et al. Preclinical models of middle cerebral artery occlusion: new imaging approaches to a classic technique. Front Neurol. 2023;14:1170675. 37409019 10.3389/fneur.2023.1170675 PMC10318149

[233] Tachibana M, Ago T, Wakisaka Y, Kuroda J, Shijo M, Yoshikawa Y, et al. Early Reperfusion After Brain Ischemia Has Beneficial Effects Beyond Rescuing Neurons. Stroke. 2017;48:2222–30. 28626056 10.1161/STROKEAHA.117.016689

[234] Jia J, Zheng L, Ye L, Chen J, Shu S, Xu S, et al. CD11c(+) microglia promote white matter repair after ischemic stroke. Cell Death Dis. 2023;14:156. 36828819 10.1038/s41419-023-05689-0 PMC9958101

[235] Longa EZ, Weinstein PR, Carlson S, Cummins R. Reversible middle cerebral artery occlusion without craniectomy in rats. Stroke. 1989;20:84–91. 2643202 10.1161/01.str.20.1.84

[236] Yang X, Wu Q, Zhang L, Feng L. Inhibition of Histone Deacetylase 3 (HDAC3) mediates ischemic preconditioning and protects Cortical neurons against ischemia in rats. Front Mol Neurosci. 2016;9:131. 27965534 10.3389/fnmol.2016.00131 PMC5124709

[237] Zhou X, Qiao B. Inhibition of HDAC3 and ATXN3 by miR-25 prevents neuronal loss and ameliorates neurological recovery in cerebral stroke experimental rats. J Physiol Biochem. 2022;78:139–49. 35025075 10.1007/s13105-021-00848-3

[238] Kemps H, Dessy C, Dumas L, Sonveaux P, Alders L, Van Broeckhoven J, et al. Extremely low frequency electromagnetic stimulation reduces ischemic stroke volume by improving cerebral collateral blood flow. J Cereb Blood Flow Metab. 2022;42:979–96. 35209740 10.1177/0271678X221084410 PMC9125494

[239] Cheng YJ, Wang F, Feng J, Yu B, Wang B, Gao Q, et al. Prolonged myelin deficits contribute to neuron loss and functional impairments after ischaemic stroke. Brain. 2024;147:1294–311. 38289861 10.1093/brain/awae029

[240] Tuo QZ, Zou JJ, Lei P. Rodent models of vascular cognitive impairment. J Mol Neurosci. 2021;71:1–12. 10.1007/s12031-020-01733-2 33107013

[241] Wahul AB, Joshi PC, Kumar A, Chakravarty S. Transient global cerebral ischemia differentially affects cortex, striatum and hippocampus in Bilateral Common Carotid Arterial occlusion (BCCAo) mouse model. J Chem Neuroanat. 2018;92:1–15. 29702163 10.1016/j.jchemneu.2018.04.006

[242] Choi BR, Kim DH, Back DB, Kang CH, Moon WJ, Han JS, et al. Characterization of white matter injury in a rat model of chronic cerebral hypoperfusion. Stroke. 2016;47:542–7. 26670084 10.1161/STROKEAHA.115.011679

[243] Wang J, Yang C, Wang H, Li D, Li T, Sun Y, et al. A new rat model of chronic cerebral hypoperfusion resulting in early-stage vascular cognitive impairment. Front Aging Neurosci. 2020;12:86. 32351379 10.3389/fnagi.2020.00086 PMC7174718

[244] Shibata M, Ohtani R, Ihara M, Tomimoto H. White matter lesions and glial activation in a novel mouse model of chronic cerebral hypoperfusion. Stroke. 2004;35:2598–603. 15472111 10.1161/01.STR.0000143725.19053.60

[245] Ishikawa H, Shindo A, Mizutani A, Tomimoto H, Lo EH, Arai K. A brief overview of a mouse model of cerebral hypoperfusion by bilateral carotid artery stenosis. J Cereb Blood Flow Metab. 2023;43:18–36. 36883344 10.1177/0271678X231154597 PMC10638994

[246] Kakae M, Kawashita A, Onogi H, Nakagawa T, Shirakawa H. Bilateral common carotid artery stenosis in mice: a model of chronic cerebral hypoperfusion-induced vascular cognitive impairment. Bio Protoc. 2024;14:e5022. 10.21769/BioProtoc.5022 PMC11238111 39007157

[247] Xie Y, Zhang X, Xu P, Zhao N, Zhao Y, Li Y, et al. Aberrant oligodendroglial LDL receptor orchestrates demyelination in chronic cerebral ischemia. J Clin Invest. 2021;131:e128114. 10.1172/JCI128114 PMC7773390 33141760

[248] Back DB, Choi BR, Han JS, Kwon KJ, Choi DH, Shin CY, et al. Characterization of Tauopathy in a rat model of post-stroke dementia combining acute infarct and chronic cerebral hypoperfusion. Int J Mol Sci. 2020;21:6929. 10.3390/ijms21186929 PMC7555397 32967251

[249] Westphal LP, Schweizer J, Fluri F, De Marchis GM, Christ-Crain M, Luft AR, et al. C-Terminal-Pro-Endothelin-1 Adds Incremental Prognostic Value for Risk Stratification After Ischemic Stroke. Front Neurol. 2020;11:629151. 33584523 10.3389/fneur.2020.629151 PMC7873365

[250] Frost SB, Barbay S, Mumert ML, Stowe AM, Nudo RJ. An animal model of capsular infarct: endothelin-1 injections in the rat. Behav Brain Res. 2006;169:206–11. 16497394 10.1016/j.bbr.2006.01.014

[251] Sommer CJ. Ischemic stroke: experimental models and reality. Acta Neuropathol. 2017;133:245–61. 28064357 10.1007/s00401-017-1667-0 PMC5250659

[252] Iijima K, Kurachi M, Shibasaki K, Naruse M, Puentes S, Imai H, et al. Transplanted microvascular endothelial cells promote oligodendrocyte precursor cell survival in ischemic demyelinating lesions. J Neurochem. 2015;135:539–50. 26212499 10.1111/jnc.13262

[253] Ohashi K, Shibasaki K, Nakazawa H, Kunimasa R, Nagayasu K, Shirakawa H, et al. Transient Receptor Potential Melastatin 3 is functionally expressed in Oligodendrocyte precursor cells and is upregulated in ischemic demyelinated lesions. Biol Pharm Bull. 2021;44:181–7. 33518671 10.1248/bpb.b20-00510

[254] Lui M, Gouveia A, Lagace D, Wang J. Combination of Endothelin-1 (ET-1) and L-NAME to induce murine focal cortical stroke with persistent sensorimotor deficits. Methods Mol Biol. 2022;2515:75–87. 35776346 10.1007/978-1-0716-2409-8_5

[255] An SJ, Kim TJ, Yoon BW. Epidemiology, risk factors, and clinical features of intracerebral hemorrhage: an update. J Stroke. 2017;19:3–10. 28178408 10.5853/jos.2016.00864 PMC5307940

[256] Morotti A, Goldstein JN. Diagnosis and management of acute intracerebral hemorrhage. Emerg Med Clin North Am. 2016;34:883–99. 27741993 10.1016/j.emc.2016.06.010 PMC5089075

[257] Greenberg SM, Ziai WC, Cordonnier C, Dowlatshahi D, Francis B, Goldstein JN, et al. 2022 Guideline for the Management of Patients With Spontaneous Intracerebral Hemorrhage: A Guideline From the American Heart Association/American Stroke Association. Stroke. 2022;53:e282–e361. 35579034 10.1161/STR.0000000000000407

[258] Aronowski J, Zhao X. Molecular pathophysiology of cerebral hemorrhage: secondary brain injury. Stroke. 2011;42:1781–6. 21527759 10.1161/STROKEAHA.110.596718 PMC3123894

[259] Wilkinson CM, Brar PS, Balay CJ, Colbourne F. Glibenclamide, a Sur1-Trpm4 antagonist, does not improve outcome after collagenase-induced intracerebral hemorrhage. PLoS One. 2019;14:e0215952. 31042750 10.1371/journal.pone.0215952 PMC6494051

[260] Fang J, Song F, Chang C, Yao M. Intracerebral hemorrhage models and behavioral tests in rodents. Neuroscience. 2023;513:1–13. 36690062 10.1016/j.neuroscience.2023.01.011

[261] Yang GY, Betz AL, Chenevert TL, Brunberg JA, Hoff JT. Experimental intracerebral hemorrhage: relationship between brain edema, blood flow, and blood-brain barrier permeability in rats. J Neurosurg. 1994;81:93–102. 8207532 10.3171/jns.1994.81.1.0093

[262] Deng R, Wang W, Xu X, Ding J, Wang J, Yang S, et al. Loss of MIC60 aggravates neuronal death by inducing mitochondrial dysfunction in a rat model of intracerebral hemorrhage. Mol Neurobiol. 2021;58:4999–5013. 34232477 10.1007/s12035-021-02468-w

[263] Shen D, Wu W, Liu J, Lan T, Xiao Z, Gai K, et al. Ferroptosis in oligodendrocyte progenitor cells mediates white matter injury after hemorrhagic stroke. Cell Death Dis. 2022;13:259. 35318305 10.1038/s41419-022-04712-0 PMC8941078

[264] Qu J, Zong HF, Shan Y, Zhang SC, Guan WP, Yang Y, et al. Piezo1 suppression reduces demyelination after intracerebral hemorrhage. Neural Regen Res. 2023;18:1750–6. 36751801 10.4103/1673-5374.361531 PMC10154511

[265] Deinsberger W, Vogel J, Kuschinsky W, Auer LM, Böker DK. Experimental intracerebral hemorrhage: description of a double injection model in rats. Neurol Res. 1996;18:475–7. 8916066 10.1080/01616412.1996.11740456

[266] Ma B, Zhang J. Nimodipine treatment to assess a modified mouse model of intracerebral hemorrhage. Brain Res. 2006;1078:182–8. 16492378 10.1016/j.brainres.2006.01.045

[267] Karki K, Knight RA, Han Y, Yang D, Zhang J, Ledbetter KA, et al. Simvastatin and atorvastatin improve neurological outcome after experimental intracerebral hemorrhage. Stroke. 2009;40:3384–9. 19644071 10.1161/STROKEAHA.108.544395 PMC2758425

[268] Lei B, Sheng H, Wang H, Lascola CD, Warner DS, Laskowitz DT, et al. Intrastriatal injection of autologous blood or clostridial collagenase as murine models of intracerebral hemorrhage. J Vis Exp. 2014;89:51439. 10.3791/51439 PMC4211435 25046028

[269] Rosenberg GA, Mun-Bryce S, Wesley M, Kornfeld M. Collagenase-induced intracerebral hemorrhage in rats. Stroke. 1990;21:801–7. 2160142 10.1161/01.str.21.5.801

[270] Rosenberg GA, Estrada E, Kelley RO, Kornfeld M. Bacterial collagenase disrupts extracellular matrix and opens blood-brain barrier in rat. Neurosci Lett. 1993;160:117–9. 8247322 10.1016/0304-3940(93)90927-d

[271] Joseph MJ, Caliaperumal J, Schlichter LC. After intracerebral hemorrhage, oligodendrocyte precursors proliferate and differentiate inside white-matter tracts in the rat striatum. Transl Stroke Res. 2016;7:192–208. 26743212 10.1007/s12975-015-0445-3 PMC4873533

[272] James ML, Warner DS, Laskowitz DT. Preclinical models of intracerebral hemorrhage: a translational perspective. Neurocrit Care. 2008;9:139–52. 18058257 10.1007/s12028-007-9030-2

[273] Andaluz N, Zuccarello M, Wagner KR. Experimental animal models of intracerebral hemorrhage. Neurosurg Clin N Am. 2002;13:385–93. 12486927 10.1016/s1042-3680(02)00006-2

[274] Rosenberg GA, Navratil M. Metalloproteinase inhibition blocks edema in intracerebral hemorrhage in the rat. Neurology. 1997;48:921–6. 9109878 10.1212/wnl.48.4.921

[275] Bailey EL, Smith C, Sudlow CL, Wardlaw JM. Is the spontaneously hypertensive stroke prone rat a pertinent model of sub cortical ischemic stroke? A systematic review. Int J Stroke. 2011;6:434–44. 21951409 10.1111/j.1747-4949.2011.00659.x

[276] Okamoto K, Yamamoto K, Morita N, Ohta Y, Chikugo T, Higashizawa T, et al. Establishment and use of the M strain of stroke-prone spontaneously hypertensive rat. J Hypertens Suppl. 1986;4:S21–24. 3465899

[277] Yang Y, Kimura-Ohba S, Thompson J, Rosenberg GA. Rodent models of vascular cognitive impairment. Transl Stroke Res. 2016;7:407–14. 27498679 10.1007/s12975-016-0486-2 PMC5016244

[278] Rosenberg GA, Wallin A, Wardlaw JM, Markus HS, Montaner J, Wolfson L, et al. Consensus statement for diagnosis of subcortical small vessel disease. J Cereb Blood Flow Metab. 2016;36:6–25. 26198175 10.1038/jcbfm.2015.172 PMC4758552

[279] Ishizuka T, Niwa A, Tabuchi M, Nagatani Y, Ooshima K, Higashino H. Involvement of thromboxane A2 receptor in the cerebrovascular damage of salt-loaded, stroke-prone rats. J Hypertens. 2007;25:861–70. 17351380 10.1097/HJH.0b013e3280464dc8

[280] Zhang Y, Sheikh AM, Tabassum S, Iwasa K, Shibly AZ, Zhou X, et al. Effect of high-fat diet on cerebral pathological changes of cerebral small vessel disease in SHR/SP rats. Geroscience. 2024;46:3779–3800. 38319539 10.1007/s11357-024-01074-7 PMC11226591

[281] Sironi L, Guerrini U, Tremoli E, Miller I, Gelosa P, Lascialfari A, et al. Analysis of pathological events at the onset of brain damage in stroke-prone rats: a proteomics and magnetic resonance imaging approach. J Neurosci Res. 2004;78:115–22. 15372505 10.1002/jnr.20219

[282] Synnes AR, Anson S, Arkesteijn A, Butt A, Grunau RE, Rogers M, et al. School entry age outcomes for infants with birth weight ≤800 grams. J Pediatr. 2010;157:989–994.e981. 20674931 10.1016/j.jpeds.2010.06.016

[283] Perlman JM. White matter injury in the preterm infant: an important determination of abnormal neurodevelopment outcome. Early Hum Dev. 1998;53:99–120. 10195704 10.1016/s0378-3782(98)00037-1

[284] Guillot M, Miller SP. The dimensions of white matter injury in preterm neonates. Semin Perinatol. 2021;45:151469. 34456064 10.1016/j.semperi.2021.151469

[285] Rice JE 3rd, Vannucci RC, Brierley JB. The influence of immaturity on hypoxic-ischemic brain damage in the rat. Ann Neurol. 1981;9:131–41. 7235629 10.1002/ana.410090206

[286] Vannucci RC, Connor JR, Mauger DT, Palmer C, Smith MB, Towfighi J, et al. Rat model of perinatal hypoxic-ischemic brain damage. J Neurosci Res. 1999;55:158–63. 9972818 10.1002/(SICI)1097-4547(19990115)55:2<158::AID-JNR3>3.0.CO;2-1

[287] Vannucci SJ, Back SA. The Vannucci model of hypoxic-ischemic injury in the neonatal rodent: 40 years later. Dev Neurosci. 2022;44:186–93. 35263745 10.1159/000523990

[288] Stadlin A, James A, Fiscus R, Wong YF, Rogers M, Haines C. Development of a postnatal 3-day-old rat model of mild hypoxic-ischemic brain injury. Brain Res. 2003;993:101–10. 14642835 10.1016/j.brainres.2003.08.058

[289] Gao T, Qian T, Wang T, Su Y, Qiu H, Tang W, et al. T0901317, a liver X receptor agonist, ameliorates perinatal white matter injury induced by ischemia and hypoxia in neonatal rats. Neurosci Lett. 2023;793:136994. 36460235 10.1016/j.neulet.2022.136994

[290] Deng YP, Sun Y, Hu L, Li ZH, Xu QM, Pei YL, et al. Chondroitin sulfate proteoglycans impede myelination by oligodendrocytes after perinatal white matter injury. Exp Neurol. 2015;269:213–23. 25862289 10.1016/j.expneurol.2015.03.026

[291] Renz P, Schoeberlein A, Haesler V, Maragkou T, Surbek D, Brosius Lutz A. A novel murine multi-hit model of perinatal acute diffuse white matter injury recapitulates major features of human disease. Biomedicines. 2022;10:2810. 10.3390/biomedicines10112810 PMC9687579 36359331

[292] Schang AL, Van Steenwinckel J, Ioannidou ZS, Lipecki J, Rich-Griffin C, Woolley-Allen K, et al. Epigenetic priming of immune/inflammatory pathways activation and abnormal activity of cell cycle pathway in a perinatal model of white matter injury. Cell Death Dis. 2022;13:1038. 36513635 10.1038/s41419-022-05483-4 PMC9748018

[293] Krishnan ML, Van Steenwinckel J, Schang AL, Yan J, Arnadottir J, Le Charpentier T, et al. Integrative genomics of microglia implicates DLG4 (PSD95) in the white matter development of preterm infants. Nat Commun. 2017;8:428. 28874660 10.1038/s41467-017-00422-w PMC5585205

[294] Favrais G, van de Looij Y, Fleiss B, Ramanantsoa N, Bonnin P, Stoltenburg-Didinger G, et al. Systemic inflammation disrupts the developmental program of white matter. Ann Neurol. 2011;70:550–65. 21796662 10.1002/ana.22489

[295] Xiong Y, Mahmood A, Chopp M. Animal models of traumatic brain injury. Nat Rev Neurosci. 2013;14:128–42. 23329160 10.1038/nrn3407 PMC3951995

[296] Rocchi A, Sariyer IK, Berger JR. Revisiting JC virus and progressive multifocal leukoencephalopathy. J Neurovirol. 2023;29:524–37. 37659983 10.1007/s13365-023-01164-w

[297] Xu X, Gao W, Cheng S, Yin D, Li F, Wu Y, et al. Anti-inflammatory and immunomodulatory mechanisms of atorvastatin in a murine model of traumatic brain injury. J Neuroinflammation. 2017;14:167. 28835272 10.1186/s12974-017-0934-2 PMC5569493

[298] Kaur P, Sharma S. Recent advances in pathophysiology of traumatic brain injury. Curr Neuropharmacol. 2018;16:1224–38. 28606040 10.2174/1570159X15666170613083606 PMC6142406

[299] Wang G, Zhang J, Hu X, Zhang L, Mao L, Jiang X, et al. Microglia/macrophage polarization dynamics in white matter after traumatic brain injury. J Cereb Blood Flow Metab. 2013;33:1864–74. 23942366 10.1038/jcbfm.2013.146 PMC3851898

[300] Atkins CM, Cepero ML, Kang Y, Liebl DJ, Dietrich WD. Effects of early rolipram treatment on histopathological outcome after controlled cortical impact injury in mice. Neurosci Lett. 2013;532:1–6. 23103712 10.1016/j.neulet.2012.10.019 PMC3527646

[301] Xu SY, Liu M, Gao Y, Cao Y, Bao JG, Lin YY, et al. Acute histopathological responses and long-term behavioral outcomes in mice with graded controlled cortical impact injury. Neural Regen Res. 2019;14:997–1003. 30762011 10.4103/1673-5374.250579 PMC6404507

[302] Siebold L, Obenaus A, Goyal R. Criteria to define mild, moderate, and severe traumatic brain injury in the mouse controlled cortical impact model. Exp Neurol. 2018;310:48–57. 30017882 10.1016/j.expneurol.2018.07.004

[303] Meng S, Cao H, Huang Y, Shi Z, Li J, Wang Y, et al. ASK1-K716R reduces neuroinflammation and white matter injury via preserving blood-brain barrier integrity after traumatic brain injury. J Neuroinflammation. 2023;20:244. 37875988 10.1186/s12974-023-02923-6 PMC10594934

[304] Zhou W, Liang Y, Liao X, Tong L, Du W, Fu W, et al. ISRIB improves white matter injury following TBI by inhibiting NCOA4-mediated ferritinophagy. Neurochem Int. 2024;177:105744. 38663454 10.1016/j.neuint.2024.105744

[305] Feeney DM, Boyeson MG, Linn RT, Murray HM, Dail WG. Responses to cortical injury: I. Methodology and local effects of contusions in the rat. Brain Res. 1981;211:67–77. 7225844 10.1016/0006-8993(81)90067-6

[306] Goldstein LE, Fisher AM, Tagge CA, Zhang XL, Velisek L, Sullivan JA, et al. Chronic traumatic encephalopathy in blast-exposed military veterans and a blast neurotrauma mouse model. Sci Transl Med. 2012;4:134ra160. 10.1126/scitranslmed.3003716 PMC3739428 22593173

[307] Marmarou A, Foda MA, van den Brink W, Campbell J, Kita H, Demetriadou K. A new model of diffuse brain injury in rats. Part I: Pathophysiology and biomechanics. J Neurosurg. 1994;80:291–300. 8283269 10.3171/jns.1994.80.2.0291

[308] Foda MA, Marmarou A. A new model of diffuse brain injury in rats. Part II: Morphological characterization. J Neurosurg. 1994;80:301–13. 8283270 10.3171/jns.1994.80.2.0301

[309] Kilbourne M, Kuehn R, Tosun C, Caridi J, Keledjian K, Bochicchio G, et al. Novel model of frontal impact closed head injury in the rat. J Neurotrauma. 2009;26:2233–43. 19929375 10.1089/neu.2009.0968 PMC2824220

[310] Hicks R, Soares H, Smith D, McIntosh T. Temporal and spatial characterization of neuronal injury following lateral fluid-percussion brain injury in the rat. Acta Neuropathol. 1996;91:236–46. 8834535 10.1007/s004010050421

[311] Alder J, Fujioka W, Lifshitz J, Crockett DP, Thakker-Varia S. Lateral fluid percussion: model of traumatic brain injury in mice. J Vis Exp. 2011;54:3063. 10.3791/3063 PMC3217637 21876530

[312] Adams AA, Wood TL, Kim HA. Mature and Myelinating Oligodendrocytes Are Specifically Vulnerable to Mild Fluid Percussion Injury in Mice. Neurotrauma Rep. 2023;4:433–46. 37435356 10.1089/neur.2023.0037 PMC10331160

[313] Flygt J, Djupsjö A, Lenne F, Marklund N. Myelin loss and oligodendrocyte pathology in white matter tracts following traumatic brain injury in the rat. Eur J Neurosci. 2013;38:2153–65. 23458840 10.1111/ejn.12179

[314] Cernak I, Noble-Haeusslein LJ. Traumatic brain injury: an overview of pathobiology with emphasis on military populations. J Cereb Blood Flow Metab. 2010;30:255–66. 19809467 10.1038/jcbfm.2009.203 PMC2855235

[315] Garman RH, Jenkins LW, Switzer RC 3rd, Bauman RA, Tong LC, Swauger PV, et al. Blast exposure in rats with body shielding is characterized primarily by diffuse axonal injury. J Neurotrauma. 2011;28:947–59. 21449683 10.1089/neu.2010.1540

[316] Sawyer TW, Ritzel DV, Wang Y, Josey T, Villanueva M, Nelson P, et al. Primary blast causes delayed effects without cell death in shell-encased brain cell aggregates. J Neurotrauma. 2018;35:174–86. 28726571 10.1089/neu.2016.4961

[317] Zheng W, Chen Q, Chen X, Wan L, Qin W, Qi Z, et al. Brain white matter impairment in patients with spinal cord injury. Neural Plast. 2017;2017:4671607. 28255458 10.1155/2017/4671607 PMC5309430

[318] Almad A, Sahinkaya FR, McTigue DM. Oligodendrocyte fate after spinal cord injury. Neurotherapeutics. 2011;8:262–73. 21404073 10.1007/s13311-011-0033-5 PMC3101831

[319] Profyris C, Cheema SS, Zang D, Azari MF, Boyle K, Petratos S. Degenerative and regenerative mechanisms governing spinal cord injury. Neurobiol Dis. 2004;15:415–36. 15056450 10.1016/j.nbd.2003.11.015

[320] Li N, Leung GK. Oligodendrocyte precursor cells in spinal cord injury: a review and update. Biomed Res Int. 2015;2015:235195. 26491661 10.1155/2015/235195 PMC4600489

[321] Duncan GJ, Manesh SB, Hilton BJ, Assinck P, Plemel JR, Tetzlaff W. The fate and function of oligodendrocyte progenitor cells after traumatic spinal cord injury. Glia. 2020;68:227–45. 31433109 10.1002/glia.23706

[322] Anjum A, Yazid MD, Fauzi Daud M, Idris J, Ng AMH, Selvi Naicker A, et al. Spinal cord injury: pathophysiology, multimolecular interactions, and underlying recovery mechanisms. Int J Mol Sci. 2020;21:7533. 10.3390/ijms21207533 PMC7589539 33066029

[323] Mattucci S, Speidel J, Liu J, Kwon BK, Tetzlaff W, Oxland TR. Basic biomechanics of spinal cord injury - How injuries happen in people and how animal models have informed our understanding. Clin Biomech. 2019;64:58–68. 10.1016/j.clinbiomech.2018.03.020 29685426

[324] Sharif-Alhoseini M, Khormali M, Rezaei M, Safdarian M, Hajighadery A, Khalatbari MM, et al. Animal models of spinal cord injury: a systematic review. Spinal Cord. 2017;55:714–21. 28117332 10.1038/sc.2016.187

[325] Kwon BK, Okon EB, Tsai E, Beattie MS, Bresnahan JC, Magnuson DK, et al. A grading system to evaluate objectively the strength of pre-clinical data of acute neuroprotective therapies for clinical translation in spinal cord injury. J Neurotrauma. 2011;28:1525–43. 20507235 10.1089/neu.2010.1296 PMC3143387

[326] Khuyagbaatar B, Kim K, Kim YH. Conversion Equation between the drop height in the New York university impactor and the impact force in the infinite horizon impactor in the contusion spinal cord injury model. J Neurotrauma. 2015;32:1987–93. 26058442 10.1089/neu.2015.3875

[327] Rong W, Pan YW, Cai X, Song F, Zhao Z, Xiao SH, et al. The mechanism of Naringin-enhanced remyelination after spinal cord injury. Neural Regen Res. 2017;12:470–7. 28469664 10.4103/1673-5374.202923 PMC5399727

[328] Li ZW, Li JJ, Wang L, Zhang JP, Wu JJ, Mao XQ, et al. Epidermal growth factor receptor inhibitor ameliorates excessive astrogliosis and improves the regeneration microenvironment and functional recovery in adult rats following spinal cord injury. J Neuroinflammation. 2014;11:71. 24708754 10.1186/1742-2094-11-71 PMC4030311

[329] Jarragh A, Shuaib A, Al-Khaledi G, Alotaibi F, Al-Sabah S, Masocha W. A custom-made weight-drop impactor to produce consistent spinal cord injury outcomes in a rat model. Transl Neurosci. 2023;14:20220287. 37250141 10.1515/tnsci-2022-0287 PMC10224629

[330] Stokes BT. Experimental spinal cord injury: a dynamic and verifiable injury device. J Neurotrauma. 1992;9:129–31. discussion 131-124. 1404426 10.1089/neu.1992.9.129

[331] Marcol W, Slusarczyk W, Gzik M, Larysz-Brysz M, Bobrowski M, Grynkiewicz-Bylina B, et al. Air gun impactor-a novel model of graded white matter spinal cord injury in rodents. J Reconstr Microsurg. 2012;28:561–8. 22711195 10.1055/s-0032-1315779

[332] Kondiles BR, Wei H, Chaboub LS, Horner PJ, Wu JQ, Perlmutter SI. Transcriptome of rat subcortical white matter and spinal cord after spinal injury and cortical stimulation. Sci Data. 2021;8:175. 34267212 10.1038/s41597-021-00953-4 PMC8282877

[333] Feigenbaum L, Khalili K, Major E, Khoury G. Regulation of the host range of human papovavirus JCV. Proc Natl Acad Sci USA. 1987;84:3695–8. 3035549 10.1073/pnas.84.11.3695 PMC304942

[334] Walker DL, Padgett BL, ZuRhein GM, Albert AE, Marsh RF. Human papovavirus (JC): induction of brain tumors in hamsters. Science. 1973;181:674–6. 4353360 10.1126/science.181.4100.674

[335] London WT, Houff SA, Madden DL, Fuccillo DA, Gravell M, Wallen WC, et al. Brain tumors in owl monkeys inoculated with a human polyomavirus (JC virus). Science. 1978;201:1246–9. 211583 10.1126/science.211583

[336] London WT, Houff SA, McKeever PE, Wallen WC, Sever JL, Padgett BL, et al. Viral-induced astrocytomas in squirrel monkeys. Prog Clin Biol Res. 1983;105:227–37. 6304760

[337] Krynska B, Otte J, Franks R, Khalili K, Croul S. Human ubiquitous JCV(CY) T-antigen gene induces brain tumors in experimental animals. Oncogene. 1999;18:39–46. 9926918 10.1038/sj.onc.1202278

[338] Windrem MS, Nunes MC, Rashbaum WK, Schwartz TH, Goodman RA, McKhann G 2nd, et al. Fetal and adult human oligodendrocyte progenitor cell isolates myelinate the congenitally dysmyelinated brain. Nat Med. 2004;10:93–97. 14702638 10.1038/nm974

[339] Kondo Y, Windrem MS, Zou L, Chandler-Militello D, Schanz SJ, Auvergne RM, et al. Human glial chimeric mice reveal astrocytic dependence of JC virus infection. J Clin Invest. 2014;124:5323–36. 25401469 10.1172/JCI76629 PMC4348956

[340] White MK, Gordon J, Berger JR, Khalili K. Animal models for progressive multifocal Leukoencephalopathy. J Cell Physiol. 2015;230:2869–74. 26041694 10.1002/jcp.25047 PMC4549183

[341] Shwetank, Frost EL, Mockus TE, Ren HM, Toprak M, Lauver MD, et al. PD-1 dynamically regulates inflammation and development of brain-resident memory CD8 T cells during persistent viral Encephalitis. Front Immunol. 2019;10:783. 31105690 10.3389/fimmu.2019.00783 PMC6499176

[342] Ayers KN, Carey SN, Lukacher AE. Understanding polyomavirus CNS disease - a perspective from mouse models. Febs j. 2022;289:5744–61. 34145975 10.1111/febs.16083 PMC8826653

[343] Barreras P, Pamies D, Monaco MC, Muñoz LS, Zhong X, Major EO, et al. A human-derived 3D brain organoid model to study JC virus infection. J Neurovirol. 2022;28:17–26. 35239145 10.1007/s13365-022-01062-7 PMC8892818

[344] Hol EM, Dykstra W, Chevalier J, Cuadrado E, Bugiani M, Aronica E, et al. Neuroglia in leukodystrophies. Handb Clin Neurol. 2025;210:159–75. 40148043 10.1016/B978-0-443-19102-2.00032-6

[345] Moser HW. Adrenoleukodystrophy: phenotype, genetics, pathogenesis and therapy. Brain. 1997;120:1485–508. 9278636 10.1093/brain/120.8.1485

[346] Sosunov A, Olabarria M, Goldman JE. Alexander disease: an astrocytopathy that produces a leukodystrophy. Brain Pathol. 2018;28:388–98. 29740945 10.1111/bpa.12601 PMC8028392

[347] Tung H, Chou CC, Chen HM, Chen YM, Wu YY, Chai JW, et al. White Matter Hyperintensities and cognitive functions in people with the R544C variant of the NOTCH3 gene without stroke or dementia. Neurology. 2024;103:e209941. 39374470 10.1212/WNL.0000000000209941

[348] Moser AB, Jones RO, Hubbard WC, Tortorelli S, Orsini JJ, Caggana M, et al. Newborn Screening for X-Linked Adrenoleukodystrophy. Int J Neonatal Screen. 2016;2:15. 10.3390/ijns2040015 PMC6715319 31467997

[349] Lauer A, Da X, Hansen MB, Boulouis G, Ou Y, Cai X, et al. ABCD1 dysfunction alters white matter microvascular perfusion. Brain. 2017;140:3139–52. 29136088 10.1093/brain/awx262 PMC5841142

[350] Lu JF, Lawler AM, Watkins PA, Powers JM, Moser AB, Moser HW, et al. A mouse model for X-linked adrenoleukodystrophy. Proc Natl Acad Sci USA. 1997;94:9366–71. 9256488 10.1073/pnas.94.17.9366 PMC23196

[351] Forss-Petter S, Werner H, Berger J, Lassmann H, Molzer B, Schwab MH, et al. Targeted inactivation of the X-linked adrenoleukodystrophy gene in mice. J Neurosci Res. 1997;50:829–43. 9418970 10.1002/(SICI)1097-4547(19971201)50:5<829::AID-JNR19>3.0.CO;2-W

[352] Kobayashi T, Shinnoh N, Kondo A, Yamada T. Adrenoleukodystrophy protein-deficient mice represent abnormality of very long chain fatty acid metabolism. Biochem Biophys Res Commun. 1997;232:631–6. 9126326 10.1006/bbrc.1997.6340

[353] Pujol A, Hindelang C, Callizot N, Bartsch U, Schachner M, Mandel JL. Late onset neurological phenotype of the X-ALD gene inactivation in mice: a mouse model for adrenomyeloneuropathy. Hum Mol Genet. 2002;11:499–505. 11875044 10.1093/hmg/11.5.499

[354] Rutherford HA, Hamilton N. Animal models of leukodystrophy: a new perspective for the development of therapies. FEBS J. 2019;286:4176–91. 31520449 10.1111/febs.15060

[355] Martinović K, Bauer J, Kunze M, Berger J, Forss-Petter S. Abcd1 deficiency accelerates cuprizone-induced oligodendrocyte loss and axonopathy in a demyelinating mouse model of X-linked adrenoleukodystrophy. Acta Neuropathol Commun. 2023;11:98. 37331971 10.1186/s40478-023-01595-w PMC10276915

[356] Schlüter A, Sandoval J, Fourcade S, Díaz-Lagares A, Ruiz M, Casaccia P, et al. Epigenomic signature of adrenoleukodystrophy predicts compromised oligodendrocyte differentiation. Brain Pathol. 2018;28:902–19. 29476661 10.1111/bpa.12595 PMC6857458

[357] Berger J, Forss-Petter S, Eichler FS. Pathophysiology of X-linked adrenoleukodystrophy. Biochimie. 2014;98:135–42. 24316281 10.1016/j.biochi.2013.11.023 PMC3988840

[358] Hashemi E, Narain Srivastava I, Aguirre A, Tilahan Yoseph E, Kaushal E, Awani A, et al. A novel mouse model of cerebral adrenoleukodystrophy highlights NLRP3 activity in lesion pathogenesis. bioRxiv. 2023.11.07.564025.

[359] Hashemi E, Srivastava IN, Aguirre A, Yoseph ET, Kaushal E, Awani A, et al. A novel mouse model for cerebral inflammatory demyelination in X-Linked Adrenoleukodystrophy: Insights into pathogenesis and potential therapeutic targets. Ann Neurol. 2025;97:296–312. 39467011 10.1002/ana.27117 PMC11747894

[360] Kipp M, Clarner T, Dang J, Copray S, Beyer C. The cuprizone animal model: new insights into an old story. Acta Neuropathol. 2009;118:723–36. 19763593 10.1007/s00401-009-0591-3

[361] Chang SC, Eichinger CS, Field P. The natural history and burden of illness of metachromatic leukodystrophy: a systematic literature review. Eur J Med Res. 2024;29:181. 38494502 10.1186/s40001-024-01771-1 PMC10946116

[362] van Rappard DF, Boelens JJ, Wolf NI. Metachromatic leukodystrophy: Disease spectrum and approaches for treatment. Best Pr Res Clin Endocrinol Metab. 2015;29:261–73. 10.1016/j.beem.2014.10.001 25987178

[363] Mekhaeil M, Conroy MJ, Dev KK. Olaparib attenuates demyelination and neuroinflammation in an organotypic slice culture model of metachromatic leukodystrophy. Neurotherapeutics. 2023;20:1347–68. 37525026 10.1007/s13311-023-01409-w PMC10480139

[364] Audouard E, Khefif N, Mansat C, Nelcha O, Banchi EG, Lupiet C, et al. Dose-response evaluation of intravenous gene therapy in a symptomatic mouse model of metachromatic leukodystrophy. Mol Ther Methods Clin Dev. 2024;32:101248. 38680552 10.1016/j.omtm.2024.101248 PMC11046302

[365] Hess B, Saftig P, Hartmann D, Coenen R, Lüllmann-Rauch R, Goebel HH, et al. Phenotype of arylsulfatase A-deficient mice: relationship to human metachromatic leukodystrophy. Proc Natl Acad Sci USA. 1996;93:14821–6. 8962139 10.1073/pnas.93.25.14821 PMC26220

[366] Givogri MI, Galbiati F, Fasano S, Amadio S, Perani L, Superchi D, et al. Oligodendroglial progenitor cell therapy limits central neurological deficits in mice with metachromatic leukodystrophy. J Neurosci. 2006;26:3109–19. 16554462 10.1523/JNEUROSCI.4366-05.2006 PMC6674100

[367] St Martin T, Seabrook TA, Gall K, Newman J, Avila N, Hayes A, et al. Single systemic administration of a gene therapy leading to disease treatment in metachromatic leukodystrophy Arsa knock-out mice. J Neurosci. 2023;43:3567–81. 36977578 10.1523/JNEUROSCI.1829-22.2023 PMC10184740

[368] Miyake N, Miyake K, Sakai A, Yamamoto M, Suzuki H, Shimada T. Treatment of adult metachromatic leukodystrophy model mice using intrathecal administration of type 9 AAV vector encoding arylsulfatase A. Sci Rep. 2021;11:20513. 34654893 10.1038/s41598-021-99979-2 PMC8521568

[369] Beerepoot S, Nierkens S, Boelens JJ, Lindemans C, Bugiani M, Wolf NI. Peripheral neuropathy in metachromatic leukodystrophy: current status and future perspective. Orphanet J Rare Dis. 2019;14:240. 31684987 10.1186/s13023-019-1220-4 PMC6829806

[370] Shaimardanova AA, Chulpanova DS, Solovyeva VV, Mullagulova AI, Kitaeva KV, Allegrucci C, et al. Metachromatic Leukodystrophy: Diagnosis, modeling, and treatment approaches. Front Med. 2020;7:576221. 10.3389/fmed.2020.576221 PMC7606900 33195324

[371] Ramakrishnan H, Hedayati KK, Lüllmann-Rauch R, Wessig C, Fewou SN, Maier H, et al. Increasing sulfatide synthesis in myelin-forming cells of arylsulfatase A-deficient mice causes demyelination and neurological symptoms reminiscent of human metachromatic leukodystrophy. J Neurosci. 2007;27:9482–90. 17728461 10.1523/JNEUROSCI.2287-07.2007 PMC6673125

[372] Joutel A, Monet-Leprêtre M, Gosele C, Baron-Menguy C, Hammes A, Schmidt S, et al. Cerebrovascular dysfunction and microcirculation rarefaction precede white matter lesions in a mouse genetic model of cerebral ischemic small vessel disease. J Clin Invest. 2010;120:433–45. 20071773 10.1172/JCI39733 PMC2810078

[373] Joutel A, Vahedi K, Corpechot C, Troesch A, Chabriat H, Vayssière C, et al. Strong clustering and stereotyped nature of Notch3 mutations in CADASIL patients. Lancet. 1997;350:1511–5. 9388399 10.1016/S0140-6736(97)08083-5

[374] Cognat E, Cleophax S, Domenga-Denier V, Joutel A. Early white matter changes in CADASIL: evidence of segmental intramyelinic oedema in a pre-clinical mouse model. Acta Neuropathol Commun. 2014;2:49. 24886907 10.1186/2051-5960-2-49 PMC4035092

[375] Rutten JW, Klever RR, Hegeman IM, Poole DS, Dauwerse HG, Broos LA, et al. The NOTCH3 score: a pre-clinical CADASIL biomarker in a novel human genomic NOTCH3 transgenic mouse model with early progressive vascular NOTCH3 accumulation. Acta Neuropathol Commun. 2015;3:89. 26715087 10.1186/s40478-015-0268-1 PMC4696336

[376] Ruchoux MM, Domenga V, Brulin P, Maciazek J, Limol S, Tournier-Lasserve E, et al. Transgenic mice expressing mutant Notch3 develop vascular alterations characteristic of cerebral autosomal dominant arteriopathy with subcortical infarcts and leukoencephalopathy. Am J Pathol. 2003;162:329–42. 12507916 10.1016/S0002-9440(10)63824-2 PMC1851116

[377] Ghosh M, Balbi M, Hellal F, Dichgans M, Lindauer U, Plesnila N. Pericytes are involved in the pathogenesis of cerebral autosomal dominant arteriopathy with subcortical infarcts and leukoencephalopathy. Ann Neurol. 2015;78:887–900. 26312599 10.1002/ana.24512

[378] Kastberger B, Winter S, Brandstätter H, Biller J, Wagner W, Plesnila N. Treatment with Cerebrolysin prolongs lifespan in a mouse model of cerebral autosomal dominant Arteriopathy with Subcortical Infarcts and Leukoencephalopathy. Adv Biol. 2024;8:e2300439. 10.1002/adbi.202300439 38062874

[379] Rajani RM, Dupré N, Domenga-Denier V, Van Niel G, Heiligenstein X, Joutel A. Characterisation of early ultrastructural changes in the cerebral white matter of CADASIL small vessel disease using high-pressure freezing/freeze-substitution. Neuropathol Appl Neurobiol. 2021;47:694–704. 33483954 10.1111/nan.12697

[380] Alexander WS. Progressive fibrinoid degeneration of fibrillary astrocytes associated with mental retardation in a hydrocephalic infant. Brain. 1949;72:373–81. 15409268 10.1093/brain/72.3.373

[381] Messing A, Head MW, Galles K, Galbreath EJ, Goldman JE, Brenner M. Fatal encephalopathy with astrocyte inclusions in GFAP transgenic mice. Am J Pathol. 1998;152:391–8. 9466565 PMC1857948

[382] Tanaka KF, Takebayashi H, Yamazaki Y, Ono K, Naruse M, Iwasato T, et al. Murine model of Alexander disease: analysis of GFAP aggregate formation and its pathological significance. Glia. 2007;55:617–31. 17299771 10.1002/glia.20486

[383] Hagemann TL, Connor JX, Messing A. Alexander disease-associated glial fibrillary acidic protein mutations in mice induce Rosenthal fiber formation and a white matter stress response. J Neurosci. 2006;26:11162–73. 17065456 10.1523/JNEUROSCI.3260-06.2006 PMC6674663

[384] Pekny T, Faiz M, Wilhelmsson U, Curtis MA, Matej R, Skalli O, et al. Synemin is expressed in reactive astrocytes and Rosenthal fibers in Alexander disease. APMIS. 2014;122:76–80. 23594359 10.1111/apm.12088

[385] Cho ES, Han S, Kim G, Eom M, Lee KH, Kim CH, et al. In vivo whole-brain imaging of zebrafish larvae using three-dimensional fluorescence microscopy. J Vis Exp. 2023;194:65218. 10.3791/65218 37184275

[386] Choe CP, Choi SY, Kee Y, Kim MJ, Kim SH, Lee Y, et al. Transgenic fluorescent zebrafish lines that have revolutionized biomedical research. Lab Anim Res. 2021;37:26. 34496973 10.1186/s42826-021-00103-2 PMC8424172

[387] Masson MA, Nait-Oumesmar B. Emerging concepts in oligodendrocyte and myelin formation, inputs from the zebrafish model. Glia. 2023;71:1147–63. 36645033 10.1002/glia.24336

[388] Münzel EJ, Becker CG, Becker T, Williams A. Zebrafish regenerate full thickness optic nerve myelin after demyelination, but this fails with increasing age. Acta Neuropathol Commun. 2014;2:77. 25022486 10.1186/s40478-014-0077-y PMC4164766

[389] Okumura G, Iguchi-Manaka A, Murata R, Yamashita-Kanemaru Y, Shibuya A, Shibuya K. Tumor-derived soluble CD155 inhibits DNAM-1-mediated antitumor activity of natural killer cells. J Exp Med. 2020;217:1. 32040157 10.1084/jem.20191290 PMC7144518

[390] Morris AD, Kucenas S. A Novel Lysolecithin model for visualizing damage in vivo in the larval Zebrafish spinal cord. Front Cell Dev Biol. 2021;9:654583. 34095120 10.3389/fcell.2021.654583 PMC8173112

[391] Karttunen MJ, Lyons DA. A drug-inducible transgenic Zebrafish model for myelinating glial cell ablation. Methods Mol Biol. 2019;1936:227–38. 30820902 10.1007/978-1-4939-9072-6_13

[392] Chung AY, Kim PS, Kim S, Kim E, Kim D, Jeong I, et al. Generation of demyelination models by targeted ablation of oligodendrocytes in the zebrafish CNS. Mol Cells. 2013;36:82–87. 23807048 10.1007/s10059-013-0087-9 PMC3887923

[393] Fang Y, Lei X, Li X, Chen Y, Xu F, Feng X, et al. A novel model of demyelination and remyelination in a GFP-transgenic zebrafish. Biol Open. 2014;4:62–68. 25527642 10.1242/bio.201410736 PMC4295166

[394] Auer F, Vagionitis S, Czopka T. Evidence for Myelin sheath remodeling in the CNS Revealed by In Vivo Imaging. Curr Biol. 2018;28:549–559.e543. 29429620 10.1016/j.cub.2018.01.017

[395] Kulkarni P, Yellanki S, Medishetti R, Sriram D, Saxena U, Yogeeswari P. Novel Zebrafish EAE model: A quick in vivo screen for multiple sclerosis. Mult Scler Relat Disord. 2017;11:32–39. 28104252 10.1016/j.msard.2016.11.010

[396] Gilardi C, Kalebic N. The Ferret as a model system for neocortex development and evolution. Front Cell Dev Biol. 2021;9:661759. 33996819 10.3389/fcell.2021.661759 PMC8118648

[397] Reillo I, de Juan Romero C, García-Cabezas M, Borrell V. A role for intermediate radial glia in the tangential expansion of the mammalian cerebral cortex. Cereb Cortex. 2011;21:1674–94. 21127018 10.1093/cercor/bhq238

[398] Wood T, Moralejo D, Corry K, Snyder JM, Traudt C, Curtis C, et al. A Ferret Model of Encephalopathy of prematurity. Dev Neurosci. 2018;40:475–89. 31079096 10.1159/000498968 PMC6658350

[399] Di Curzio DL, Buist RJ, Del Bigio MR. Reduced subventricular zone proliferation and white matter damage in juvenile ferrets with kaolin-induced hydrocephalus. Exp Neurol. 2013;248:112–28. 23769908 10.1016/j.expneurol.2013.06.004

[400] Chavali M, Ulloa-Navas MJ, Pérez-Borredá P, Garcia-Verdugo JM, McQuillen PS, Huang EJ, et al. Wnt-Dependent Oligodendroglial-endothelial interactions regulate white matter vascularization and attenuate injury. Neuron. 2020;108:1130–1145.e1135. 33086038 10.1016/j.neuron.2020.09.033 PMC7769920

[401] Walter C, Balouchzadeh R, Garcia KE, Kroenke CD, Pathak A, Bayly PV. Multi-scale measurement of stiffness in the developing ferret brain. Sci Rep. 2023;13:20583. 37996465 10.1038/s41598-023-47900-4 PMC10667369

[402] Schwerin SC, Chatterjee M, Imam-Fulani AO, Radomski KL, Hutchinson EB, Pierpaoli CM, et al. Progression of histopathological and behavioral abnormalities following mild traumatic brain injury in the male ferret. J Neurosci Res. 2018;96:556–72. 29360208 10.1002/jnr.24218

[403] Krieg JL, Leonard AV, Tuner RJ, Corrigan F. Characterization of traumatic brain injury in a Gyrencephalic Ferret Model Using the Novel Closed Head Injury Model of Engineered Rotational Acceleration (CHIMERA). Neurotrauma Rep. 2023;4:761–80. 38028274 10.1089/neur.2023.0047 PMC10659026

[404] Kohama SG, Rosene DL, Sherman LS. Age-related changes in human and non-human primate white matter: from myelination disturbances to cognitive decline. Age. 2012;34:1093–110. 22203458 10.1007/s11357-011-9357-7 PMC3448998

[405] Aggarwal N, Moody JF, Dean DC, Tromp DPM, Kecskemeti SR, Oler JA, et al. Spatiotemporal dynamics of nonhuman primate white matter development during the first year of life. Neuroimage. 2021;231:117825. 33549752 10.1016/j.neuroimage.2021.117825 PMC8154685

[406] Sanchez MM, McCormack KM, Howell BR. Social buffering of stress responses in nonhuman primates: Maternal regulation of the development of emotional regulatory brain circuits. Soc Neurosci. 2015;10:512–26. 26324227 10.1080/17470919.2015.1087426 PMC4618704

[407] Howell BR, McCormack KM, Grand AP, Sawyer NT, Zhang X, Maestripieri D, et al. Brain white matter microstructure alterations in adolescent rhesus monkeys exposed to early life stress: associations with high cortisol during infancy. Biol Mood Anxiety Disord. 2013;3:21. 24289263 10.1186/2045-5380-3-21 PMC3880213

[408] t Hart BA, Bauer J, Brok HP, Amor S. Non-human primate models of experimental autoimmune encephalomyelitis: Variations on a theme. J Neuroimmunol. 2005;168:1–12. 16023737 10.1016/j.jneuroim.2005.05.017

[409] t Hart BA, Laman JD, Bauer J, Blezer E, van Kooyk Y, Hintzen RQ. Modelling of multiple sclerosis: lessons learned in a non-human primate. Lancet Neurol. 2004;3:588–97. 15380155 10.1016/S1474-4422(04)00879-8

[410] Li S, Rao JH, Lan XY, Li X, Chu CY, Liang Y, et al. White matter demyelination predates axonal injury after ischemic stroke in cynomolgus monkeys. Exp Neurol. 2021;340:113655. 33617887 10.1016/j.expneurol.2021.113655

[411] Higo N. Non-human primate models to explore the adaptive mechanisms after stroke. Front Syst Neurosci. 2021;15:760311. 34819842 10.3389/fnsys.2021.760311 PMC8606408

[412] Kinder HA, Baker EW, West FD. The pig as a preclinical traumatic brain injury model: current models, functional outcome measures, and translational detection strategies. Neural Regen Res. 2019;14:413–24. 30539807 10.4103/1673-5374.245334 PMC6334610

[413] Kuang VH, Skoven CS, Arvin S, Fitting LM, Drasbek KR, Hansen B, et al. A large animal model for focal stroke: Photothrombotic lesion in the cortex of Danish Landrace pigs. J Neurosci Methods. 2025;418:110408. 40010647 10.1016/j.jneumeth.2025.110408

[414] Ryan MC, Sherman P, Rowland LM, Wijtenburg SA, Acheson A, Fieremans E, et al. Miniature pig model of human adolescent brain white matter development. J Neurosci Methods. 2018;296:99–108. 29277719 10.1016/j.jneumeth.2017.12.017 PMC5817010

[415] Back SA, Riddle A, Dean J, Hohimer AR. The instrumented fetal sheep as a model of cerebral white matter injury in the premature infant. Neurotherapeutics. 2012;9:359–70. 22399133 10.1007/s13311-012-0108-y PMC3337024

[416] Silbereis JC, Huang EJ, Back SA, Rowitch DH. Towards improved animal models of neonatal white matter injury associated with cerebral palsy. Dis Model Mech. 2010;3:678–88. 21030421 10.1242/dmm.002915 PMC2965396

[417] Banstola A, Reynolds JNJ. The Sheep as a Large Animal Model for the Investigation and Treatment of Human Disorders. Biology (Basel). 2022;11:1251. 10.3390/biology11091251 PMC9495394 36138730

[418] Yuen TJ, Silbereis JC, Griveau A, Chang SM, Daneman R, Fancy SPJ, et al. Oligodendrocyte-encoded HIF function couples postnatal myelination and white matter angiogenesis. Cell. 2014;158:383–96. 25018103 10.1016/j.cell.2014.04.052 PMC4149873

[419] Templeton JP, Geisert EE. A practical approach to optic nerve crush in the mouse. Mol Vis. 2012;18:2147–52. 22876142 PMC3413441

[420] Xing J, Lukomska A, Rheaume BA, Kim J, Sajid MS, Damania A, et al. Post-injury born oligodendrocytes incorporate into the glial scar and contribute to the inhibition of axon regeneration. Development. 2023;150:dev201311. 10.1242/dev.201311 PMC10163352 36971369

[421] Mendonça HR, Villas Boas COG, Heringer LDS, Oliveira JT, Martinez AMB. Myelination of regenerating optic nerve axons occurs in conjunction with an increase of the oligodendrocyte precursor cell population in the adult mice. Brain Res Bull. 2021;166:150–60. 33232742 10.1016/j.brainresbull.2020.11.012

[422] Rodriguez JP, Coulter M, Miotke J, Meyer RL, Takemaru K, Levine JM. Abrogation of β-catenin signaling in oligodendrocyte precursor cells reduces glial scarring and promotes axon regeneration after CNS injury. J Neurosci. 2014;34:10285–97. 25080590 10.1523/JNEUROSCI.4915-13.2014 PMC4115138

[423] Hawker B, Dhakal M, Connor B, McCaughey-Chapman A. Modeling demyelination and endogenous remyelination in spinal cord ex vivo rat organotypic slice cultures. Front Cell Neurosci. 2024;18:1345042. 38988661 10.3389/fncel.2024.1345042 PMC11233765

[424] Sekizar S, Williams A. Ex vivo slice cultures to study myelination, demyelination, and remyelination in mouse brain and spinal cord. Methods Mol Biol. 2019;1936:169–83. 30820899 10.1007/978-1-4939-9072-6_10

[425] Shen K, Yuen TJ. Ex vivo myelination and remyelination in cerebellar slice cultures as a quantitative model for developmental and disease-relevant manipulations. J Vis Exp. 2020;160:61044. 10.3791/61044 32597864

[426] Doussau F, Dupont JL, Neel D, Schneider A, Poulain B, Bossu JL. Organotypic cultures of cerebellar slices as a model to investigate demyelinating disorders. Expert Opin Drug Discov. 2017;12:1011–22. 28712329 10.1080/17460441.2017.1356285

[427] Baudouin L, Adès N, Kanté K, Czarnecki A, Bachelin C, Baskaran A, et al. Co-culture of exogenous oligodendrocytes with unmyelinated cerebella: Revisiting ex vivo models and new tools to study myelination. Glia. 2021;69:1916–31. 33811384 10.1002/glia.24001

[428] Manousi A, Göttle P, Reiche L, Cui QL, Healy LM, Akkermann R, et al. Identification of novel myelin repair drugs by modulation of oligodendroglial differentiation competence. EBioMedicine. 2021;65:103276. 33714029 10.1016/j.ebiom.2021.103276 PMC7970057

[429] Najm FJ, Madhavan M, Zaremba A, Shick E, Karl RT, Factor DC, et al. Drug-based modulation of endogenous stem cells promotes functional remyelination in vivo. Nature. 2015;522:216–20. 25896324 10.1038/nature14335 PMC4528969

[430] Yao X, Su T, Verkman AS. Clobetasol promotes remyelination in a mouse model of neuromyelitis optica. Acta Neuropathol Commun. 2016;4:42. 27117475 10.1186/s40478-016-0309-4 PMC4845317

[431] Plug BC, Revers IM, Breur M, González GM, Timmerman JA, Meijns NRC, et al. Human post-mortem organotypic brain slice cultures: a tool to study pathomechanisms and test therapies. Acta Neuropathol Commun. 2024;12:83. 38822428 10.1186/s40478-024-01784-1 PMC11140981

[432] James OG, Selvaraj BT, Magnani D, Burr K, Connick P, Barton SK, et al. iPSC-derived myelinoids to study myelin biology of humans. Dev Cell. 2021;56:1346–1358.e1346. 33945785 10.1016/j.devcel.2021.04.006 PMC8098746

[433] Marangon D, Caporale N, Boccazzi M, Abbracchio MP, Testa G, Lecca D. Novel in vitro experimental approaches to study myelination and remyelination in the central nervous system. Front Cell Neurosci. 2021;15:748849. 34720882 10.3389/fncel.2021.748849 PMC8551863

[434] Kawabata S, Takano M, Numasawa-Kuroiwa Y, Itakura G, Kobayashi Y, Nishiyama Y, et al. Grafted human iPS cell-derived Oligodendrocyte precursor cells contribute to robust remyelination of demyelinated axons after spinal cord injury. Stem Cell Rep. 2016;6:1–8. 10.1016/j.stemcr.2015.11.013 PMC4719132 26724902

[435] Martinez-Curiel R, Jansson L, Tsupykov O, Avaliani N, Aretio-Medina C, Hidalgo I, et al. Oligodendrocytes in human induced pluripotent stem cell-derived cortical grafts remyelinate adult rat and human cortical neurons. Stem Cell Rep. 2023;18:1643–56. 10.1016/j.stemcr.2023.04.010 PMC10444570 37236198

[436] Vandana JJ, Manrique C, Lacko LA, Chen S. Human pluripotent-stem-cell-derived organoids for drug discovery and evaluation. Cell Stem Cell. 2023;30:571–91. 37146581 10.1016/j.stem.2023.04.011 PMC10775018

[437] Park JC, Jang SY, Lee D, Lee J, Kang U, Chang H, et al. A logical network-based drug-screening platform for Alzheimer’s disease representing pathological features of human brain organoids. Nat Commun. 2021;12:280. 33436582 10.1038/s41467-020-20440-5 PMC7804132

[438] Osaki T, Uzel SGM, Kamm RD. Microphysiological 3D model of amyotrophic lateral sclerosis (ALS) from human iPS-derived muscle cells and optogenetic motor neurons. Sci Adv. 2018;4:eaat5847. 30324134 10.1126/sciadv.aat5847 PMC6179377

[439] GBD 2021 Diseases and Injuries Collaborators. Global incidence, prevalence, years lived with disability (YLDs), disability-adjusted life-years (DALYs), and healthy life expectancy (HALE) for 371 diseases and injuries in 204 countries and territories and 811 subnational locations, 1990-2021: a systematic analysis for the Global Burden of Disease Study 2021. Lancet 2024;403**:**2133–61. 10.1016/S0140-6736(24)00757-8 PMC11122111 38642570

[440] Caloni F, De Angelis I, Hartung T. Replacement of animal testing by integrated approaches to testing and assessment (IATA): a call for in vivitrosi. Arch Toxicol. 2022;96:1935–50. 35503372 10.1007/s00204-022-03299-x PMC9151502

[441] Rai J, Kaushik K. Reduction of animal sacrifice in biomedical science & research through alternative design of animal experiments. Saudi Pharm J. 2018;26:896–902. 30202234 10.1016/j.jsps.2018.03.006 PMC6128677

[442] Jankowsky JL, Fadale DJ, Anderson J, Xu GM, Gonzales V, Jenkins NA, et al. Mutant presenilins specifically elevate the levels of the 42 residue beta-amyloid peptide in vivo: evidence for augmentation of a 42-specific gamma secretase. Hum Mol Genet. 2004;13:159–70. 14645205 10.1093/hmg/ddh019

[443] Liu Y, Xue X, Zhang H, Che X, Luo J, Wang P, et al. Neuronal-targeted TFEB rescues dysfunction of the autophagy-lysosomal pathway and alleviates ischemic injury in permanent cerebral ischemia. Autophagy. 2019;15:493–509. 30304977 10.1080/15548627.2018.1531196 PMC6351122

[444] Schleicher C, Starker K, Burkhardt B, Starker W. Value of hysterosalpingography in the diagnosis of sterility. Radio Diagn. 1987;28:657–67. 3423247

